# Bioinformatics Identification and Expression Analysis of Acetyl-CoA Carboxylase Reveal Its Role in Isoflavone Accumulation during Soybean Seed Development

**DOI:** 10.3390/ijms251810221

**Published:** 2024-09-23

**Authors:** Xu Wu, Zhenhong Yang, Yina Zhu, Yuhang Zhan, Yongguang Li, Weili Teng, Yingpeng Han, Xue Zhao

**Affiliations:** Key Laboratory of Soybean Biology in Chinese Education Ministry, Northeast Agricultural University, Harbin 150030, China; wuxubio@163.com (X.W.); dongyzh918@163.com (Z.Y.); 15045389452@163.com (Y.Z.); zyhsoybean@163.com (Y.Z.); yongguangli@neau.edu.cn (Y.L.); twlneau@163.com (W.T.)

**Keywords:** soybean (*Glycine max* L. Merr.), Acetyl-CoA carboxylase (ACCase), gene family, phylogenetic analysis, gene expression, WGCNA

## Abstract

Isoflavones belong to the class of flavonoid compounds, which are important secondary metabolites that play a crucial role in plant development and defense. Acetyl-CoA carboxylase (ACCase) is a biotin-dependent enzyme that catalyzes the conversion of Acetyl-CoA into Malonyl-CoA in plants. It is a key enzyme in fatty acid synthesis and also catalyzes the production of various secondary metabolites. However, information on the *ACC* gene family in the soybean (*Glycine max* L. Merr.) genome and the specific members involved in isoflavone biosynthesis is still lacking. In this study, we identified 20 *ACC* family genes (*GmACCs*) from the soybean genome and further characterized their evolutionary relationships and expression patterns. Phylogenetic analysis showed that the *GmACCs* could be divided into five groups, and the gene structures within the same groups were highly conserved, indicating that they had similar functions. The *GmACCs* were randomly distributed across 12 chromosomes, and collinearity analysis suggested that many *GmACCs* originated from tandem and segmental duplications, with these genes being under purifying selection. In addition, gene expression pattern analysis indicated that there was functional divergence among *GmACCs* in different tissues. The *GmACCs* reached their peak expression levels during the early or middle stages of seed development. Based on the transcriptome and isoflavone content data, a weighted gene co-expression network was constructed, and three candidate genes (*Glyma.06G105900*, *Glyma.13G363500*, and *Glyma.13G057400*) that may positively regulate isoflavone content were identified. These results provide valuable information for the further functional characterization and application of *GmACCs* in isoflavone biosynthesis in soybean.

## 1. Introduction

Soybean (*Glycine max* L. Merr.), anciently known as “shu”, is an annual leguminous crop native to Asia with a cultivation history of thousands of years in China. As an important crop for both food and feed, soybeans play a significant role in agricultural production and the diet of residents [1]. Soybeans are a good source of bioactive components such as vegetable oil, protein, and isoflavones [2]. Isoflavones are significant secondary metabolites in the phenylpropanoid biosynthesis pathway [3], predominantly found in legumes, and are richly present in soybean seeds, constituting from 0.1% to 0.6% of the seed mass [4]. Soybean isoflavones include daidzein (DZ), genistein (GT), glycitein (GC), daidzin, genistin, glycitin, 6-o-acetyldaidzin, 6-o-acetylgenistin, 6-o-acetylglycitin, 6-o-malonyldaidzin, 6-o-malonylgenistin, and 6-o-malonylglycitin [5]. Among them, DZ, GT, and GC are the major bioactive components in human nutrition [6]. Soybeans are the only effective source of isoflavones for humans [7]. Isoflavones are precursors in the biosynthesis of phytoalexins [8] and play a crucial role in the defensive responses of plants to various biological and abiotic stresses [9]. Additionally, isoflavones possess biological activity and serve as natural plant estrogens, playing a significant role in healthcare, wellness, and beauty applications [10]. The pathway of isoflavone biosynthesis is relatively complex and is regulated by a variety of genes and metabolic networks [11]. This pathway primarily involves enzymes such as phenylalanine ammonia lyase (PAL), chalcone synthase (CHS), chalcone isomerase (CHI), and isoflavone synthase (IFS). Among them, chalcone synthase (CHS) and isoflavone synthase (IFS) are key enzymes in the production of isoflavones. To date, a large number of genes involved in the isoflavone biosynthetic pathway have been elucidated. According to reports, a total of 12 chalcone isomerase (CHI) family members [12,13], 21 chalcone synthase (CHS) family members [14], and 14 chalcone reductase (CHR) family members have been identified in soybeans [15]. Therefore, studying the different gene families in soybeans will contribute to our understanding of their evolution and functions.

Acetyl-CoA carboxylase (ACCase) is a biotin-dependent enzyme that catalyzes the conversion of Acetyl-CoA into Malonyl-CoA, playing a crucial role in the fatty acid synthesis pathway [16,17]. Acetyl-CoA carboxylase consists of the three following domains: biotin carboxylase (BC), carboxyl transferase (CT), and biotin carboxyl carrier protein (BCCP) [18]. In plants, ACCase is primarily classified into the two following types: homomeric and heteromeric. The heteromeric form of ACCase, also known as the prokaryotic type, is mainly found in the plastids of plants [19]. The heteromeric form of Acetyl-CoA carboxylase is a multi-subunit complex consisting of the four following subunits: BC, BCCP, α-CT, and β-CT [20]. In its active state, the BC and BCCP subunits form homodimers, while the α-CT and β-CT subunits exist as a heterodimer. The α-CT and β-CT subunits are connected by a covalent bond and, together, they constitute the CT catalytic domain [21]. The BC subunit, BCCP subunit, and α-CT subunit are encoded by the nuclear genes *accC*, *accB*, and *accA*, respectively, while the β-CT subunit is encoded by the chloroplast *accD* gene [22]. The BC, BCCP, and α-CT subunits possess N-terminal signal peptide sequences. Their protein precursors are synthesized in the cytoplasm and then transported into the chloroplasts. After the signal peptide is removed, the protein matures. This mature protein then associates with the β-CT subunit to form a functional, complete protein [23]. The heteromeric form of ACCase is structurally unstable and prone to dissociation. In contrast, the homomeric form of ACCase is a single polypeptide chain that forms a stable homodimer within cells, which is difficult to dissociate [24]. The homomeric form of ACCase, also known as the eukaryotic type, is primarily found in the cytoplasm of algae and higher plants [25]. In ACCase, the three functional domains of the homomeric form are homologous in sequence to the four subunits of the heteromeric form [26]. So far, *ACC* genes have been identified in various species, and the number of *ACC* genes varies greatly among different species. Generally, in most plants, the genes encoding the BCCP subunit of *ACC* are the most numerous [27]. In five Brassica species, 43 BCCP subunits were identified, and some of these genes may play important roles in their responses to biotic and abiotic stresses [28]. In four cotton varieties, 24 *BCCP* genes were identified. In each variety, the homologs exhibited relative conservation in their gene structure and motifs, and they were induced by cold or salt stress signals, showing specific expressions in different tissue locations [29].

In addition to being involved in de novo fatty acid synthesis, some studies have reported that ACCase is also important for secondary metabolism [30]. From a metabolic pathway perspective, ACCase catalyzes the conversion of Acetyl-CoA into Malonyl-CoA. Malonyl-CoA, serving as a necessary substrate in the flavonoid metabolic pathway, and is combined with p-coumaroyl CoA and catalyzed by chalcone synthase (CHS) and chalcone reductase (CHR), ultimately resulting in the production of isoflavone synthesis precursors such as naringenin and liquiritigenin [31]. Research has reported that ACCase can influence the synthesis of flavonoid compounds. In *Arabidopsis thaliana*, overexpression of the *BCCP* gene led to the suppression of plastidial ACCase and increased the production of cytoplasmic ACCase to promote the up-regulation of flavonoid biosynthesis genes (*At1g53520*, *At3g51240*, and *At1g06000*) [32]. In rapeseed leaves, the activity of homomeric ACCase was reduced by antisense RNA technology, which could decrease the accumulation of flavonoids under UV-B treatment [33]. In *Escherichia coli*, the co-overexpression of the four ACCase subunits led to an increase in the production of flavonoid compounds [34].

*GmACCs* in the soybean genome and their regulatory roles, especially in the flavonoid and isoflavone biosynthesis pathway, have not been widely studied. In the present study, we identified 20 *GmACCs* and constructed a comprehensive phylogenetic tree. The gene structures, conserved motifs, promoter *cis*-acting elements, collinearity, and expression patterns of the *GmACCs* were analyzed. To further elucidate the function of the *GmACCs*, we constructed a weighted gene co-expression network using transcriptomic and isoflavone content data from soybean seeds. These results provide a basic understanding of the roles and complexity of *GmACCs* in soybean and lay the foundation for understanding the breeding utilization of *GmACCs* in improving soybean isoflavone content and stress resistance.

## 2. Results

### 2.1. Identification of ACC Genes from Soybean Genome

A total of 17 *ACC* genes and 3 putative *ACC* genes were identified from the soybean genome, named *GmACC1* to *GmACC20* based on their chromosomal locations and subunit compositions (Table 1). The 20 *GmACCs* were randomly distributed across 12 chromosomes of the soybean genome. Six genes (30%) were distributed on chromosome 18, which had the largest number of *GmACCs*, while three genes (15%) were located on chromosome 15. The length of the soybean ACCase proteins ranged from 34 to 2260 amino acids and the molecular weight ranged from 3847.76 kDa to 252,370.45 kDa, with the predicted isoelectric point ranging from 4.51 to 10.30. Subcellular localization predictions suggested that GmACC1, GmACC2, and GmACC12 were primarily located in the cytoplasm, GmACC4 and GmACC15 were mainly localized in the nucleus, and GmACC3 was primarily located in the plasma membrane. Interestingly, the subcellular localization of the majority of the remaining ACCase proteins was primarily in the semi-autonomous organelles’ mitochondria and chloroplasts (Table 1 and Table S1).

### 2.2. Phylogenetic Analysis of GmACCs

**Figure 1:**
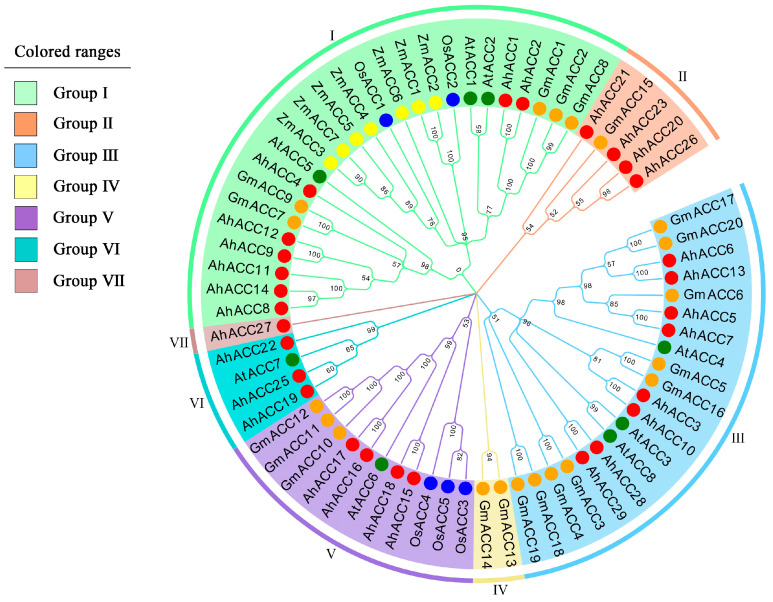
Phylogenetic analysis of *ACC* genes from soybean, *Arabidopsis thaliana*, rice, maize, and peanut. A phylogenetic tree constructed using full-length protein sequences. Different shades of color were used to distinguish different branches of Group I–VII indicating the classification of the *ACCase* gene family.

To evaluate the evolutionary relationships among the ACCase proteins, we constructed a phylogenetic tree based on the amino acid sequences of twenty *ACC* genes from soybean, eight from *Arabidopsis thaliana*, five from rice, seven from maize, and twenty-nine from peanuts (File S1 and Table S2). In plants, ACCase is primarily composed of BC, CT, and BCCP. CT further comprises α-CT and β-CT subunits. Phylogenetic analysis revealed that the 20 *GmACCs* identified in soybean belonged five different groups, respectively (Group I–Group V). Group VI and VII did not cover any *GmACCs*. The *GmACCs* encoding the BC subunit proteins were predominantly found in Group I, genes encoding the BCCP subunit proteins mainly resided in Group III, genes encoding the β-CT subunit proteins were predominantly found in both Group II and Group IV, and genes encoding the α-CT subunit proteins were mainly found in Group V (Figure 1). Overall, *ACCase* genes from soybean, *Arabidopsis thaliana*, maize, rice, and peanuts were simultaneously identified in Group I. The *GmACCs* were highly similar to those in peanuts. Additionally, seven ACCase members from maize were only found in Group I, and five ACCase members from rice were only found in Group I and Group V, suggesting that the differentiation of *ACC* genes may happen between monocotyledon and dicotyledon in the process of evolution (Figure 1).

### 2.3. Conserved Motifs and Gene Structure Analysis of GmACCs

**Figure 2:**
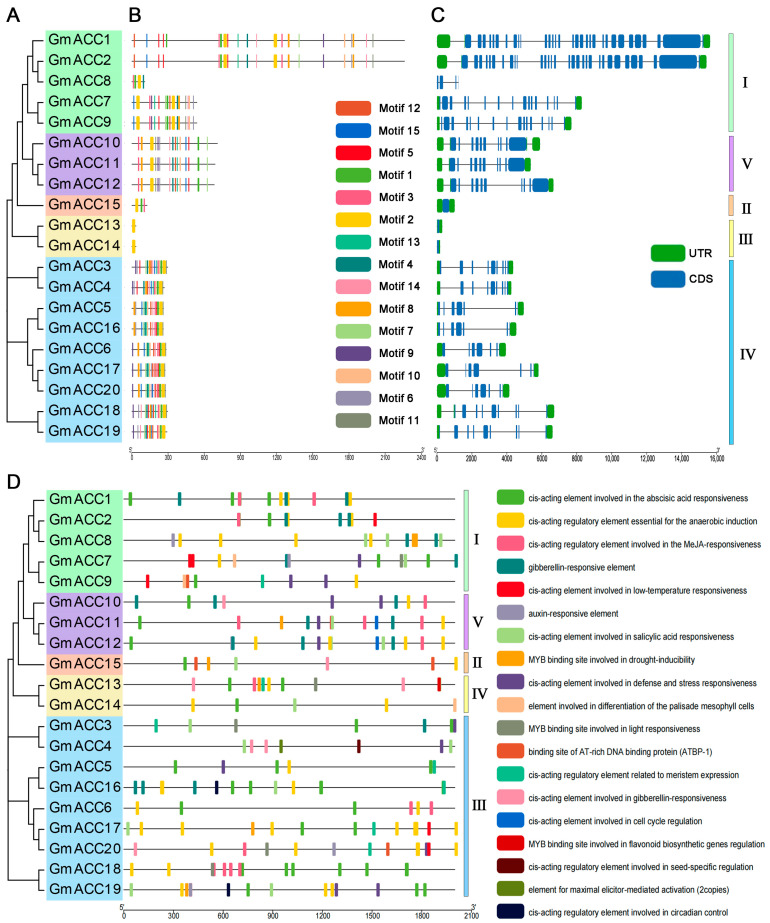
Conserved motifs, gene structure maps of 20 *GmACCs*, and *cis*-elements in the promoter sequences of *GmACCs*. (**A**) Phylogenetic tree of 20 *GmACCs*. (**B**) Motif composition of 20 *GmACCs*; the black line indicates the protein length. (**C**) Gene structures of 20 *GmACCs*. Black lines indicate introns, green boxes indicate UTR, and blue boxes indicate CDS. (**D**) Schematic model of 19 *cis*-elements in the promoter sequences of *GmACCs*.

To explore the sequence structures of the soybean *ACCase* family, we predicted the conserved motifs using the MEME tool and also analyzed the intron/exon structure of each member of the *GmACCs*. The results showed that 15 highly conserved motifs were found in the *GmACCs*. Only motif 2 was present in all 20 *GmACCs*, while other motifs differed in type and number among the *ACCase* family members. Interestingly, the chloroplast genes *GmACC13* and *GmACC14*, which encode the β-CT subunit, contained only one motif 2. There were differences in the conserved motifs among the members of the *GmACCs*, which may have also led to functional differences among the members of the gene family. Gene structure analysis revealed that almost all members of the *GmACCs* family contained a certain number of introns, except for *GmACC13*, *GmACC14*, and *GmACC15*, which lacked introns. Additionally, *GmACC8* had no untranslated regions (UTRs) at the 5′ end and 3′ end (Figure 2A–C).

### 2.4. Cis-Acting Element Analysis of GmACC Gene Promoters

Due to the important role of *cis*-elements in gene expression regulation, we analyzed the *cis*-elements in the promoters of *GmACCs* using the Plant CARE online tool. The results showed that the *cis*-regulatory elements in the promoter region of the *GmACC* genes were involved in light response, various hormone responses, growth regulation, seed development, responses to abiotic stress, and secondary metabolism processes in addition to the fundamental TATA-box and CAAT-box (Figure 2D and Table S3). In the study, MYB binding sites were present in the promoter regions of 10 *GmACC* genes (*GmACC3*, *GmACC7*, *GmACC8*, *GmACC11*, *GmACC13*, *GmACC15*, *GmACC17*, *GmACC18*, *GmACC19*, and *GmACC20*). Some research reports that certain MYB binding sites can serve as gene regulatory elements involved in flavonoid biosynthesis [35,36]. It has been hypothesized that these genes might synthesize isoflavones by regulating the upstream substances of the isoflavone biosynthesis pathway. Methyl jasmonate (MeJA) and salicylic acid (SA) also affect isoflavone biosynthesis [37,38]. The study found that 11 *GmACC* genes possessed *cis*-acting regulatory elements involved in MeJA responsiveness, while 12 genes had *cis*-acting regulatory elements involved in salicylic acid responsiveness. Apart from *GmACC5*, *GmACC9*, and *GmACC20*, these *cis*-acting elements could be identified in the *GmACCs*.

### 2.5. Collinearity and Amino Acid Substitution Selection Pressure Analyses of GmACCs

**Figure 3:**
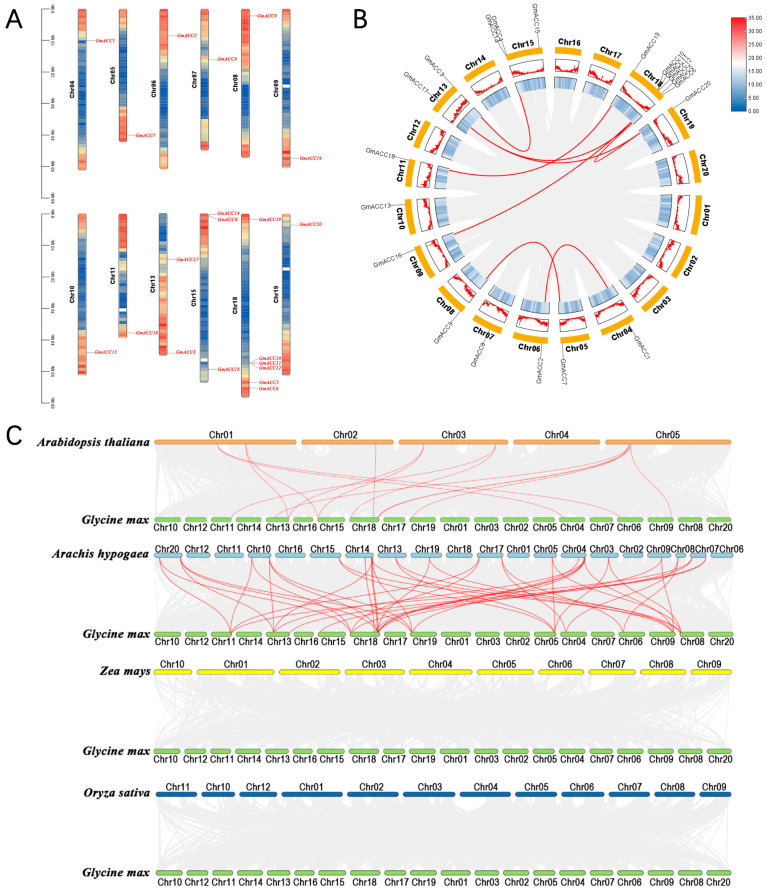
Chromosomal locations and collinearity analysis of the *ACC* genes. (**A**) Chromosomal locations the *ACC* genes. The scale is in megabases (Mbs). (**B**) The intrachromosomal segmental duplication map of the *GmACCs*. Chromosomes 01–20 are represented by yellow rectangles. Along these rectangles, blue lines and a heatmap indicate the gene density on the chromosomes. The red lines represent the segmental duplication pairs between the *GmACCs* and the gray lines represent the segmental duplication pairs in the whole soybean genome. Different colors on the chromosomes represent gene density, with red indicating high-density regions and blue indicating low-density regions. (**C**) Collinearity analysis of the *ACC* genes between soybean and four other plant species. Red lines represent the syntenic *ACC* gene pairs. Gray lines indicate collinear blocks.

We utilized soybean genome annotation information and TBtools to conduct a chromosomal distribution analysis of the *GmACCs* [39,40]. The results showed that the *GmACCs* were randomly distributed across 12 chromosomes (Figure 3A), with the majority of genes being located in regions with a high gene density (Figure 3B). Notably, chromosome 18 contained six *GmACCs* and chromosome 15 contained three *GmACCs*, with most genes being located at both ends of the soybean chromosomes (Figure 3A).

Tandem duplication and segmental duplication facilitate the expansion of new gene family members and the emergence of new functions in plant genome evolution [41,42]. In order to identify duplication events between the *GmACCs*, we conducted a collinearity analysis on the *GmACCs* using the Multiple Collinearity Scan toolkit (MCScanX) within TBtools [43]. We found eight pairs of segmental duplications, which comprised 13 genes (Figure 3B). In addition to these segmental duplications, we also identified three pairs of tandem duplications, which consisted of three genes located on chromosome 18, belonging to Group V (Figure 3A and Table 2). These genes were preserved through tandem duplication events during evolution, which might be related to specific biological functions in soybean. In total, there were 13 *GmACCs* resulting from segmental duplications, constituting 65% (13/20) of all *GmACCs*, and 3 *GmACCs* resulting from tandem duplications, accounting for 15% (3/20) of the total. The overall duplication rate of the *GmACCs* was 80%, higher than the average duplication rate of the soybean genome (75%) [44], indicating that both segmental and tandem duplication events may play important roles in the expansion of the *GmACCs* gene family.

To determine the selective evolutionary pressures on the *GmACCs* after duplication, the KaKs Calculator was used to compute the synonymous substitution rate (Ks) and nonsynonymous substitution rate (Ka) values for the duplicated *GmACC* pairs [45]. The Ka/Ks ratio for 11 pairs of *GmACC* duplication events was less than one (Table 2). Since the Ka/Ks ratio indicates selection on the gene, the duplicated *GmACCs* were under purifying selection.

Additionally, we investigated the collinearity relationships of the *GmACCs* with *ACC* genes from four representative species, including two dicots (*Arabidopsis thaliana* and peanut) and two monocots (rice and maize), to identify homologous genes (Figure 3C and Table S4). A total of 12 soybean genes exhibited collinearity with 7 *Arabidopsis thaliana* genes, and 14 soybean genes showed collinearity with 21 peanut genes. The number of homologous gene pairs between soybean and *Arabidopsis thaliana* was 14 pairs, and the number of homologous gene pairs between soybean and peanut was 44 pairs. However, no such gene pairs were identified between soybean and rice or between soybean and maize. This discrepancy may be attributed to the closer phylogenetic relationship among dicots compared to monocots. Notably, the collinearity between soybean genes and peanut genes was stronger than that observed with the other three species. This may be related to the fact that both soybean and peanut belong to legumes. Additionally, we found that a large number of *GmACCs* exhibited collinear relationships with three to four peanut genes, suggesting that these genes may play important roles in the evolution of the *ACCase* gene family (Table S4).

### 2.6. Expression Pattern Analysis of GmACCs

**Figure 4:**
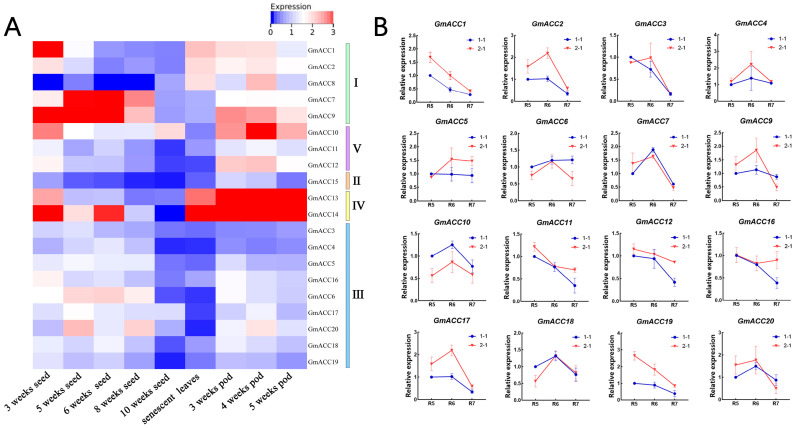
The expression profiles of *GmACCs* in soybean seeds. (**A**) The expression profilez of *GmACCs* during seed development. The figure illustrates the hierarchical clustering and heatmap of the dynamic expression levels of 20 *GmACCs* in soybean tissues. The vertical bars on the right side of the figure represent the five groups. (**B**) Relative expression of 16 *GmACCs* from seed development stages (R5–R7) detected by RT-qPCR. The blue line represents the low isoflavone variety, and the red line represents the high isoflavone variety. Each bar indicates the mean of three repeats. Similar results were obtained from three independent biological experiments.

To investigate the expression patterns of the *GmACCs* in different tissues and explore the relationships between isoflavones and the *GmACCs*, we utilized the multi-omics database SoyMD online tool to predict the expression levels of the 20 *GmACCs* in various tissues during soybean seed development [46]. The results revealed that *GmACC8* and *GmACC15* exhibited relatively low overall expression levels in seeds (Figure 4A). Moreover, most other *GmACC* genes in seeds showed an overall decreasing trend or a pattern of initially increasing and then decreasing. Members of Group II were found at higher levels in seeds and pods, while most genes in Group III showed higher expression levels in seeds, suggesting a potential association with the accumulation of fatty acids and isoflavones. Genes in Group IV exhibited significantly higher expression levels in pods than in seeds, implying that CT proteins might primarily function in pods.

Transcriptome sequencing is an effective method for analyzing gene expression patterns. Utilizing the existing soybean germplasm resources, we selected three high-isoflavone varieties and three low-isoflavone varieties. Subsequently, their seeds were subjected to transcriptome sequencing analysis at the beginning seed stage (R5), the full seed stage (R6), and the beginning maturity stage (R7). Following this, the expression levels of the 20 *GmACCs* were identified (Figure S1). The results indicated that, during the three stages of seed development, four genes (*GmACC8*, *GmACC11*, *GmACC12*, and *GmACC14*) showed no significant differences in expression between the high-isoflavone and low-isoflavone soybean varieties, suggesting that these genes are not key regulators of isoflavone synthesis. Out of the 20 *GmACCs* analyzed, 12 genes (*GmACC1*, *GmACC2*, *GmACC3*, *GmACC4*, *GmACC6*, *GmACC9*, *GmACC16*, *GmACC17*, *GmACC18*, *GmACC19*, and *GmACC20*) exhibited higher overall expression levels in the high-isoflavone varieties than in the low-isoflavone varieties. When considering the consecutive stages of seed development, the expression trends of eight genes (*GmACC2*, *GmACC3*, *GmACC4*, *GmACC6*, *GmACC7*, *GmACC9*, *GmACC17*, and *GmACC20*) showed a pattern of first increasing and then decreasing, while the expression trends of six genes exhibited a continuous decline.

To further validate the accuracy of the transcriptome data, we selected soybean varieties with significant differences in their isoflavone content through preliminary screening. We then used RT-qPCR technology to verify the expression of 16 *GmACC* genes during seed development at the R5, R6, and R7 stages (Figure 4B). The results showed that the expression levels of four genes exhibited a decreasing trend as seed development progressed. The expression levels of eight genes showed a trend of first increasing and then decreasing with seed development. Notably, the expression patterns of *GmACC2*, *GmACC4*, and *GmACC17* were essentially consistent with the transcriptomic results. Most genes showed expression trends consistent with those observed in the transcriptomic data, and the expression differences in *GmACCs* between high-isoflavone and low-isoflavone varieties were particularly evident at the R5 and R6 stages.

**Figure 5:**
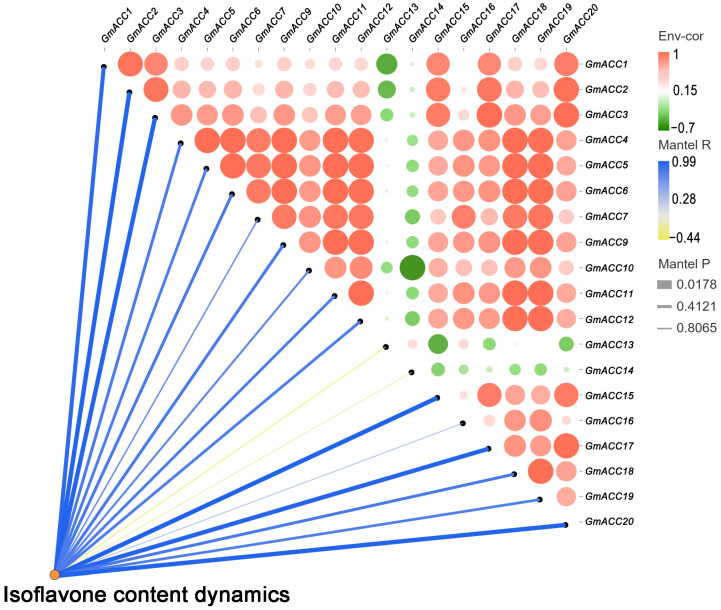
Correlation between *GmACCs* and isoflavone accumulation. The color of lines refers to Mantel’s r for statistics of corresponding distance correlations, and Edge width of lines represents the statistical significances. The size of the circles indicates the statistical significance, while the color of the circles represents the Spearman’s correlation coefficients for pairwise comparisons of *GmACC* expression levels.

To explore the correlation between isoflavone content and the expression of *GmACCs*, we randomly selected seeds from high-isoflavone varieties and collected samples at four stages over a period of 0–35 days after the R5 stage. We conducted a correlation analysis between the changes in isoflavone content and transcriptomic data at different stages (Figure 5 and Table S6). The results showed that the expression levels of most *GmACC* genes increased with the number of development days. Notably, the expression levels of five genes (*GmACC2*, *GmACC3*, *GmACC15*, *GmACC17*, and *GmACC20*) showed a significant positive correlation with the number of development days, indicating that these genes may be involved in the accumulation of isoflavones.

### 2.7. Construction and Analysis of Weighted Gene Co-Expression Networks

**Figure 6:**
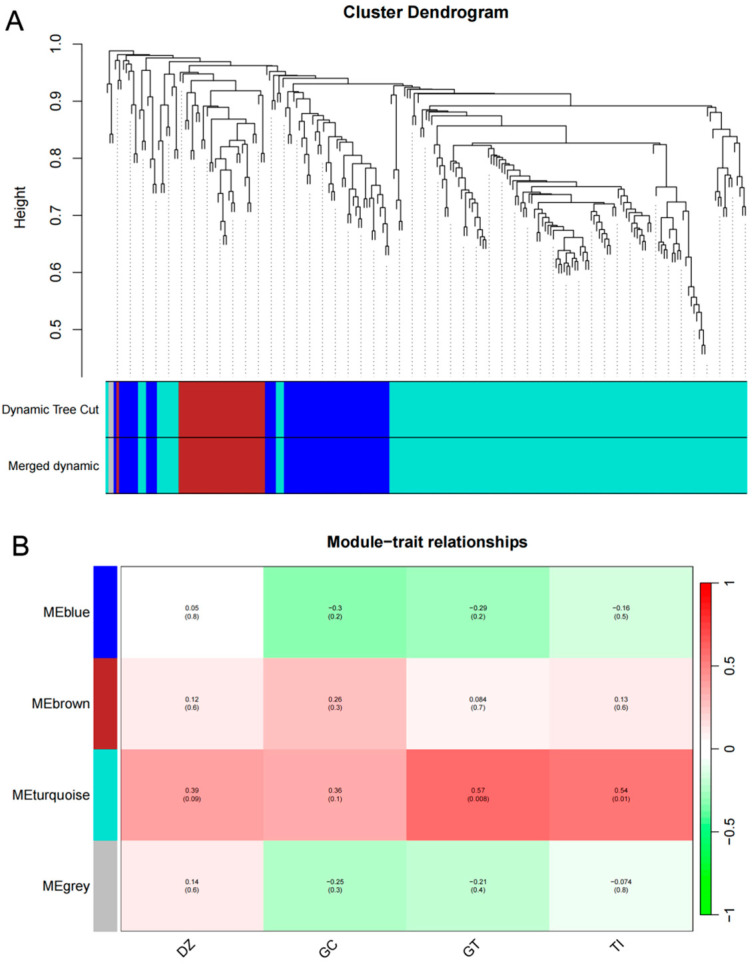
WGCNA reveals modules associated with isoflavone content. (**A**) Dendrogram of average network adjacency for identifying gene co-expression modules. (**B**) Analysis of the correlation between gene modules and traits.

To gain a more comprehensive understanding of the mechanism by which *GmACCs* regulate isoflavone biosynthesis, we selected 10 varieties with a high isoflavone content (3549.27–4688.14 µg g^−1^) and 10 varieties with a low isoflavone content (1396.33–2575.78 µg g^−1^) (Table S5). Subsequently, we conducted a transcriptome sequencing analysis on their seeds at the R6 stage. Within the established KEGG pathways, we screened 423 key genes involved in the isoflavone biosynthesis pathway and its upstream pathways, and constructed a weighted gene co-expression network based on the content data of various isoflavone components. The results showed that a total of four color modules were clustered (Figure 6), with the genes within the turquoise module showing a positive correlation with isoflavone content, especially significantly positive correlations between the genes within the module and both GT and TI. Notably, *GmACC1*, *GmACC2*, *GmACC3*, *GmACC10*, *GmACC16*, and *GmACC17* were identified in the turquoise module (Table S7). However, when integrating the transcriptomic data from different stages with the RT-qPCR results, it was found that *GmACC1*, *GmACC10*, and *GmACC16* did not show significant correlations with isoflavone accumulation. Therefore, the experiment identified the genes *GmACC2*, *GmACC3*, and *GmACC17*, which showed increased expressions from the R5 to R6 stage and were found to be synergistic with isoflavone accumulation, as candidate genes affecting isoflavone content.

## 3. Discussion

In recent years, the characterization of gene families has been instrumental in unraveling their functions. Acetyl-CoA carboxylase was first discovered in the 1960s through the study of fatty acid biosynthesis in plants [47], where the identification and characterization of this enzyme were pivotal in understanding the process of fatty acid synthesis within plants [48]. Most research on Acetyl-CoA carboxylase has primarily focused on its regulation of the de novo synthesis of fatty acids [49], while studies on its regulation of isoflavone synthesis have not yet been reported. In this study, we identified 20 *GmACC* genes that were randomly distributed across 12 chromosomes. A subcellular localization prediction analysis indicated that most of these proteins were predicted to be located in the cytoplasm, chloroplasts, and mitochondria, suggesting that this gene family could play roles in various biological processes, participating in the syntheses of certain substances and playing important roles in energy metabolism.

Phylogenetic analysis indicated that the *ACCase* genes in soybean, *Arabidopsis thaliana*, maize, rice, and peanut could be divided into seven groups, with the 20 *GmACC* genes in soybean being revealed within five of these groups. The *ACC* genes from soybean, *Arabidopsis thaliana*, maize, rice, and peanut could all be found in Group I. In this group, the identified gene family members primarily encoded homomeric ACCase and biotin carboxylase, implying that biotin carboxylase was a shared component among most plant ACCases. Furthermore, the *ACC* genes from maize and rice were exclusively identified in Group I and Group V, suggesting a possible divergence in the evolution of *ACC* genes between monocotyledonous and dicotyledonous plants. In Group IV, only two genes encoding the β-CT subunit protein were identified specifically in soybean, suggesting that this protein may have a distinct structure in soybean.

In soybean, ACCase exists in the two following forms: homomeric and heteromeric. The homomeric ACCase has the three following functional domains: the BC domain, the BCCP domain, and the CT domain. The heteromeric ACCase’s BC and BCCP domains are composed of separate BC and BCCP subunits, while the CT domain is composed of two subunits, α-CT and β-CT [50]. Studies have reported that *Arabidopsis thaliana* has three ACCase isozymes, one of which is a hetero-oligomer composed of four different subunits located in the plastids (*AT5G16390*, *AT5G35360*, *AT2G38040*, and *ATCG00500*), while the other two are homodimers, ACC1 (*AT1G36160*) and ACC2 (*AT1G36180*), located in the cytoplasm and chloroplasts [22]. In this study, homomeric ACCase was identified in Group I in both soybean and *Arabidopsis thaliana*, and all four subunits of the heteromeric ACCase could be identified within the same group in both species. Therefore, members of the same group had similar gene structures. In Group I, the identified gene family members primarily encoded homomeric ACCase and biotin carboxylase, in Group III, they primarily encoded biotin carboxyl carrier proteins, and in Groups II, IV, and V, they primarily encoded biotin carboxyltransferase. An important aspect of further structural exploration was to reveal the functions of the genes within each group and the functional differences among genes within the same group.

Previous studies have shown that, during the evolutionary process, gene families typically undergo tandem duplication or large-scale segmental duplication to maintain their gene integrity [51]. Segmental duplication events refer to the process in which a segment of a DNA sequence is repeated in the genome, usually distributed at distant locations, while homologous genes that are continuously repeated in the same genomic region are considered as tandem duplication events [52,53]. Soybean (*Glycine max*) is a typical paleopolyploid, whose ancestor diverged from the ancestor of green bean (*Phaseolus vulgaris*) and then experienced a whole-genome polyploidization event accompanied by a slow diploidization process, resulting in a whole-genome duplication rate of approximately 75% [44]. The analysis results indicated that the rates of segmental and tandem duplications in soybean (80%) were higher than the whole-genome duplication rate, suggesting that segmental duplication events and tandem duplication events play significant roles in the amplification of these *GmACCs*. We also found that the calculated Ka/Ks ratios for all gene pairs were less than one, indicating that these genes may experience strong purifying selection pressure during the evolutionary process. It has been reported that soybean is closely related to green bean and peanut during the evolutionary process of legumes [54]. Meanwhile, the experimental results showed that there were significantly more collinear gene pairs between soybean and peanut than between soybean and *Arabidopsis thaliana*. However, soybean did not exhibit collinear relationships with monocots like maize and rice, suggesting that this linear relationship may only exist among dicots.

ACCase catalyzes the conversion of Acetyl-CoA into Malonyl-CoA. There have been research reports where, in some soybean varieties, the total isoflavone content was negatively correlated with the oil content [55]. This study suggested that homomeric and heteromeric ACCases might play different roles in maintaining metabolic balance and responding to environmental changes. It has been reported that overexpressing the heteromeric *ACC* in *E. coli* and then introducing it into rapeseed results in an increased oil content in seeds [56]. The overexpression of α-CT from pea in *Arabidopsis thaliana* was found to enhance fatty acid synthesis [57,58]. The overexpression of the four subunits of the heteromeric ACCase can enhance lipid accumulation in cotton seeds [59]. In *Arabidopsis thaliana*, “BCCP-like” proteins act as negative regulators of ht*ACCase*. The silencing of this gene by RNA interference led to an increase in seed oil content [60]. In tobacco, it was discovered that the level of the accD subunit was a determinant of ACCase levels, and that enzyme levels were, in part, controlled post-transcriptionally at the level of subunit assembly [61]. However, once Acetyl-CoA is produced in the cytoplasm, the Malonyl-CoA produced by the homomeric ACCase enters various secondary metabolic pathways, participating in multiple plant responses [31]. It is inferred that the Malonyl-CoA produced under the catalysis of the heteromeric ACCase can promote fatty acid synthesis, while the homomeric ACCase may have a certain influence on the accumulation of isoflavones. Studies have found that the homomeric ACCase is primarily responsible for producing cytoplasmic Malonyl-CoA, which is used for flavonoid biosynthesis in *Arabidopsis thaliana* [33]. Since *Arabidopsis thaliana* lacks the key enzyme in the isoflavone biosynthesis process, isoflavone synthase, it cannot synthesize isoflavones [62]. However, isoflavone synthase is widely present in legumes and is capable of catalyzing the isoflavone biosynthesis reaction. Therefore, further verification of the functions of *GmACCs* will greatly enhance our understanding of the soybean isoflavone synthesis mechanism.

Gene expression patterns serve as vital clues for elucidating gene functions. In this study, we analyzed the expression profiles of 20 *GmACC* genes during seed development. The research indicated that most genes in Group I were highly expressed in both seeds and pods, genes in Group II, Group IV, and Group V were more highly expressed in pods, and most genes in Group III were more highly expressed in seeds. It is inferred that *BC* genes and homomeric *ACC* may play significant roles in the accumulation of specific substances during seed development, carboxyl transferase primarily functions in pods, and *BCCP* genes perform key functions during seed development. The accumulation levels of isoflavone-related compounds vary during seed development. Reports indicate that, after flowering for 25 days, the expression levels of genes related to daidzein and glycitein contents significantly change. Additionally, in the early stages of seed development, the expression levels of genes associated with genistein content also undergo significant changes [63,64]. To further validate the *GmACCs* associated with isoflavone accumulation, we conducted transcriptome sequencing of the seeds at the R5, R6, and R7 stages, followed by RT-qPCR verification. The results revealed that the expression patterns of most genes were consistent with the expression profiles, and their expression levels were lower during the R7 stage of soybean development. The R7 stage marks the beginning of maturity, when the seeds are about to enter a dormant state and the expression levels of most genes within the plant decrease. Studies have reported that isoflavones continuously accumulate during seed development, and genes related to isoflavone synthesis exhibit higher expression levels in the later stages of seed development [63]. It has been reported that genes involved in isoflavone synthesis, such as *CHS7*, *CHS8*, and *IFS2*, show a gradual increase in expression quantity throughout the seed development process, reaching their peak expression levels 60 days after flowering [65]. Therefore, this study selected four stages following the R5 period to analyze the correlation between the expression levels of the *GmACC* genes and isoflavone accumulation. Ultimately, five genes were found to have a significant positive correlation with isoflavone accumulation. Based on the literature and our experimental results, we preliminarily inferred that the expression of isoflavone-synthesis-related genes was most active during the R6 stage.

WGCNA analysis is an effective technique for classifying transcriptome data into co-expression modules, in order to reduce the number of potential candidate genes. Azam et al. [66] used a combination of GWAS and WGCNA to identify three modules highly correlated with total isoflavone content, ultimately determining that *Glyma.11G108100* affected the accumulation of isoflavone in soybean seeds. Azam et al. [67], based on BSA-seq and differentially expressed genes from WGCNA, identified four hub genes, of which the allele variation in *Glyma.06G290400* (*GmIE3-1*) significantly affected isoflavone accumulation. The isoflavone biosynthesis pathway involves multiple structural genes encoding enzymes, which interact through coordination or competition to promote isoflavone accumulation [68]. In this study, we constructed a weighted gene co-expression network using genes related to the isoflavone synthesis pathway and phenotypic data. Within the network, we found that the turquoise module was significantly positively correlated with the various components of isoflavones, showing an extremely significant positive correlation with GT. It has been reported that DZ and GT are key components affecting the total isoflavone content [2]. Therefore, identifying genes within the turquoise module could more accurately uncover the genes involved in regulating isoflavone accumulation.

By integrating the RT-qPCR and transcriptome sequencing results, we preliminarily identified *GmACC2*, *GmACC3*, and *GmACC17* as hub genes regulating isoflavone synthesis. *GmACC2* encodes homomeric ACCase. The homogeneous ACCase carboxylase plays a significant role in plant secondary metabolism. Studies have suggested that the homomeric ACCase in plants can catalyze the production of flavonoid metabolites [21]. Research has found that ACC missense mutants lack flavonoid accumulation during cold acclimation in leaves [69]. In *Arabidopsis thaliana*, under UV-B stress, the expression levels of cytosolic ACC and flavonoid-biosynthesis-related genes can significantly increase [70]. *GmACC3* was found to encode the biotin carboxyl carrier protein (BCCP), and *GmACC17* was found to encode the biotin carboxyl carrier protein subtype 2. Previous studies have shown that overexpressing *BCCP* increases fatty acid elongation and the production of flavonoids, terpenes, and phytosterols [32]. Both the antisense and sense expression of the biotin carboxyl carrier protein subtype 2 in *Arabidopsis thaliana* inactivated plastidial Acetyl-CoA carboxylase, reducing the seed oil content [71]. However, the role of this protein subtype in secondary metabolism remains unexplored. Therefore, it was speculated that *GmACC2* can directly participate in isoflavone biosynthesis, and that *GmACC3* and *GmACC17* can indirectly regulate the accumulation of isoflavones. In summary, this study employed a bioinformatics analysis and integrated transcriptomics with WGCNA to associate phenotypes, delving into the regulatory mechanism of isoflavone biosynthesis during soybean seed development. We found that the *GmACC2*, *GmACC3*, and *GmACC17* genes play significant roles in isoflavone biosynthesis. These results provide important insights into the molecular mechanisms underlying isoflavone biosynthesis in soybeans and offer theoretical support for the improvement of soybean varieties and the enhancement of isoflavone content.

## 4. Materials and Methods

### 4.1. Genome-Wide Identification of ACC Genes in Soybean

To identify the *ACC* gene family, we obtained the *Arabidopsis thaliana* ACCase protein sequences from TAIR (https://www.arabidopsis.org/) (accessed on 21 May 2024) and downloaded the complete set of soybean protein sequences from the Phytozome database [72]. Using these sequences as a reference, we searched the soybean protein database using the BLASTP program with an e-value threshold of 1 × 10^−5^. A local search based on a hidden Markov model (HMM) was conducted (http://hmmer.org/) (accessed on 22 May 2024) [73], using all known ACCase protein sequences from *Arabidopsis thaliana* to identify the *GmACC* genes in soybean. To more accurately identify the predicted *GmACC* genes from the genomic sequences, we analyzed the *GmACC*-like gene sequences based on the typical structural features of plant ACCase proteins reported previously in the NCBI online database (https://www.ncbi.nlm.nih.gov/) (accessed on 22 May 2024) [74]. As a result, we obtained 20 soybean *ACC* genes, which were renamed as *GmACC1*–*GmACC20*. (Table 1).

### 4.2. Prediction of Physicochemical Properties of GmACC Protein

We utilized Expasy-ProtParam (https://web.expasy.org/protparam/) (accessed on 23 May 2024) for the prediction of the protein physicochemical properties through pI/MW calculation [75]. Subcellular localization information on the soybean *GmACC* genes was obtained from the CELLO (http://cello.life.nctu.edu.tw/) (accessed on 24 May 2024) online web resource [76].

### 4.3. Construction of a Phylogenetic Tree

Based on the identified ACCase functional domains, we downloaded the ACCase protein sequences for peanut, rice, and maize from the Phytozome database [39]. Subsequently, we aligned the ACCase protein sequences from soybean, *Arabidopsis thaliana*, peanut, rice, and maize using the ClustalW algorithm in MEGA XI v11.0.13. All sequences were renamed according to their genetic characteristics as AtACC1-AtACC8, AhACC1-AhACC29, OsACC1-OsACC5, and ZmACC1-ZmACC7. A phylogenetic tree was constructed using the Maximum Parsimony method in MEGA XI, and bootstrap values were obtained based on 1000 replicates [77]. The tree was visualized using the online software Evolview v3 (https://www.evolgenius.info/evolview/#/treeview) (accessed on 24 May 2024) [78].

### 4.4. Gene Structure, Conserved Motif Analysis, and Promoter Cis-Regulatory Element Characterization of the GmACC Genes

We downloaded soybean genomic data, protein files, CDS files, and gff3 files from the Phytozome online website. We then used the online tool MEME motif analysis (https://meme-suite.org/meme/tools/meme) (accessed on 27 May 2024) to identify the conserved motifs in the soybean ACCase proteins [79]. In the MEME version 5.5.7 program, we set the number of conserved motifs to 15. Finally, we imported the generated files into TBtools (version 2.057) for visualization [40].

We extracted the 2000 bp sequence upstream of the start codon of the *GmACC* genes and submitted it to the PlantCare version 1 (https://bioinformatics.psb.ugent.be/webtools/plantcare/html/) (accessed on 27 May 2024) website for *cis*-acting element prediction [80]. After removing ambiguous elements and some basic elements, we identified 19 *cis*-elements. The results were visualized using the TBtools software.

### 4.5. Collinearity Analysis and Selective Pressure for Duplicated Genes

Tandem duplication events and segmental duplication events are considered to be the main drivers of gene family expansion within genomes [81]. Tandem duplications are defined as clusters of genes with sequence similarity and functional similarity that form within a 200 kb segment in the same chromosomal region [41]. The Simple Ka/Ks Calculator tool in TBtools software was used to estimate the synonymous substitution rate (Ks) and the non-synonymous substitution rate (Ka) to assess the impact sof selection pressure and divergence time on the *ACC* genes (parameter settings: CPU = 4) [45]. Subsequently, we analyzed tandem duplication events in the *GmACC* gene family using TBtools and MCScanX version git-97e74f40. Similarly, we investigated segmental duplication events and the collinearity relationships between gene pairs in different species using TBtools in conjunction with MCScanX and the BLASTP method.

### 4.6. Plant Materials and Gene Expression Analysis

The plant materials used in this study were grown in the potting field of Northeast Agricultural University in 2022 and included the low-isoflavone varieties L-79, Hong Feng No. 11, and Feng Shou No. 6, as well as the high-isoflavone varieties Sui 03-3952, Small-seeded Fodder Bean, and Zhong Dou 32 (Table S5). The plants were in good health with no disease or pest infestations. Using the SoyMD online platform (https://yanglab.hzau.edu.cn/SoyMD/#/) (accessed on 27 May 2024), we predicted the expression levels of the *GmACC* genes in various tissues during seed development [46]. Seeds at the beginning seed stage (R5), the full seed stage (R6), and the beginning maturity stage (R7) were selected for transcriptomic and RT-qPCR detection.

### 4.7. Total RNA Isolation and RT-qPCR Expression Analysis

To validate the expression profiles of the *GmACCs* in the multi-omics database SoyMD, we followed the methods described in the literature to extract the total RNA from the samples [82], and synthesized cDNA from mRNA using the ReverTra Ace^®^ qPCR RT Master Mix (TOYOBO, Life Science Department, Tokyo, Japan). The cDNA samples were diluted to 150 ng/μL with sterile double-distilled water and stored at −20 °C for later use. Three biological replicates for each sample were conducted for transcriptomic and RT-qPCR detection. We used actin as the internal reference gene [83], with primers designed using Primer Premier 5 (Table S8).

### 4.8. Transcriptome Sequencing Analysis and Correlation Analysis

To clarify the correlation between the expressions of the *GmACCs* and isoflavone accumulation, we selected seeds from the high-isoflavone variety Zhongdou 27 (4220.61 µg g^−1^) at the four following stages of grain development: 0 days, 7 days, 21 days, and 35 days after the R5 stage. Three biological replicates were conducted for each sample for transcriptomic analysis. Subsequently, the isoflavone content in the seeds was measured using high-performance liquid chromatography (HPLC), with the measurement method referenced from that of Wu et al. [84]. A network heatmap was generated using https://www.omicshare.com/ (accessed on 21 May 2024) [85].

Using Tbtools (version 2.057), we analyzed the mapping of clean reads and utilized them for HISAT2 version 2.2.1 [86]. Transcripts per million (TPM) values were used for gene/transcript-level quantification, and the genes obtained were selected based on the KEGG pathway [87]. We focused on genes involved in the isoflavone biosynthesis pathway, the flavonoid biosynthesis pathway, and the phenylpropanoid biosynthesis pathway as the key genes affecting isoflavone accumulation for further study.

### 4.9. Analysis of Weighted Gene Co-Expression Networks

Based on the definitions of high and low isoflavone content by Cai and Kim et al. [88,89], we chose varieties with a total isoflavone content in their seeds of less than 3000 µg g^−1^ as the low isoflavone varieties and those with a total isoflavone content greater than 3500 µg g^−1^ as the high isoflavone varieties (Table S5). Utilizing the contents of various isoflavone components in conjunction with transcriptomic data, a weighted gene co-expression network analysis was conducted using the WGCNA package in R software version R-4.2.2 [90]. We determined the soft thresholding power based on the scale-free network principle, selecting a soft threshold with a scale-free network fit index greater than 0.8 [91]. We identified co-expression patterns using dynamic tree cutting, constructed a gene clustering tree based on the correlation of gene expression levels, set the minimum number of genes per module to two, and then merged modules with similar expression patterns based on a module eigengene similarity threshold of 0.8 (Table S7).

## 5. Conclusions

In conclusion, this study systematically explored soybean *GmACC* genes across the entire genome by integrating bioinformatics analysis and WGCNA. It was determined that the *GmACC2*, *GmACC3*, and *GmACC17* genes play significant roles in the biosynthesis of isoflavones. These findings provide important evidence for further elucidating the molecular mechanisms of isoflavone biosynthesis in soybeans and offer theoretical support for the improvement of soybean varieties and the enhancement of isoflavone content.

## Figures and Tables

**Table 1:** The Acetyl-CoA carboxylase (*ACCase*) gene family in soybean.

Gene Name	Gene ID	Chromosome	Length (aa)	MW	pI	Subcellular Localization Prediction	Group
*GmACC1*	*Glyma.04G104900.*	4	2260	252,370.45	6.00	Cytoplasmic	I
*GmACC2*	*Glyma.06G105900*	6	2260	252,169.99	5.92	Cytoplasmic	I
*GmACC3*	*Glyma.13G363500*	13	298	32,437.37	8.49	Plasma Membrane, Chloroplast	III
*GmACC4*	*Glyma.15G010300*	15	269	29,161.57	8.81	Nuclear, Chloroplast, Mitochondrial	III
*GmACC5*	*Glyma.18G243500*	18	262	27,657.07	9.47	Chloroplast	III
*GmACC6*	*Glyma.18G265300*	18	284	29,764.27	8.45	Chloroplast	III
*GmACC7*	*Glyma.05G221100*	5	539	58,888.72	7.22	Mitochondrial	I
*GmACC8*	*Glyma.07G137400*	7	107	11,530.56	9.03	Mitochondrial	I
*GmACC9*	*Glyma.08G027600*	8	539	58,807.60	7.22	Mitochondrial	I
*GmACC10*	*Glyma.18G195700*	18	709	78,666.60	7.64	Mitochondrial, Cytoplasmic	V
*GmACC11*	*Glyma.18G195900*	18	690	76,918.92	8.63	Mitochondrial, Cytoplasmic	V
*GmACC12*	*Glyma.18G196000*	18	683	75,989.91	8.88	Cytoplasmic, Mitochondrial	V
*GmACC13*	*Glyma.10G208900*	10	34	3865.83	10.30	Mitochondrial, Chloroplast, Nuclear	IV
*GmACC14*	*Glyma.15G003800*	15	34	3847.76	10.00	Mitochondrial, Chloroplast	IV
*GmACC15*	*Glyma.15G248500*	15	126	14,897.90	4.51	Nuclear	II
*GmACC16*	*Glyma.09G248900*	9	261	27,536.94	9.37	Chloroplast	III
*GmACC17*	*Glyma.13G057400*	13	276	28,870.48	8.69	Chloroplast	III
*GmACC18*	*Glyma.11G233700*	11	297	32,064.75	9.01	Mitochondrial	III
*GmACC19*	*Glyma.18G023300*	18	291	31,540.09	9.10	Mitochondrial	III
*GmACC20*	*Glyma.19G028800*	19	280	29,326.88	8.16	Chloroplast	III

**Table 2:** The duplication events of *GmACCs* identified in soybean.

No.	Sequence	Duplication Type	Ka	Ks	Ka/Ks	Divergence Time (MYA)
1	*GmACC1* and *GmACC2*	Segmental	0.013	0.093	0.139	3.116
2	*GmACC3* and *GmACC4*	Segmental	0.034	0.064	0.528	2.134
3	*GmACC6* and *GmACC20*	Segmental	0.200	0.543	0.368	18.085
4	*GmACC7* and *GmACC9*	Segmental	0.008	0.124	0.066	4.141
5	*GmACC16* and *GmACC5*	Segmental	0.017	0.090	0.193	3.002
6	*GmACC17* and *GmACC6*	Segmental	0.183	0.552	0.331	18.416
7	*GmACC18* and *GmACC19*	Segmental	0.049	0.104	0.466	3.476
8	*GmACC17* and *GmACC20*	Segmental	0.046	0.077	0.601	2.569
9	*GmACC10* and *GmACC11*	Tandem	0.037	0.072	0.517	2.393
10	*GmACC11* and *GmACC12*	Tandem	0.014	0.054	0.259	1.798
11	*GmACC10* and *GmACC12*	Tandem	0.035	0.075	0.473	2.501

## Data Availability

The RNA_Seq data (ID: PRJNA1139955) is accessible: https://www.ncbi.nlm.nih.gov/bioproject/PRJNA1139955 (accessed on 2 June 2024). All figures and data are included in the manuscript and Supplementary Materials.

## Supplementary Materials

The supporting information can be downloaded at: https://www.mdpi.com/article/10.3390/ijms251810221/s1.
