# Supplementary material for: Bioinformatics Identification and Expression Analysis of Acetyl-CoA Carboxylase Reveal Its Role in Isoflavone Accumulation during Soybean Seed Development
Source: Int J Mol Sci. 2024 Sep 23;25(18):10221. doi: 10.3390/ijms251810221 (PMC11432495; doi:10.3390/ijms251810221)
Supplement: Supplementary file 1 [file ijms-25-10221-s001.zip › ijms-3171334-supplementary/File S1.Protein sequences of ACC genes.pdf]

Protein

>GmACC1

MADIGHRNGYVNSVLPNRPPAAISEVDDFCNALCGNRPIHSILIANNGMAAVKFIRSVRSW
AYETFGSEKAILLVAMATPEDMRINAEHIRIADQFVEVPGGTNNNNYANVQLILEMAEITH
VDAVWPGWGHASENPELPDALKAKGIVFLGPPAISMAALGDKIGSSLIAQAAEVPTLPWS
GSHVKIPPESSLITIPDEIYREACVYTTEEAVASCQVVGYPAMIKASWGGGGKGIRKVHND
DEVRALFKQVQGEVPGSPIFIMKVASQSRHLEVQLLCDQYGNVAALHSRDCSIQRRHQKII
EEGPITVAPIETVKQLEQAARRLAKSVNYVGAATVEYLFSMETGEYYFLELNPRLQVEHP
VTEWIAEINLPAAQVAIGMGIPLWQIPEIRRFYGVEHGGGYDAWRKTSVLATPFDFDKAQS
TRPKGHCVAVRVTSEDPDDGFKPTSGKVQELSFKSKPNVWAYFSVKSGGGIHEFSDSQFG
HVFAFGESRALAIANMVLGLKEIQIRGEIRTNVDYTIDLLNASDYRENKIHTGWLDSRIAM
RVRAERPPWYLSVVGGALYKASTSSAALVSDYVGYLEKGQIPPKHISLVHSQVSLNIEGSK
YTIDMIRGGSGSYRLRMNQSEIEAEIHTLRDGGLLMQLDGNSHVIYAEEEAAGTRLLIDGR
TCLLQNDHDPSKLVAETPCKLLRYLVADDSHVDADTPYAEVEVMKMCMPLLSPASGIIHF
KMSEGQAMQAGELIARLDLDDPSAVRKAEPFTGSFPVLGPPTAISGKVHQKCAASLNAAR
MILAGYEHNIDEVVQSLLNCLDSPELPFLQWQECLAVLATRLPKDLKNELESKYKEFEGIS
SSQIVDFPAKLLKGILEAHLSSCPDKEKGAQERLVEPLLSLVKSYEGGRESHAHIIVQSLFEE
YLSVEELFSDNIQADVIERLRLQYQKDLLKIVDIVLSHQGIKSKNKLILLLMDKLVYPNPAA
YRDQLIRFSLLNHTNYSELALKASQLLEQTKLSELRSNIARSLSELEMFTEDGENIDTPKRK
SAINDRMEDLVSAPLAVEDALVGLFDHSDHTLQRRVVETYIRRLYQPYLVKGSVRMQWH
RSGLIATWEFYDEYIERKNGVEDQTLNKMVEEKHGEKKWGVMVIIKSLQFLPAIISAALRE
ATNNLHEALTSGSVEPVNYGNMMHIGLVGINNQMSLLQDSGDEDQAQERINKLAKILKEH
EVGSTIRAAGVRVISCIIQRDEGRAPMRHSFHWSEEKLYYAEEPLLRHLEPPLSIYLELDKL
KAYENIRYTPSRDRQWHLYTVVDHKPQPIQRMFLRTLLRQPTTNEGFSSYQRLDAETSRTQ
LAMSFTTRSIFRSLMAAMEELELNAHNANIKSEHAHMYLYIIREQQIDDLVPYPKRINIDAG
KEETTVEAILEELAREIHSSVGVRMHRLGVVVWEVKLWMAACGQANGAWRVIVNNVTG
HTCTVHIYREKEDTVTHKVVYRSVSIKGPLHGVPVNENYQPLGVIDRKRLSARKNSTTYC
YDFPLAFETALEQSWAIQQPGFQRAKDKNLLKVTELKFADKEGSWGAPLVPVERYPGLND
VGMVAWFMEMCTPEFPSGRTILVVANDVTFKAGSFGPREDAFFRAVTDLACTKKLPLIYL
AANSGARLGVAEEVKSCFRVGWSEESNPEHGFQYVYLTPEDYARIGSSVIAHELKLESGET
RWVIDTIVGKEDGLGVENLSGSGAIAGAYSRAYKETFTLTYVTGRTVGIGAYLARLGMRCI
QRLDQPIILTGFSALNKLLGREVYSSHMQLGGPKIMATNGVVHLTVSDDLEGISSILKWLS
YIPSHVGGALPIVKPLDPPERPVEYFPENSCDPRAAISGTLDGNGRWLGGIFDKDSFVETLE
GWARTVVTGRAKLGGIPVGIVAVETQTVMQIIPADPGQLDSHERVVPQAGQVWFPDSATK
TAQAILDFNREELPLFILANWRGFSGGQRDLFEGILQAGSTIVENLRTYKQPIFVYIPMMGE
LRGGAWVVVDSRINSDHIEMYADRTAKGNVLEPEGMIEIKFRTRELLESMGRLDQQLITLK
VKLQEAKSNRDIAAFESLQQQIKSRERQLLPVYTQIATKFAELHDTSLRMAAKGVVREVL
DWCNSRAVFYQRLHRRIGEQSLINSVRDAAGDQLSHASALNLLKEWYLHSDIAKGRADA
WLDDKAFFRWKDNPANYENKLKELRAQKVLLQLTNIGDSALDLQALPQGLAALLSKLEP
SGRVKLTDELRKVLG

>GmACC2

MADIGRRNGYANSVLPNRPPAAISEVDEFCNALGGNRPIHSILIANNGMAAVKFIRSVRSW
AYETFGSEKAILLVAMATPEDMRINAEHIRIADQFVEVPGGTNNNNYANVQLILEMAEITH

VDAVWPGWGHASENPELPDALKAKGIVFLGPPAISMAALGDKIGSSLIAQAAEVPTLPWSGSH
VKIPPESSLITIPDEIYREACVYTTEEAVASCQVVGYPAMIKASWGGGGKGIRKVHND
DEVRALFKQVQGEVPGSPIFIMKVASQSRHLEVQLLCDQYGNVAALHSRDCSVQRRHQKI
IEEGPITVAPIETVKKLEQAARRLAISVNYVGAATVEYLYSMETGEYYFLELNPRLQVEHP
VTEWIAEINLPAAQVAIGMGVPLWQIPEIRRFYGVEHGGGYDAWRKTSVLATPFDFDKAQ
STRPKGHCVAVRVTSEDPDDGFKPTSGKVQELNFKSKPNVWAYFSVKSGGGIHEFSDSQF
GHVFAFGESRALAIANMVLGLKEIQIRGEIRTNVDYTIDLLNASDYRENKIHTGWLDSRIA
MRVRAERPAWYLSVVGGALYKASASSAALVSDYVGYLEKGQIPPKHISLVHSQVSLNIEG
SKYTIDMIRGGSGSYRLRMNQSEIEAEIHTLRDGGLLMQLDGNSHVIYAEEEAAGTRLLID
GRTCLLQNDHDPSKLVAETPCKLLRYLVADDSHVDADTPYAEVEVMKMCMPLLSPASGII
HFKMSEGQAMQAGELIARLDLDDPSAVRKAEPFTGSFPVLGPPTAISGKVHQKCAASLNA
ARMILSGYEHNIDEVVQSLLNCLDSPELPFLQWQECLAVLATRLPKELKNELESKYKEFEG
ISSSQIVDFPAKLLKGIIEAHLSSCPDKEKGAQERLVEPLLSLVKSYEGGRESHAHIIVQSLF
DEYLSVEELFSDNIQADVIERLRLQYKKDLLKIVDIVLSHQGIKSKNKLILQLMDKLVYPN
PVAYRDQLIRFSLLNHTNYSELALKASQLLEQTKLSELRSNIARSLSELEMFTEDGENIDTP
KRKSAINDRMEDLVSAPFAVEDALVGLFDHSDHTLQRRVVESYIRRLYQPYLVKGSARMQ
WHRSGLIATWEFYDEYIERKNGVEDQSLSKTVEEKHSEKKWGVMVIIKSLQFLPAIITAAL
REATNNPHEALTSGSVEPVNYGNMMHIGLVGINNQMSLLQDSGDEDQAQERINKLAKILK
EQEVGSTIRAAGVGVISCIIQRDEGRAPMRHSFHWSEEKLYYAEEPLLRHLEPPLSIYLELD
KLKAYENIRYTPSRDRQWHLYTVVDHKPQPIQRMFLRTLVRQPTTNEGFSSYQRLDAETSR
TQLAMSFTSRSIFRSLMAAMEELELNAHNVNIKSEHAHMYLYIIREQQIDDLVPYPKRINIE
AGKEEITVEAVLEELAREIHSSVGVRMHRLGVVVWEIKLWMAACGQANGAWRVIVNNV
TGHTCTVHLYREKEDTITHKVVYSSVSVKGPLHGVAVNENYQPLGVIDRKRLSARKNSTT
YCYDFPLAFETALEQSWAIQQPGFQRAKDKNLLKVTELKFADKEGSWGTPLVPVENYPGL
NDVGMVAWFMEMCTPEFPSGRTILVVANDVTFKAGSFGPREDAFFRAVTDLACTKKLPLI
YLAANSGARLGVAEEVKSCFRVGWSEESNPENGFQYVYLTPEDNARIGSSVIAHELKLES
GETRWVIDTIVGKEDGLGVENLSGSGAIAGAYSRAYKETFTLTYVTGRTVGIGAYLARLG
MRCIQRLDQPIILTGFSALNKLLGREVYSSHMQLGGPKIMATNGVVHLTVSDDLEGVSSIL
KWLSYIPSHVGGALPIVKPLDPPERPVEYFPENSCDPRAAISGTLDGNGRWLGGIFDKDSF
VETLEGWARTVVTGRAKLGGIPVGVVAVETQTVMQIIPADPGQLDSHERVVPQAGQVWFP
DSATKTAQAILDFNREELPLFILANWRGFSGGQRDLFEGILQAGSTIVENLRTYKQPIFVYIP
MMGELRGGAWVVVDSRINSDHIEMYADRTAKGNVLEPEGMIEIKFRTRELLESMGRLDQ
QLITLKAKLQEAKSSRNIVAFESLQQQIKSRERQLLPVYTQIATKFAELHDTSLRMAAKGVI
REVLDWRNSRSVFYQRLHRRIGEQSLINSVRDAAGDQLSHASAMNLLKEWYLNSDIAKG
REDAWLDDEAFFRWKDIPSNYENKLKELRVQKVLLQLTNIGDSALDLQALPQGLAALLSK
LEPLGRVKLTDELRKVLG
>GmACC3
MLFTFFTSLPFTLLCDTHSFCFTFSMASCSIGTPNIKVLNLHFGGKKVGLSRQFGTRSWISR
LQYTSLVMSRQTVRFLASSNGPSTEIQFAARSEGSEEIRSSGLTSELIPNINEVEFLLTKLCDT
SSIGELDLKLAGFHLHVVRDLTEKTKTLPPLIPASVSIINVTETPKTNGSVPTTSLAVSKPVD
PVPSSGSIQRFLDKAADEGLVIIQSPKVGFFRRSRTIKGKRAPPSCKEKQNVEEGQVICYIEQ
LGGELPIESDVSGEVIKILRQDGDPVGYGDALVAILPSFPGIKKLQ
>GmACC4
MASCSIGTPNIKALNLHFGGKKVGLSQQFGTRSWISKQSLQYTSLVMSRQKVRFSPTEIQF

VTRSEGSEEVKSSGLTSELIPNLIEVEFLLTKLCDTSSIGELDLKLAGFHLHVVRDLTEKTKTLPP
PIPASESIINVTETPKTNGSVSTTSLAVSKPVDPIPSSGSIQRFLNKAADEGLVIIQSPKVG
FFRRSRTIKGRRAPPSCKEKQNVEEGQVICYIEQLGGELPIESDVSGEVIKILQKDGDPVGY
GDALVAILPSFPGIKKLQ
>GmACC5
MASSLAPATKAATNLRLTHSLRFSPKPNNLRFATKPGNTLLCTRVKAQLNEVALDSSSNATS
PPMKAKSKEEPPAKPLAEPSSSVLATQESVSQFITQVASLVKLVDSRDIVELKLKQHDVEVT
IRKKEAMPQPPPAPQPSVVYSPPPPALPPPPVPASTPAPTLARATPTPTSAPAVKSAKSSLPPL
KSPMAGTFYRSPAPGEPSFVKVGDKVKKGQVVCIIEAMKLMNEIEADQSGTIVEIVAEDA
KSVSVDTPLFVIQP
>GmACC6
MASFTIPCPKCVVVPFAHLGLNSQTQQRNALGLKKSLSFGSLSSDSAPNGIQCLNKKQSSV
WKLQAQPKEAVTVENSAPVQVNGPKIAPPEEKDDHNGKPSGPSTSADASSISAFMNQVSD
LVKLVDSKDIMELQLKQANCELVIRKKEALLPPPATFVAPVSQPFPYPTNSLPAAPPPVATST
PASSPSSKAAPALPPAKASKSSHPALKCPMAGTFYRSPAPGEPPFVKVGDKVQKGQVICIIE
AMKLMNEIEADQSGTVAEVVAEDGKPVSVDTPLFVIVP
>GmACC7
MEVTLSACKSISSPSVPVAALFAGKAGTKSSQCSFLAGANKVRFPRQVGQVSHVRKQRQT
RHCGALHATCSGDKILIANRGEIAVRVIRTAHELGIPCVAVYSTIDKDALHVKLADEAVCIG
EAPSSQSYLLIPNVLSAAISRRCTMLHPGYGFLAENAVFVEMCREHGINFIGPNPDSIRVMG
DKATARETMKKAGVPTVPGSDGLLQSTEEAIRLANEIGFPVMIKATAGGGGRGMRLAKEP
DEFVKLLQQAKSEAAAAFGNDGVYLEKYIQNPRHIEFQVLADKYGNVVHFGERDCSIQR
RNQKLLEEAPSPALTPELRKAMGDAAVAAAASIGYIGVGTIEFLLDERGSFYFMEMNTRIQ
VEHPVTEMISSTDLIEEQIRVAMGEKLRYKQEDIVLRGHSIECRINAEDAFKGFRPGPGRITA
YLPSGGPFVRMDSHVYPDYVVPPSYDSLLGKLIVWAPTREKAIERMKRALDDTIITGVPTT
IDYHKLILDIEDFRNGKVDTAFIPKHEEELAMPPQKMVLANRVNELAGSTA
>GmACC8
MKMCMPLLSPASGIIHFKMSEGQAMQAGELIARLHLDDPSTVRKAEPFTGSFPVLGPPTAI
SGKVHQKCAASLNAARMILSGYDHNIDEGIKSKNKLILQLMDKLGN
>GmACC9
MEVTLSACKSVSSPSVPVAGLFAGNGGIKSSQCSFLAGASKVRFPRQVGQVSHLRKQRQT
RHCGALHATCSGDKILIANRGEIAVRVIRTAHELGIPCVAVYSTIDKDALHVKLADEAVCIG
EAPSSQSYLLIPNVLSAAISRRCTMLHPGYGFLAENAVFVEMCREHGINFIGPNPDSIRVMG
DKATARETMKKAGVPTVPGSDGLLQSTEEAIRLANEIGFPVMIKATAGGGGRGMRLAKEP
AEFVKFLQQAKSEAAAAFGNDGVYLEKYIQNPRHIEFQVLADKYGNVVHFGERDCSIQR
RNQKLLEEAPSPALTPELRKAMGDAAVAAAASIGYIGVGTVEFLLDERGSFYFMEMNTRI
QVEHPVTEMISSTDLIEEQIRVAMGEKLRYKQEDIVLRGHSIECRINAEDAFKGFRPGPGRIT
AYLPSGGPFVRMDSHVYPDYVVPPSYDSLLGKLIVWAPTREKAIERMKRALDDTIITGVPT
TIDYHKLILDIEDFRNGKVDTAFIPKHEEELAMPPQKMVLANRVNELAGSTA
>GmACC10
MAASSASLSGASASDLLRSSTSGFNGVPLRTMGKGKLVLKRRNFTVAARLRKVKKHEYP
WPPNPDPNVKGGVLSHLSLFKPLKEKPKPVTLDFERPLVDLQKKIIDVQKMANETGLDFS
DQILSLETKYHQALKDLYTHLTPIQRVNIARHPNRPTFLDHVFNITEKFVELHGDRAGYDD
PAIVTGLGTIDGRSYMFIGHQKGRNTKENIQRNFGMPTPHGYRKALRLMEYADHHGFPIV

TFIDTPGAYADLKSEELGQGEAIAHNLRSMFGLKVPVISIVIGEGGSGGALAIGCANKLLMLEN
AVFYVASPEACAAILWKTAKASPKAAEKLKITATELCKLQIADGVIPEPLGGAHADPE
WTSQQIKKAIKETMDELMKMNTEELLKHRMLKFRKIGGFQEGIPIDPKRKANMKKRDLSI
AKIPDAELEVEVEKLKQQVLEAKESSPVPPKLDLDEMLKQLAREVDLEYSEAVKATGLTD
SLLKLREEVSKANADNQIVDPLLEGKIEKLRVEFEQQLRAAPNYGRLQNKLNYLSELCKV
KLLSDGKKDNEAVTFKQELKKKIDNALSDPKIRETFEALKAEIKGVGASSASDLDDELKK
KIIEFIKEVKEVKEVKEVIENQIESLVNSSDDIKSKILQLKLEVPKAGETPDSEPKNRIGALV
QLIKPSLVEAVDSSGLKDLFENLVSNDGSLTHEDPARDSLTDDQ
>GmACC11
MAASSASLSGASASDLLRSSTSGFNGVPLRTLGKGKLVLKRRDFTVAAKLRKVKKHEYP
WPPNPDPNVKGGVLSHLSMFKPLKEKPKPVTLDFEKPLVDLQKKIIDVQKMANETGLDFS
DQILSLETKYQQALKDLYTHLTPIQRVNIARHPNRPTFLDHVFNITEKFVELHGDRAGYDD
PAIVTGLGTIDGRSYMFIGHQKGRNTKENIQRNFGMPTPHGYRKALRLMEYADHHGFPIV
TFIDTPGAYADLKSEELGQGEAIAHNLRSMFGLKVPVISIVIGEGGSGGALAIGCANKLLM
LENAVFYVASPEACAAILWKTAKASPKAAEKLKITATELCKLQIADGVIPEPLGGAHADPE
WTSQQIKKAIKETMDELMKMNTEELLKHRMLKFRKIGGFQEGIPIDPKRKANMKKRDLSI
AKISDAELEVEVEKLKQQVLEAKESSPVPPKLDLDEMLKQLTREVDLEYSEAVKATGLTD
SLLKLREEVSKANADNQIVDPLLKDKIEKLRVEFEQQLRAAPNYGRLQNKFTYLSELCKV
KLLSDANKDNEAVTFKQELEKKVDNALSNPKIRETFEALKAEIKGAGASSASDLDDELKK
KIVEFMIELKEVKEVKEVIENQIESLVNSSDDIKNKVLQLKLEVPKAGETPDSESKSRIGDFI
FRTSSRIIMAILLRDMFGNFKEIPC
>GmACC12
MAASSASLSGASASDLLRSSTSGFNGVPLRTLGKGKLVLKRRDFTVAAKLRKVKKHEYP
WPANPDPNVKGGVLSHLSLFKPLKEKPKPVTLDFEKPLVDLQKKIIDVQKMANETGLDFS
DQILSLENKYQQALKDLYTHLTPIQRVNIARHPNRPTFLDHVFNITEKFVELHGDRAGYDD
PAIVTGLGTIDGRSYMFIGHQKGRNTKENIQRNFGMPTPHGYRKALRLMEYADHHGFPIV
TFIDTPGAYADLKSEELGQGEAIAHNLRSMFGLKVPVISIVIGEGGSGGALAIGCANKLLM
LENAVFYVASPEACAAILWKTAKASPKAAEKLKITATELCKLQIADGVIPEPLGGAHADPE
WTSQQIKKAIKETMDELTKMNTEELLKHRMLKFRKIGGFQEGIPIDPKRKANMKKRDLSI
AKIPDAELEVEVEKLKQQVLEAKESSPVPPKLDLDEMLKQLAREVGLEYSEAVKATGLTD
SLLKLREEVSKANADSQIVDPLLKDKIEKLRVEFEQQLRAAPNYGRLQNKFKYLSELCKV
KLLSDANKDNQAVTFKQELEKKVDNALSDPKIRETFEALKAEIKGAGASSASDLDDELKK
KIVGFMIELKEVKEVKEVIENQIESLVNSSDDIKSKILQLKLELPKAGETPDSESNNRIGDFIF
KKSSKIIMAILLRDMFW
>GmACC13
MINFPSIFVPLVGLVFPAIAMVSLFFHVKKNKIF
>GmACC14
MINFPSIFVPLVGLIFPAIAMTSLFLHVQKNKIF
>GmACC15
MLFNRKLEYRCELSKSMDSLGPIENTSLREDPKILTDIEKKIHKDLDYLEMEGFFSSDLNTV
SKNDDDHYMYETQFSFNNNITSFIDSCIESFNLGDIDKYNDIYFYSYIFLRAEIVVKVIILVL
V
>GmACC16
MASSLAPATKAATNLRLTHSLRFSPKPNNLRFATKPGNTLLCARVKAQLDEVALDSSSNAT

PPIKAKSKEEPPAKPLAEPSSSVLATQESVSQFITQVASLVKLVDSRDIVELKLKQHDVEVTIRK
KEAMPQPPPPPQPAVVYSSPPPVLPPPSVPASTPAPTLARATPSPTSAPAVKSAKSSLPPL
KSPMAGTFYRSPAPGEPPFVKVGDKVKKGQVVCIIEAMKLMNEIEADQSGTIVEIVAEDA
KSVSVDTPLFVIQP
>GmACC17
MASFSVPCPKCPTTSSSPPLGLKSLNVSFQRVLSLKPSLSFGSLSAESAASRIQCLNRKQFSV
LKATKVENSNSAPVMVNGPNVASSKEKEVHNGKLSGGTIPDDASIIAFMSQVSDLVKLVD
SRDIVELQLKQSDCELMIRKKEALQPPPVIAPTSPPMHYATVPSPPPPPAAAPASSAPPKAVP
ALPSPAKAGTSSHPTLKCPMAGTFYRSPAPGEPAFVKVGDKVQKGQVICIIEAMKLMNEIE
ADQSGTIAEVLAEDGKPVSVDTPLFVIVP
>GmACC18
MFLQIYNQRMLCNHPRAYPIGTMSHVRASLEKQAVVPIHNAGWNSKSRLFIQHLAYGQK
HINSHTKGKNTLISCGKTAEAINASKSDASSDNTPQGSLEKKPLQTATFPNGFEALVLEVCD
ETEIAELKVKVGDFEMHIKRNIGATKVPLSNISPTTPPPIPSKPMDESAPGSLPPSPPKSSPEK
NNPFANVSKEKSPRLAALEASGTNTYVLVSSPTVGLFRRGRTVKGKKQPPICKEGDVIKEG
QVIGYLDQFGTGLPIKSDVAGEVLKLLVEDGEPVGYGDPLIAVLPSFHDIK
>GmACC19
MESSPAIRSFHYPMGTMSHVRACLEKQAVLPIHNARWNSKRRLFIQHLAYGQKHINSHMK
GKSTLVSSAKTAEAINTSNSDASSDNTPQGSLEKKPLQTATFPNGFEALVLEVCDETEIAEL
KVKVGDFEMHIKRNIGATKVPLSNISPTTPPPIPSKPMDESAPNSLPPSPPKSSPEKNNPFAN
VSKEKSPKLAALEASGTNTYVLVTSPTVGLFRRGRTVKGKKQPPICKEGDVIKEGQVIGYL
DQFGTGLPIRSDVAGEVLKLLVEDGEPVGYGDRLIAVLPSFHDIK
>GmACC20
MASFSVPCPKCPTTSSSSSLPLGLNSQKVSFQSGLILKPSLSFGSLSAESAASRIQCLNRKQF
SVLKATKVENSNSAPVTVNGPTVASSKENQVHNGKLSDTTIPDEASIIAFMSQVSDLVKLV
DSRDIVELQLKQSDCELMIRKKEALQPPPIIAPAPPPMHYATFPSPSSPLPAEAAPASSAPPK
AAPALPSPGKASTSSHPPLKCPMAGTFYRSPAPGEPAFVKVGDKVKKGQVICIIEAMKLMN
EIEADQSGTIAEVLAEDGKPVSVDMPLFVIVP
>AtACC1
MAGSVNGNHSAVGPGINYETVSQVDEFCKALRGKRPIHSILIANNGMAAVKFIRSVRTWA
YETFGTEKAILLVGMATPEDMRINAEHIRIADQFVEVPGGTNNNNYANVQLIVEMAEVTR
VDAVWPGWGHASENPELPDALDAKGIIFLGPPASSMAALGDKIGSSLIAQAADVPTLPWS
GSHVKIPPNSNLVTIPEEIYRQACVYTTEEAIASCQVVGYPAMIKASWGGGGKGIRKVHND
DEVRALFKQVQGEVPGSPIFIMKVASQSRHLEVQLLCDKHGNVSALHSRDCSVQRRHQKI
IEEGPITVAPPETVKKLEQAARRLAKSVNYVGAATVEYLYSMDTGEYYFLELNPRLQVEH
PVTEWIAEINLPAAQVAVGMGIPLWQIPEIRRFYGIEHGGGYDSWRKTSVVAFPFDFDKAQ
SIRPKGHCVAVRVTSEDPDDGFKPTSGRVQELSFKSKPNVWAYFSVKSGGGIHEFSDSQFG
HVFAFGESRALAIANMVLGLKEIQIRGEIRTNVDYTIDLLHASDYRDNKIHTGWLDSRIAM
RVRAERPPWYLSVVGGALYKASATSAAVVSDYVGYLEKGQIPPKHISLVHSQVSLNIEGSK
YTIDVVRGGSGTYRLRMNKSEVVAEIHTLRDGGLLMQLDGKSHVIYAEEEAAGTRLLIDG
RTCLLQNDHDPSKLMAETPCKLMRYLISDNSNIDADTPYAEVEVMKMCMPLLSPASGVIH
FKMSEGQAMQAGELIANLDLDDPSAVRKAEPFHGSFPRLGLPTAISGRVHQRCAATLNAA
RMILAGYEHKVDEVVQDLLNCLDSPELPFLQWQECFAVLATRLPKNLRNMLESKYREFES
ISRNSLTTDFPAKLLKGILEAHLSSCDEKERGALERLIEPLMSLAKSYEGGRESHARVIVHS

LFEEYLSVEELFNDNMLADVIERMRQLYKKDLLKIVDIVLSHQGIKNKNKLVLRLMEQLVYPN
PAAYRDKLIRFSTLNHTNYSELALKASQLLEQTKLSELRSNIARSLSELEMFTEDGEN
MDTPKRKSAINERIEDLVSASLAVEDALVGLFDHSDHTLQRRVVETYIRRLYQPYVVKDSV
RMQWHRSGLLASWEFLEEHMERKNIGLDDPDTSEKGLVEKRSKRKWGAMVIIKSLQFLP
SIISAALRETKHNDYETAGAPLSGNMMHIAIVGINNQMSLLQDSGDEDQAQERVNKLAKI
LKEEEVSSSLCSAGVGVISCIIQRDEGRTPMRHSFHWSLEKQYYVEEPLLRHLEPPLSIYLE
LDKLKGYSNIQYTPSRDRQWHLYTVTDKPVPIKRMFLRSLVRQATMNDGFILQQGQDKQ
LSQTLISMAFTSKCVLRSLMDAMEELELNAHNAAMKPDHAHMFLCILREQQIDDLVPFPR
RVEVNAEDEETTVEMILEEAAREIHRSVGVRMHRLGVCEWEVRLWLVSSGLACGAWRVV
VANVTGRTCTVHIYREVETPGRNSLIYHSITKKGPLHETPISDQYKPLGYLDRQRLAARRS
NTTYCYDFPLAFGTALELLWASQHPGVKKPYKDTLINVKELVFSKPEGSSGTSLDLVERPP
GLNDFGMVAWCLDMSTPEFPMGRKLLVIANDVTFKAGSFGPREDAFFLAVTELACAKKL
PLIYLAANSGARLGVAEEVKACFKVGWSDEISPENGFQYIYLSPEDHERIGSSVIAHEVKLS
SGETRWVIDTIVGKEDGIGVENLTGSGAIAGAYSKAYNETFTLTFVSGRTVGIGAYLARLG
MRCIQRLDQPIILTGFSTLNKLLGREVYSSHMQLGGPKIMGTNGVVHLTVSDDLEGVSAIL
NWLSYIPAYVGGPLPVLAPLDPPERIVEYVPENSCDPRAAIAGVKDNTGKWLGGIFDKNSF
IETLEGWARTVVTGRAKLGGIPVGVVAVETQTVMQIIPADPGQLDSHERVVPQAGQVWFP
DSAAKTAQALMDFNREELPLFILANWRGFSGGQRDLFEGILQAGSTIVENLRTYRQPVFV
YIPMMGELRGGAWVVVDSQINSDYVEMYADETARGNVLEPEGTIEIKFRTKELLECMGRL
DQKLISLKAKLQDAKQSEAYANIELLQQQIKAREKQLLPVYIQIATKFAELHDTSMRMAA
KGVIKSVVEWSGSRSFFYKKLNRRIAESSLVKNVREASGDNLAYKSSMRLIQDWFCNSDI
AKGKEEAWTDDQVFFTWKDNVSNYELKLSELRAQKLLNQLAEIGNSSDLQALPQGLANL
LNKVEPSKREELVAAIRKVLG

>AtACC2

MEMRALGSSCSTGNGGSAPITLTNISPWITTVFPSTVKLRSSLRTFKGVSSRVRTFKGVSST
RVLSRTKQQFPLFCFLNPDPISFLENDVSEAERTVVLPDGSVNGAGSVNGYHSDVVPGRNV
AEVNEFCKALGGKRPIHSILVATNGMAAVKFIRSVRTWAYETFGSEKAVKLVAMATPEDMR
INAEHIRIADQFVEVPGGTNNNNYANVQLIVEMAEVTRVDAVWPGWGHASENPELPDAL
KEKGIIFLGPPADSMIALGDKIGSSLIAQAADVPTLPWSGSHVKIPPGRSLVTVPEEIYKKAC
VYTTEEAIASCQVVGYPAMIKASWGGGGKGIRKVHNDDEVRALFKQVQGEVPGSPIFIM
KVASQSRHLEAQLLCDQYGNVAALHSRDCSVQRRHQKIIEEGPITVAPQETIKKLEQAARR
LAKSVNYVGAATVEYLYSMDTGEYYFLELNPRLQVEHPVTEWIAEVNLPAAQVAVGMGI
PLWQIPEIRRFYGMEHGGGYDSWRKTSVVASPFDFDEAESLRPKGHCVAVRVTSEDPDDG
FKPTSGEIQELSFKSKPNMWSYFSVKSGGGIHEFSDSQFGHVFAFGESRSVAIANMVLALK
EIQIRGDIRTNVDYTIDLLHASDYRENKIHTGWLDSRIAMRVRAERPPWYLSVVGGALYK
ASTTSSAVVSDYVGYLEKGQIPPKHISLVHSQVSLNIEGSKYTIDVVRGGSGTYRLRMSNS
EVVAEIHTLRDGGLLMQLDGKSHVIYAKEEATGTRLLIDGRTCLLQNDHDPSKLMAETPC
KLLRYLVSDNSSIDTDTPYAEVEVMKMCMPLISPASGVIHFKLSEGQAMQAGELIAKLDLD
DPSAVRKAKPFRGSFPRLGLPTAISGKVHQRCAATLNAARMILAGYDHKVDEVLQDLLNC
LDSPELPFLQWQECFAVLATRLPKDLRNMLELKYKEFEIISKTSLTPDFPAKLLKGILEAHLS
SCDEKERGSLERLIEPLMSLVKSYEGGRESHARLIVHSLFEEYLSVEELFNDNMLADVIER
MRQQYKKDRLKIVDIVLSHQGIIHKNKLVLRLMEQLVYPNPAAYREKLIRFSALNHTNYS
QLALKASQLLEQTKRSELRSNIARSLSELEMFTEAGENMDTPKRKSAISETMENLVSSSLA
VEDALVGLFDHSDHTLQRRVVETYIHRLYQPYVVKESVRMQWHQSGVIASWEFLEHFER

KNTGPDDHEISEKGIVAKSSKRKRGTMVIIKSLQFLPSIINASLRETNHSHCEYARAPLSGNMM
HIAVVGINNQMSLLQDSGDEDQTQERVNKLAKILKEEEVSLTLCSAGVGVISCIIQRDE
GRTPMRHSFHWLMEKQYYVEEPLLRHVEPPLSVYLELDKLKGYSNIQYSPSRDRQWHM
YSVTDRPVPIKRMFLRSLVRQTTMNDGFLLQQGQDYQLSQTVLSMAFTSKCILRSLMNA
MEELELNAHNAAMKPDHAHMFLCILREQQIDDLVPYPRRFEVNAEDEETTVETILEEATQ
EIHRSVGVRMHALGVCEWEVRLWLVSSGLANGAWRVVVANVTGRTCTVHIYREVEATG
RNSLIYHSITKKGPLHGTLINGQYKPLNNLDRKRLAARRSNTTYCYDFPLAFETALELNWA
SQHSGVRKPCKNRLINVKELVFSNTEGSLGTSLIPVERPAGLNDIGMVAWILEMSTPEFPM
GRKLLIVANDVTFKAGSFGPREDAFFLAVTELACTKKLPLIYLAANSGARLGVAEEVKACF
KVGWSDEVSPGNDFQYIYLSSEDYARIGSSVIAHEVKLPSGETRWVIDTIVGKEDGLGVEN
LTGSGAIAGAYSRAYNETFTLTFVSGRSVGIGAYLARLGMRCIQRLDQPIILTGFSTLNKLL
GREVYSSHMQLGGPKIMGTNGVVHLTVSDDLEGVSAILNWLSYIPAYVGGPLPVLAPLDP
PERTVEYIPENSCDPRAAIAGINDNTGKWLGGIFDKNSFVETLEGWARTVVTGRAKLGGIP
IGVVAVETQTVMHVIPADPGQLDSHERVVPQAGQVWFPDSAAKTAQALMDFNREQLPLFI
IANWRGFSGGQRDLFEGILQAGSAIVENLRTYRQPVFVYIPMMGELRGGAWVVVDSQINS
DYIEMYADETARGNVLEPEGMIEIKFRRKELLECMGRLDQTLINLKANIQDAKRNKAYANI
ELLQKQIKTREKQLLPVYTQIATKFAELHDTSMRMAAKGVIKSVVEWSGSRSFFYKKLYR
RIAESSLVRNIRKASGDILSYKSAMGLIQDWFRKSEIAKGKEEAWTDDQLFFTWKDNVSN
YEQKLSELRTQKLLNQLAEIGNSSDLQALPQGLANLLNKVDLSRREELVDAIRKVLG
>AtACC3
MASSSFSVTSPAAAASVYAVTQTSSHFPIQNRSRRVSFRLSAKPKLRFLSKPSRSSYPVVKA
QSNKVSTGASSNAAKVDGPSSAEGKEKNSLKESSASSPELATEESISEFLTQVTTLVKLVDS
RDIVELQLKQLDCELVIRKKEALPQPQAPASYVMMQQPNQPSYAQQMAPPAAPAAAAPAP
STPASLPPPSPPTPAKSSLPTVKSPMAGTFYRSPAPGEPPFIKVGDKVQKGQVLCIVEAMKL
MNEIESDHTGTVVDIVAEDGKPVSLDTPLFVVQP
>AtACC4
MASLSVPCVKICALNRRVGSLPGISTQRWQPQPNGISFPSDVSQNHSAFWRLRATTNEVVS
NSTPMTNGGYMNGKAKTNVPEPAELSEFMAKVSGLLKLVDSKDIVELELKQLDCEIVIRK
KEALQQAVPPAPVYHSMPPVMADFSMPPAQPVALPPSPTPTSTPATAKPTSAPSSSHPPLKS
PMAGTFYRSPGPGEPPFVKVGDKVQKGQIVCIIEAMKLMNEIEAEKSGTIMELLAEDGKP
VSVDTPLFVIAP
>AtACC5
MDASMITNSKSITSPPSLALGKSGGGGVIRSSLCNLMMPSKVNFPRQRTQTLKVSQKKLK
RATSGGLGVTCSGGDKILVANRGEIAVRVIRTAHEMGIPCVAVYSTIDKDALHVKLADEAV
CIGEAPSNQSYLVIPNVLSAAISRGCTMLHPGYGFLSENALFVEMCRDHGINFIGPNPDSIR
VMGDKATARETMKNAGVPTVPGSDGLLQSTEEAVRVANEIGFPVMIKATAGGGGRGMRL
AKEPGEFVKLLQQAKSEAAAAFGNDGCYLEKFVQNPRHIEFQVLADKFGNVVHFGERDC
SIQRRNQKLLEEAPSPALTAELRKAMGDAAVAAAASIGYIGVGTVEFLLDERGSFYFMEM
NTRIQVEHPVTEMIYSVDLIEEQIRVAMGEKLRYKQEDIVLRGHSIECRINAEDPFKGFRPG
PGRITSYLPSGGPFVRMDSHVYSDYVVPPSYDSLLGKLIVWAPTREKAIERMKRALNDTII
TGVPTTINYHKLILDVEDFKNGKVDTAFIVKHEEELAEWDMKPLVKLNIEIGELHEKNETI
YRGRQIKLCL
>AtACC6
MASISHSSLALGGASSASASDYLRSSSNGVNGVPLKTLGRAVFTTIRRKDLAVTSRLKKGK

KFEHPWPANPDPNVKGGVLSYLAEFKPLGDTQKPVTLDFEKPLVELEKKIVDVRKMANETGL
DFTEQIITLENKYRQALKDLYTHLTPIQRVNIARHPNRPTFLDHIHNITDKFMELHGDR
AGYDDPAIVTGIGTIDGKRYMFIGHQKGRNTKENIMRNFGMPTPHGYRKALRMMYYADH
HGFPIVTFIDTPGAYADLKSEELGQGEAIANNLRTMFGLKVPILSIVIGEGGSGGALAIGCA
NKMLMLENAVFYVASPEACAAILWKTSKAAPEAAEKLRITSKELVKLNVADGIIPEPLGGA
HADPSWTSQQIKIAINENMNEFGKMSGEELLKHRMAKYRKIGVFIEGEPIEPSRKINMKKR
EAVFSDSRKLQGEVDKLKEQILKAKETSTEAEPSSEVLNEMIEKLKSEIDDEYTEAAIAVGL
EERLTAMREEFSKASSEEHLMHPVLIEKIEKLKEEFNTRLTDAPNYESLKSKLNMLRDFSR
AKAASEATSLKKEINKRFQEAVDRPEIREKVEAIKAEVASSGASSFDELPDALKEKVLKTK
GEVEAEMAGVLKSMGLELDAVKQNQKDTAEQIYAANENLQEKLEKLNQEITSKIEEVVRT
PEIKSMVELLKVETAKASKTPGVTEAYQKIEALEQQIKQKIAEALNTSGLQEKQDELEKEL
AAARELAAEESDGSVKEDDDDDEDSSESGKSEMVNPSFA
>AtACC7
MEKSWFNFMFSKGELEYRGELSKAMDSFAPGEKTTISQDRFIYDMDKNFYGWDERSSYS
SSYSNNVDLLVSSKDIRNFISDDTFFVRDSNKNSYSIFFDKKKKIFEIDNDFSDLEKFFYSYC
SSSYLNNRSKGDNDLHYDPYIKDTKYNCTNHINSCIDSYFRSYICIDNNFLIDSNNFNESYI
YNFICSESGKIRESKNYKIRTNRNRSNLISSKDFDITQNYNQLWIQCDNCYGLMYKKVKM
NVCEQCGHYLKMSSSERIELSIDPGTWNPMDEDMVSADPIKFHSKEEPYKNRIDSAQKTT
GLTDAVQTGTGQLNGIPVALGVMDFRFMGGSMGSVVGEKITRLIEYATNQCLPLILVCSSG
GARMQEGSLSLMQMAKISSVLCDYQSSKKLFYISILTSPTTGGVTASFGMLGDIIIAEPYAYI
AFAGKRVIEQTLKKAVPEGSQAAESLLRKGLLDAIVPRNLLKGVLSELFQLHAFFPLNTN
>AtACC7
MASSSFSVTSPAAAASVYAVTQTSSHFPIQNRSRRVSFRLSAKPKLRFLSKPSRSSYPVVKA
QSNKVSTGASSNAAKVDGPSSAEGKEKNSLKESSASSPELATEESISEFLTQVTTLVKLVDS
RDIVELQLKQLDCELVIRKKEALPQPQAPASYVMMQQPNQPSYAQQMAPPAAPAAAAPAP
STPASLPPPSPPTPAKSSLPTVKSPMAGTFYRSPAPGEPPFIKVGDKVQKGQVLCIVEAMKL
MNEIEVSF*
>OsACC1
MEGSYQMNGILNGMSNSRHPSSPSEVDEFCKALGGDSPIHSVLVANNGMAAVKFMRSIRT
WALETFGTEKAILLVAMATPEDLKINAEHIRIADQFVEVPGGTNNNNYANVQLIVEIAERTH
VSAVWPGWGHASENPELPDALKEKGIIFLGPPSAAMAALGDKIGSSLIAQAAGVPTLPWS
GSHVKIPPESCNSIPEEMYRSACVSTTEEAVASCQVVGYPAMIKASWGGGGKGIRKVHND
DEVRALFKQVQGEVPGSPIFIMKVASQSRHLEVQLLCDKHGNVAALHSRDCSVQRRHQKI
IEEGPITVAPSETVKELEQAARRLAKCVHYVGAATVEYLYSMETGEYYFLELNPRLQVEHP
VTEWIAEINLPAAQVVVGMGVPLYNIPEIRRFYGMEHGGGYDAWRKISAVATKFDLDNAQ
SVKPKGHCVAVRVTSEDPDDGFKPTSGRVEELNFKSKPNVWAYFSVKSGGAIHEFSDSQFG
HVFAFGESRSLAIANMVLGLKEIQIRGEIRTNVDYTVDLLNAAEYRENKIHTGWLDSRIAM
RVRAERPPWYLSVVGGALYEASSRSSSVVTDYVGYLSKGQIPPKHISLVNLTVTLNIEGSK
YTIETVRRGPRSYTLRMNGSEIEAEIHSLRDGGLLMQLDGNSHVIYAETEAAGTRLLINGR
TCLLQKEHDPSKLLADTPCKLLRFLVADGSHVDADTPYAEVEVMKMCMPLLLPASGVIHF
VMPEGQAMQAADLIARLDLDDPSSVRRAEPFHGTFPKLGPPTAVSGKVHQKFAASVNSA
HMILAGYEHNINEVVQDLLNCLDSPELPFLQWQELMSVLATRLPKDLRNELDGKYKEYE
LNSDFRKNKDFPAKLLRGIIEANLAYCSEKDRVTNERLVEPLMSLVKSYEGGRESHARVVV
KSLFEEYLSVEELFSDNIQSDVIERLRLQHAKDLEKVVYIVFSHQGVRTKNKLILRLMEAL

VYPNPSAYRDQLIRFSGLNNTVYSELALKASQLLEHTKLSELRTSIARSLSELEMFTEEGERVST
PRRKMAINERMEDLVGAPLAVEDALVALFDHSDPTLQRRVVETYIRRLYQPYLVKGS
VRMQWHRSGLIALWEFSEEHIKQRNGQDAMSLKQQVEDPEEKRWGVMVVIKSLQYLSS
AIDAALKETSHYKAGAGNVSNGNSASSSHGNMLHIALVGINNQMSTLQDSGDEDQAQER
INKISKILKDSTVTSHLNGAGVRVVSCIIQRDEGRPPMRHSFQWSVDKIYYEEDPMLRHVE
PPLSTFLELNKVNLDGYNEVKYTPSRDRQWHIYTLIKNKKDQRSNDQRLFLRTIVRQPGV
TNGFLSGNVDNEVGRAQASSSYTSSSILRSLMAALEEIELHAHNETVRSSYSHMYLCILRV
QQLFDLIPFSRTIDNVGQDEATACTLLKNMALNIYEHVGVRMHRLSVCQWEVKLWLDCD
GQASGAWRVVVTNVTGHTCTVDIYREVEDSNTHKLFYHSVTPSLGPLHGIVLDEPYKPLD
AIDLKRYSARKNETTYCYDFPLAFETALKRSWKSTLSVVAEANEHNKSYAKVTELMFADS
TGSWGTPLVPVERSPGINDIGIVAWIMKLSTPEFPSGREIIVVSNDVTFKAGSFGPREDAFFD
AVTNLACERKLPLIYLSATAGARLGVAEEIKACFNVGWSDDESPERGFHYIYLTEQDYSRL
SSSVIAHELKLESGETRWVVDTIVGKEDGLGCENLHGSGAIASAYSKAYKETFTLTFVTGR
AVGIGAYLARLGMRCIQRLDQPIILTGFSALNKLLGREVYSSHMQLGGPKIMATNGVVHLT
VSDDLEGVSAILKWLSYVPPYVGGPLPIMKPLDPPDRPVTYFPENSCDARAAICGVQDSQ
GKWMGGMFDRESFVETLEGWAKTVVTGRAKLGGIPVGVIAVETQTMMQVIPADPGQLD
SAERVVPQAGQVWFPDSATKTAQALLDFNREELPLFILANWRGFSGGQRDLFEGILQAGS
NIVENLRTYNQPAFVYIPMGGELRGGAWVVVDSKINPEHIEMYAERTAKGNVLEPEGLVEI
KFRPKELEECMLRLDPELIKLSTRLREMKKENAGLSEMDTTRRSIIARMKQLMPIYTQVAT
RFAELHDTSARMAAKGVIGKVVDWEESRSFFYRRLRRRVTEDALAKEIREAAGEQLSQKS
ALDYIKKWYLSSNGSDGNSEKWNNDEAFFAWKDDPTNYENQLEELKAERVSKWLSRLA
ESPDVKALPNGLSIVLNKMNPSKREQVIDGLRQLLG
>OsACC2
MTSTHVATLGVGAQAPPRHQKKSAGTAFVSSGSSRPSYRKNGQRTRSLREESNGGVSDSK
KLNHSIRQGLAGIIDLPNDAASEVDISHGSEDPRGPTVPGSYQMNGIINETHNGRHASVSK
VVEFCTALGGKTPIHSVLVANNGMAAAKFMRSVRTWANDTFGSEKAIQLIAMATPEDLRI
NAEHIRIADQFVEVPGGTNNNNYANVQLIVEIAERTGVSAVWPGWGHASENPELPDALTA
KGIVFLGPPASSMHALGDKVGSALIAQAAGVPTLAWSGSHVEVPLECCLDSIPDEMYRKA
CVTTTEEAVASCQVVGYPAMIKASWGGGGKGIRKVHNDDEVRTLFKQVQGEVPGSPIFIM
RLAAQSRHLEVQLLCDQYGNVAALHSRDCSVQRRHQKIIEEGPVTVAPRETVKELEQAAR
RLAKAVGYVGAATVEYLYSMETGEYYFLELNPRLQVEHPVTEWIAEVNLPAAQVAVGMG
IPLWQIPEIRRFYGMNHGGGYDLWRKTAALATPFNFDEVDSKWPKGHCVAVRITSEDPDD
GFKPTGGKVKEISFKSKPNVWAYFSVKSGGGIHEFADSQFGHVFAYGTTRSAAITTMALAL
KEVQIRGEIHSNVDYTVDLLNASDFRENKIHTGWLDTRIAMRVQAERPPWYISVVGGALY
KTVTANTATVSDYVGYLTKGQIPPKHISLVYTTVALNIDGKKYTIDTVRSGHGSYRLRMNG
STVDANVQILCDGGLLMQLDGNSHVIYAEEEASGTRLLIDGKTCMLQNDHDPSKLLAETP
CKLLRFLVADGAHVDADVPYAEVEVMKMCMPLLSPASGVIHVVMSEGQAMQAGDLIAR
LDLDDPSAVKRAEPFEDTFPQMGLPIAASGQVHKLCAASLNACRMILAGYEHDIDKVVPE
LVYCLDTPELPFLQWEELMSVLATRLPRNLKSELEGKYEEYKVKFDSGIINDFPANMLRVII
EENLACGSEKEKATNERLVEPLMSLLKSYEGGRESHAHFVVKSLFEEYLYVEELFSDGIQS
DVIERLRLQHSKDLQKVVDIVLSHQSVRNKTKLILKLMESLVYPNPAAYRDQLIRFSSLNH
KAYYKLALKASELLEQTKLSELRARIARSLSELEMFTEESKGLSMHKREIAIKESMEDLVT
APLPVEDALISLFDCSDTTVQQRVIETYIARLYQPHLVKDSIKMKWIESGVIALWEFPEGHF
DARNGGAVLGDKRWGAMVIVKSLESLSMAIRFALKETSHYTSSEGNMMHIALLGADNK

MHIIQESGDDADRIAKLPLILKDNVTDLHASGVKTISFIVQRDEARMTMRRTFLWSDEKLSYEE
EPILRHVEPPLSALLELDKLKVKGYNEMKYTPSRDRQWHIYTLRNTENPKMLHRVFF
RTLVRQPSVSNKFSSGQIGDMEVGSAEEPLSFTSTSILRSLMTAIEELELHAIRTGHSHMYL
HVLKEQKLLDLVPVSGNTVLDVGQDEATAYSLLKEMAMKIHELVGARMHHLSVCQWEV
KLKLDCDGPASGTWRIVTTNVTSHTCTVDIYREMEDKESRKLVYHPATPAAGPLHGVALN
NPYQPLSVIDLKRCSARNNRTTYCYDFPLAFETAVRKSWSSSTSGASKGVENAQCYVKAT
ELVFADKHGSWGTPLVQMDRPAGLNDIGMVAWTLKMSTPEFPSGREIIVVANDITFRAGSF
GPREDAFFEAVTNLACEKKLPLIYLAANSGARIGIADEVKSCFRVGWSDDGSPERGFQYIY
LSEEDYARIGTSVIAHKMQLDSGEIRWVIDSVVGKEDGLGVENIHGSAAIASAYSRAYKET
FTLTFVTGRTVGIGAYLARLGIRCIQRLDQPIILTGYSALNKLLGREVYSSHMQLGGPKIMA
TNGVVHLTVSDDLEGVSNILRWLSYVPAYIGGPLPVTTPLDPPDRPVAYIPENSCDPRAAIR
GVDDSQGKWLGGMFDKDSFVETFEGWAKTVVTGRAKLGGIPVGVIAVETQTMMQTIPA
DPGQLDSREQSVPRAGQVWFPDSATKTAQALLDFNREGLPLFILANWRGFSGGQRDLFEG
ILQAGSTIVENLRTYNQPAFVYIPMAAELRGGAWVVVDSKINPDRIECYAERTAKGNVLEP
QGLIEIKFRSEELQDCMSRLDPTLIDLKAKLEVANKNGSADTKSLQENIEARTKQLMPLYT
QIAIRFAELHDTSLRMAAKGVIKKVVDWEESRSFFYKRLRRRISEDVLAKEIRAVAGEQFS
HQPAIELIKKWYSASHAAEWDDDDAFVAWMDNPENYKDYIQYLKAQRVSQSLSSLSDSS
SDLQALPQGLSMLLDKMDPSRRAQLVEEIRKVLG
>OsACC3
MALQSLRGSMRSVVGKRICPLIEYAIFPPLPRIIVYASRRARMQRGNYSLIKKPKKVSTLRQ
YQSTKSPMYQSLQRICGVREWLNKYCMWKEVDEKDFGFEIGAFD
>OsACC4
MALQSLRGSMRSVVGKRICPLIEYAIFPPLPRIIVYASRRARMQRGNYSLIKKPKKVSTLRQ
YQSTKSPMYQSLQRICGVREWLNKYCMWKEVDEKDFGFEIGAFD
>OsACC5
MALQSLRGSMRSVVGKRICPLIEYAIFPPLPRIIVYASRRARMQRGNYSLIKKPKKVSTLRQ
YQSTKSPMYQSLKRICGVREWLNKYCMWKEVDEKDFGFEIGAFD
>ZmACC1
MEGNLHSAVPTASRTGETTRPRAHTGPPLVPHRHLHHPPLSRASALHRGGVPDADCGPRN
FIIGPTPVARPSPPPLGISASARRLPLPPLLRPAAARIQTPYVAFSAAFLPEIFRNFALFLWHCA
FEKALPLLPNRQRSPAGTTFPSSALPRPSNRRKSHTRSLRDGGNEVSDAKKHSQSVRQGLA
GIIDIPSDAVSEVDISHGPKDPRGPTDSYQMNGIINETHNGRHASGSMVVEFCAALGGKTPI
HSILVANNGMAATKFMRSVRTWANDTFGSEKAIHLIAMATPEDMRINAEHIRLADQFVEV
PGGTNNNNYANVQLIVEIAERVGVSAVWPGWGHASENPELPDALTAKGIVFLGPPATSMN
ALGDKVGSALIAQAAGVPTLAWSGSHVEVPLECCLDAIPEEMYRKACVTTTEEAVASCQV
VGYPAMIKASWGGGGKGIRKVHNDDEVRALFKQVQGEVPGSPIFIMRLASQSRHLEVQL
LCDQYGNVAALHSRDCSVQRRHQKIIEEGPVTVAPRETVKALEQAARRLAKAVGYVGAA
TVEYLYSMETGEYYFLELNPRLQVEHPVTEWIAEVNLPAAQVAVGMGIPLWQIPEIRRFYG
MDYGGGYDIWRKTAALAAPFNFDEVDSLWPKGHCVAVRITSEDPDDGFKPTGGKVKEISF
KSKPNVWAYFSVKSGGGIHEFADSQFGHVFAYGLSRPAAITNMSLALKEIQIRGEIHSNVD
YTVDLLNASDFRENKIHTGWLDTRIAMRVQAERPPWYISVVGGALYKTVTTNAATVSEY
VSYLTKGQIPPKHISLVNSTVNLNIEGSKYTIETVRTGHGRYKLRMNDSTVEANVQSLCDG
GLLMQLDGNSHVIYAEEEAGGTRLQINGKTCLLQNDHDPSKLLAETPCKLLRFLVADGAH
VGADVPYAEVEVMKMCMPLLSPASGVIHCMMSEGQALQAGDLIARLDLDDPSAVKRAEP

FDGMFPLMDLPVAASSQVHKRYAASLNAARMVLAGYEHNINEVVQDLVCCLDNPELPFLQW
DELMSVLATRLPRNLKSELEDKYKEYKLNFYHGKNKDFPSKLLRDIVEENLAYGSEK
EKATNERLVEPLMNLLKSYEGGRESHAHFVVKSLFEEYLTVEELFSDGIQSDVIETLRHQH
SKDLQKVVDIVLSHQGVRNKAKLVTALMEKLVYPNPGAYRDLLVRFSSLNHKRYYKLAL
KASELLEQTKLSELCSSIARSLSDLGMHKGEMTIKDSMEDLVSAPLPVEDALISLFDYSDPT
VQQKVIVTYISRLYQPHLVKDSIQMKFKESGAIVFWEFSEGHVDTRNGQGAILGGKRWGA
MVVLRSLESASTAIMAALKDSVQYNNSEVNTMHIVLLNAETESNISGTSDDQAQHRMEKL
TKILKDSSVASDLQAAGLKVISCIVQRDAGRMPMRHTFLWFDEKNCYEEEHILRHVEPPLS
ALLELGKLKVKGYNEMKYTPSRDRQWHIYTLRNTENPKMLHRVFFRTIVRQPNAGNKFT
SAQVSDTGLGCPEESLSFTSNSILRSLMTAIEELELHAIRTGHSHMFLCILKEQKLLDLVPFS
GSTIVDVGQDEATACSLLRSMALKIHELVGARMHHLSVCQWEVKLKLDCDGPASGTWRV
VTTNVTSHTCTIDIYREVEDTESQKLLYHSATSSAGPMHGVALNNPYQPLSVIDLKRCSAR
NNRTTYCYDFPLAFETALQKSWQSNCSSVPEGSENSKSYVKSTELVFAEKHGSWGTPIIPM
ERPAGLNDIGMVAWILEMSTPEFPNGRQIIVVANDITFRAGSFGPREDAFFEAVTNLACERK
LPLIYLAANSGARIGIADEVKSCFRVGWSDERSPERGFQYIYLTEEDYARISSSVIAHKLQL
DNGEIRWIIDSVVGKEDGLGVENIHGSAAIASAYSRAYEETFTLTFVTGRTVGIGAYLARLG
IRCIQRLDQPIILTGFSALNKLLGREVYSSHMQLGGPKIMATNGVVHLTVSDDLEGVSNILR
WLSYVPANIGGPLPITKPLDPPDRPVAYIPENTCDPRAAIRGVDDSQGKWLGGMFDKDSFV
ETFEGWAKTVVTGRAKLGGIPVGVIAVETQTMMQLIPADPGQLDSHERSVPRAGQVWFP
DSATKTAQALLDFNREGLPLFILANWRGFSGGQRDLFEGILQAGSAIVENLRTYNQPAFVY
IPMAGELRGGAWVVIDSKINPDRIECYAERTAKGNVLEPQGLIEIKFRSEELQDCMGRLDP
ELINLKAKLQDAKHGNGSLPDIESLQKSIEARTKQLLPLYTQIAVRFAELHDTSLRMAAKG
VIKKVVDWEESRSFFYKRLRRRISEDLLAKEIRRIIGDNFTHQSAMELINEWYLASQATTGS
TAGWDDDDAFVAWKDSPENYKGYIQELRAQKVSQSLSDLADSSSDLQAFSQGLSTLLDK
MDPSQRVKFVQEVKKVLG
>ZmACC2
MSQLGLAAAASKALPLLPNRQRSSAGTTFSSSSLSRPLNRRKSRTRSLRDGGDGVSDAKK
HSQSVRQGLAGIIDLPSEAPSEVDISHGSEDPRGPTDSYQMNGIINETHNGRHASVSKVVEF
CAALGGKTPIHSILVANNGMAAAKFMRSVRTWANDTFGSEKAIQLIAMATPEDMRINAEH
IRIADQFVEVPGGTNNNNYANVQLIVEMAQKLGVSAVWPGWGHASENPELPDVLTAKGI
VFLGPPASSMNALGDKVGSALIAQAAGVPTLAWSGSHVEVPLECCLDAIPEEMYRKACV
TTTEEAVASCQVVGYPAMIKASWGGGGKGIRKVHNDDEVRALFKQVQGEVPGSPIFVMR
LASQSRHLEVQLLCDQYGNVAALHSRDCSVQRRHQKIIEEGPVTVAPRETVKALEQAARR
LAKAVGYVGAATVEYLYSMETGDYYFLELNPRLQVEHPVTEWIAEVNLPAAQVAVGMGI
PLWQIPEIRRFYGMDYGGGYDIWRKTAALATPFNFDEVDSQWPKGHCVAVRITSEDPDDG
FKPTGGKVKEISFKSKPNVWAYFSVKSGGGIHEFADSQFGHVFAYGLSRSAAITNMTLALK
EIQIRGEIHSNVDYTVDLLNASDFRENKIHTGWLDTRIAMRVQAERPPWYISVVGGALYKT
VTTNAATVSEYVSYLTKGQIPPKHISLVNSTVNLNIEGSKYTIETVRTGHGSYRLRMNDST
VEANVQSLCDGGLLMQLDGNSHVIYAEEEAGGTRLQIDGKTCLLQNDHDPSKLLAETPC
KLLRFLVADGAHVDADVPYAEVEVMKMCMPLLSPASGVIHCMMSEGQALQAGDLIARL
DLDDPSAVKRAEPFDGIFPQMELPVAVSSQVHKRYAASLNAARMVLAGYEHNINEVVQDL
VCCLDNPELPFLQWDELMSVLATRLPRNLKSELEDKYKEYKLNFYHGKNEDFPSKLLRDI
IEENLSYGSEKEKATNERLVEPLMNLLKSYEGGRESHAHFVVKSLFEEYLTVEELFSDGIQS
DVIETLRHQHSKDLQKVVDIVLSHQGVRNKAKLVTALMEKLVYPNPGGYRDLLVRFSSLN

HKRYYKLALKASELLEQTKLSELRASVARSLSDLGMHKGEMSIKDNMEDLVSAPLPVEDALIS
LFDYSDRTVQQKVIETYISRLYQPHLVKDSIQMKFKESGAITFWEFYEGHVDTRNGH
GAIIGGKRWGAMVVLKSLESASTAIVAALKDSAQFNSSEGNMMHIALLSAENESNISGISS
DDQAQHKMEKLSKILKDTSVASDLQAAGLKVISCIVQRDEARMPMRHTFLWLDDKSCYE
EEQILRHVEPPLSTLLELDKLKVKGYNEMKYTPSRDRQWHIYTLRNTENPKMLHRVFFRTI
VRQPNAGNKFTSAQISDAEVGCPEESLSFTSNSILRSLMTAIEELELHAIRTGHSHMYLCIL
KEQKLLDLIPFSGSTIVDVGQDEATACSLLKSMALKIHELVGARMHHLSVCQWEVKLKLD
CDGPASGTWRVVTTNVTGHTCTIDIYREVEEIESQKLVYHSATSSAGPLHGVALNNPYQPL
SVIDLKRCSARNNNRTTYCYDFPLAFETALQKSWQSNGSTVSEGNENSKSYVKATELVFAE
KHGSWGTPIIPMERPAGLNDIGMVAWIMEMSTPEFPNGRQIIVVANDITFRAGSFGPREDAF
FETVTNLACERKLPLIYLAANSGARIGIADEVKSCFRVGWSDEGSPERGFQYIYLTEEDYA
RISSSVIAHKLELDSGEIRWIIDSVVGKEDGLGVENIHGSAAIASAYSRAYEETFTLTFVTGR
TVGIGAYLARLGIRCIQRLDQPIILTGFSALNKLLGREVYSSHMQLGGPKIMATNGVVHLT
VPDDLEGVSNILRWLSYVPANIGGPLPITKPLDPPDRPVAYIPENTCDPRAAICGVDDSQGK
WLGGMFDKDSFVETFEGWAKTVVTGRAKLGGIPVGVIAVETQTMMQIIPADPGQLDSHE
RSVPRAGQVWFPDSATKTAQALLDFNREGLPLFILANWRGFSGGQRDLFEGILQAGSTIVE
NLRTYNQPAFVYIPMAGELRGGAWVVVDSKINPDRIECYAERTAKGNVLEPQGLIEIKFRS
EELQDCMGRLDPELINLKAKLQDVNHGNGSLPDIEGIRKSIEARTKQLLPLYTQIAIRFAEL
HDTSLRMAAKGVIKKVVDWEESRSFFYKRLRRRIAEDVLAKEIRQIVGDKFTHQLAMELI
KEWYLASQATTGSTGWDDDDAFVAWKDSPENYKGHIQKLRAQKVSHSLSDLADSSSDL
QAFSQGLSTLLDKMDPSQRAKFVQEVKKVLD
>ZmACC3
MAEPYQLNGILNGMPNLSHPSSPSEVDEFCKALGGNSPIHSVLVANNGMAAVKFMRSIRIW
ALETFGTEKAILLVAMATPEDLKINAEHIRIADQFIEVPGGTNNNNYANVQLIVEIAERTRVS
AVWPGWGHASENPELPDALDEKGIIFLGPPSAAMAALGDKIGSSLIAQAAGVPTLPWSGS
HVKVPPESCHSIPEELYRNACVSTTEEAVASCQVVGYPAMIKASWGGGGKGIRKVHNDDE
VRALFKQVQGEVPGSPIFIMKVASQSRHLEVQLLCDKHGNVAALHSRDCSVQRRHQKIIEE
GPITIAAPDTVKELEQAARQLAKCVQYVGAATVEYLYSMETGEYYFLELNPRLQVEHPVT
EWIAEINLPAAQVAVGMGIPLYNIPEIRRFYGMDHGGGYHNWRTISAVATKFDLDKAQSVR
PKGHCVAVRVTSEDPDDGFKPTSGRVEELNFKSKPNVWAYFSVKSGGAIHEFSDSQFGHVF
AFGESRSLAIANMVLGLKEIQIRGEIRTNVDYTVDLLNATEYRENKIHTGWLDSRIAMRVR
AERPPWYLSVVGGALYEASSRSSSVITDYVGYLSKGQIPPKHISLVNLTVTLNIEGSKYTIET
VRGGPRSYKLRMNGSEIETEIHSLRDGGLLMQLDGNSHVIYAETEAAGTRLLINGRTCLLQ
KEHDPSKLLADTPCKLLRFLVADGSHVDADTPYAEVEVMKMCMPLLLPASGVIHFVMPE
GQAMKANDLIARLDLDDPSSVRRAEPFHGSFPKLGPPTAISGKVHQKFAASVNSAHMILA
GYEHNINEVVQDLLNCLDNPELPFLQWQELMSVLATRLPKDLRNELDGKYKEYELNPDF
CKSKDFPARLLRGVIEANLAYCSEKDRVTNERLVEPLMSLVKSYEGGRESHARVVVKSLFE
EYLSVEELFNDNLQSDVIERLRLQHAKDLEKVVYIVFSHQGVRSKNKLILRLMEALVYPN
PSAYRDQLIRFSALNHTSYSELALKASQLLEHTKLSELRTSIARSLSELEMFTEEGERLSTPR
RKMAINERMEDLVCAPLAAEDALVALFDHSDPTLQRRVVETYIRRLYQPYLVSGSIRMQW
HRAGLIALWEFSEEHLKQRSGQDVPLQQVENPIEKSWGVMVVIKSLQFVATAIDVALKETS
QYGIGVRSVSNSNHVHSNQSNMLHIALVGINNQMSTLQDSGDEDQTQERVNKLFKILKDN
TITSHLNGASVKVVSCIIQRDEGRPPMRHSFQWSVDKLYYEEDPMLRHVEPPLSTFLELEK
VNLEGYNEVKYTPSRDRQWHIYTLIKNKKDQRLNDQRMFLRTIVRQPSATNSFLTGNIDN

EVGHTQASSSFTSNSILRSLMGALEEIELHAHSETVRSGHSHMYLCLLREQQLHELIPFSRMTG
KIDKDEGTVCTLLKHMVLNLYEHVGVRMHRLSVCQWEVKLWLVCDGQASGAWRV
VVTNVTGHTCTIDIYREVEDPTTHQLLYHSATTSAGPLHGVALNEPYKPLDAIDLKRYAAR
KNETTYCYDFPLAFETALKRSWKSSSYGVSEANEHNRFYAEVKELIFADSVGAWGTPLVS
VERPPGINDIGIVAWNMKLSTPEFPRGREIIVVANDVTFKAGSFGPREDAFFDAVTNLACER
KLPLIYLAATAGARLGVAEEIKSCFHVGWSDYESPERGFQYIYLTTQDYSRLSSSVIAHELQ
LKNGETRWVVDTIVGKEDGLGCENLHGSGAIASAYSKAYKETFTLTFVTGRAVGIGAYLA
RLGMRCIQRLDQPIILTGFSALNKLLGREVYSSHMQLGGPKIMATNGVVHQTVSDDLEGV
SAILKWLSYVPPYVGGPLPIMKPLDPPERPVTYLPENACDALAAICGIQDGEGRWLGGMF
DRESFVETLEGWAKTVITGRAKLGGIPVGVIAVETQTVMQVIPADPGQLDSAERVVPQAG
QVWFPDSATKTAQALLDFNREELPLFILANWRGFSGGQRDLFEGILQAGSTIVENLRTYKQ
PAFVYIPMGGELRGGAWVVVDSKINPDHIEMYAERTAKGNVLEPEGLVEIKFRPKELEDC
MLRLDPELIGLNTRLKEMKKQNASISEMETIRRSMTIRMKQLMPIYTQVATRFAELHDTSA
RMAAKGVIGKVVDWKESRAFFYRRLRRRVAEDALAKEVKEAAGEQLSHRSALDCIKKW
YLASKGTEGDGEMWNDDESFFAWKDDPKNYENYLEELKAERVSNWFSHLAESSDVKAL
PNGLSLLLNKMNPLKREQVIDGLRQLLG
>ZmACC4
MRCIQRLDQPIILTGFSALNKLLGREVYSSHMQLGGPKIMATNGVVHQTVSDDLEGVSAIL
KWLSYVPPYVGGPLPIMKPLDPPERPVTYLPENACDALAAICGIQDGEGRWLGGMFDRES
FVETLEGWAKTVITGRAKLGGIPVGVIAVETQTVMQVIPA
>ZmACC5
MRCIQRLDQPIILTGFSALNKLLGREVYSSHMQLGGPKIMATNGVVHQTVSDDLEGVSAIL
KWLSYVPPYVGGPLPIMKPLDPPERPVTYLPENACDALAAICGIQDGEGRWLGGMFDRES
FVETLEGWAKTVITGRAKLGGIPVGVIAVETQTVMQVIPA
>ZmACC6
MKSLFKSKIWWPHRSNDPTSTAGQSQQTSGPPTASSPSGTVPATALSVSTPASSSPPPVVATL
TGAAAAAVVAGAGEEDYISSKAKLMSLNCFAANRDETHTAESLSHRYWATEYHKNKIHT
GWLDSRIAMRVRAERPPWYLSVVGGALYEASSRSSSVITNYVGYLSKDQIPVVVGLKTSW
>ZmACC7
MRCIQRIDQPIILTGFSALNKLLGREVYSSHMQLGGPKIMATNGVVHQTVSDDLEGVSAIL
KWLSYVPPYVGGPLPIMKPLDPPERPVTYLPENACDALAAICGIQDGEGRWLGGMFDRES
FVETLEGWAKTVITGRAKLGGIPVGVIAVETQTVMQVIPA
>AhACC1
MAGVGRGNGYTNGVVPNRHPATISEVDEYCNALGGTRPIHSILIANNGMAAVKFIRSVRS
WAYETFGTEKAILLVAMATPEDMRINAEHIRIADQFVEVPGGTNNNNYANVQLIVEMAEIT
RVDAVWPGWGHASENPELPDALKAKGIVFLGPPAVSMAALGDKIGSSLIAQAAEVPTLPW
SGSHVKIPPDSCLVTIPDEIYREACVYTTEEAIASCQVVGYPAMIKASWGGGGKGIRKVHN
DDEVRALFKQVQGEVPGSPIFIMKVASQSRHLEVQLLCDQYGNVAALHSRDCSVQRRHQ
KIIEEGPITVAPPQTVKLLEQAARRLAKSVNYVGAATVEYLFSMETGEYYFLELNPRLQVE
HPVTEWIAEINLPAAQVAIGMGIPLWQLPEIRRFYGVEHGGGNDAWRKTSALATPFDFDK
AQSTKPKGHCVAVRVTSEDPDDGFKPTSGKVQELSFKSKPNVWAYFSVKSGGGIHEFSDS
QFGHVFAFGESRALAIANMVLGLKEIQIRGEIRTNVDYTIDLLNASDYRDNKIHTGWLDSR
IAMRVRAERPPWYLSVVGGALYKASASSAALVSDYVGYLEKGQIPPKHISLVHSQVSLNIE
GSKYTIDMVRGGSGSYRLRMNQSEVEAEIHTLRDGGLLMQASILDGNSHVIYAEEEAAGT

RLLIDGRTCLLQNDHDPSKLVAETPCKLMRYLVVDDSHIDADTPYAEVEVMKMCMPLLSPAS
GVIHFKMSEGQPMQAGELIARLDLDDPSAVRKAEPFNGKFPVLGPPTATSDKVHQKCA
ASLNAAQMILAGYEHNIDEVVQSLLNCLDSPELPFLQWQECFAVLANRLPKDLKNELESK
YKEYERISSFQVVDFPAKLLKGILEAHLSSCPNKEKGAQERLIEPLLSLVKSYEGGRESHAR
KIVQSLFEEYLFVEELFSDNIQADVIERLRLQYKKDLLKIVDIVLSHQGIKSKNKLILRLMD
KLVYPNPAAYRDQLIRFSQLNHTNYSQLALKASQLLEQTKLSELRSNIARSLSELEMFTED
GENIDTPKRKSAINDRMEDLVSAPLAVEDALVGLFDHSDHTLQRRVVETYIRRLYQPYLVK
GSVRMQWHRSGLIASWEFLEEYIERKSGVEDQMSDKTLVEKHTEKKWGVMVVIKSLHFL
PAIITAALKEATNNLHEAVSSAAGEPVKHGNMMHVALVGINNQMSLLQDSGDEDQAQERI
NKLAKILKEEEVGSTIRGTGVGVISCIIQRDEGRTPMRHSFHWSAEKLYYQEEPLLRHLEPP
LSIYLELDKLKGYENIRYTPSRDRQWHLYTVMDQKPQPVQRMFLRTLLRQPTTNEGFSSY
QRTDAETPSTELAMSFTSRSIFRSLMAAMEELELNSHNATIRPEHAHMYLYIIREQEINDLV
PYPKRVDIDAGQEETTVEATLEELAHEIHSSVGVRMHRLGVVVWEVKLWMAACGQANG
AWRIVVNNVTGHTCTVHIYREMEDTNTHRVVYSSITVKGPLHGVPVNETYQPLGVIDRKR
LSARKNSTTFCYDFPLAFETALEQSWAIQQPGFRRPKDKNLLKVTELRFADKEGSWGTPLV
PVEHSAGLNDVGMVAWFMDMCTPEFPSGRTILVVANDVTFKAGSFGPREDAFFRAVTDL
ACAKKLPLIYLAANSGARLGVAEEVKACFKVGWSEESNPEHGFQYVYLTPEDFARIGSSVI
AHELKLESGETRWIIDTIVGKEDGLGVENLSGSGAIAGSYSRAYKETFTLTYVTGRTVGIGA
YLARLGMRCIQRLDQPIILTGFSALNKLLGREVYSSHMQLGGPKIMATNGVVHLTVSDDL
EGVSAILKWLSYIPSHVGGSLPIVKPLDPPERPVEYLPENSCDPRAAISGTLDGNGRWLGGI
FDKDSFVETLEGWARTVVTGRAKLGGIPVGIVAVETQTVMQIIPADPGQLDSHERVVPQAG
QVWFPDSATKTAQAIMDFNREELPLFILANWRGFSGGQRDLFEGILQAGSTIVENLRTYKQ
PIFVYIPMMGELRGGAWVVVDSRINSDHIEMYADRTAKGNVLEPEGMIEIKFRTRELLECM
GRLDQKLITLKAKLQEAKDKRDTESFESLQQQIKSREKQLLPLYTQIATKFAELHDTSLRM
AAKGVIRQVLDWGNSRAVFYRRLYRRIGEQSLINNVREAAGDHLSHVSAMDLVKNWYLS
SNIAKGRKDAWLDDEAFFSWKENPLNYEDKLKELRAQKVLLQLTNIGDSVLDLQALPQG
LAALLSKLEPSSRVKLTEELRKVLG

>AhACC2

MAGVGRGNGYTNGVVPNRHPATISEVDEYCNALGGTRPIHSILIANNGMAAVKFIRSVRS
WAYETFGTEKAILLVAMATPEDMRINAEHIRIADQFVEVPGGTNNNNYANVQLIVEMAEIT
RVDAVWPGWGHASENPELPDALKAKGIVFLGPPAVSMAALGDKIGSSLIAQAAEVPTLPW
SGSHVKIPPDSCLVTIPDEIYREACVYTTEEAIASCQVVGYPAMIKASWGGGGKGIRKVHN
DDEVRALFKQVQGEVPGSPIFIMKVASQSRHLEVQLLCDQYGNVAALHSRDCSVQRRHQ
KIIEEGPITVAPPQTVKQLEQAARRLAKSVNYVGAATVEYLFSMETGEYYFLELNPRLQVE
HPVTEWIAEINLPAAQVAIGMGIPLWQLPEIRRFYGVEHGGGNDAWRKTSALATPFDFDK
AQSTKPKGHCVAVRVTSEDPDDGFKPTSGKVQELSFKSKPNVWAYFSVKSGGGIHEFSDS
QFGHVFAFGESRALAIANMVLGLKEIQIRGEIRTNVDYTIDLLNASDYRDNKIHTGWLDSR
IAMRVRAERPPWYLSVVGGALYKASASSAALVSDYVGYLEKGQIPPKHISLVHSQVSLNIE
GSKYTIDMVRGGSGSYRLRMNQSEVEAEIHTLRDGGLLMQASILDGNSHVIYAEEEAAGT
RLLIDGRTCLLQNDHDPSKLVAETPCKLMRYLVVDDSHIDADTPYAEVEVMKMCMPLLSP
ASGVIHFKMSEGQPMQAGELIARLDLDDPSAVRKAEPFNGKFPVLGPPTATSDKVHQKCA
ASLSAAQMILAGYEHNIDEVVQSLLNCLDSPELPFLQWQECFAVLANRLPKDLKNELESK
YKEYERISSFQVVDFPAKLLKGILEAHLSSCPNKEKGAQERLIEPLLSLVKSYEGGRESHAR
KIVQSLFEEYLFVEELFSDNIQADVIERLRLQYKKDLLKIVDIVLSHQGIKSKNKLILRLMD

KLVYPNPAAYRDQLIRFSQLNHTNYSQLALKASQLLEQTKLSELRSNIARSLSELEMFTEDGEN
IDTPKRKSAINDRMEDLVSAPLAVEDALVGLFDHSDHTLQRRVVETYIRRLYQPYLVK
GSVRMQWHRSGLIASWEFLEEYIERKSGVEDQMSDKTLVEKHTEKKWGVMVVIKSLHFL
PAIITAALKEATNNLHEAVSSAAGEPVKHGNMMHVALVGINNQMSLLQDSGDEDQAQERI
NKLAKILKEEEVGSTIRGTGVGVISCIIQRDEGRTPMRHSFHWSAEKLYYQEEPLLRHLEPP
LSIYLELDKLKGYENIRYTPSRDRQWHLYTVMDQKPQPVQRMFLRTLLRQPTTNEGFSSY
QRTDAETPSTELAMSFTSRSIFRSLMAAMEELELNSHNATIRPEHAHMYLYIIREQEINDLV
PYPKRVDIDAGQEETTVEATLEELAHEIHSSVGVRMHRLGVVVWEVKLWMAACAQANG
AWRIVVNNVTGHTCTVHIYREMEDTNTHRVVYSSITVKGPLHGVPVNETYQPLGVIDRKR
LSARKNSTTFCYDFPLAFETALEQSWAIQQPGFRRPKDKNLLKVTELRFADKEGSWGTPLV
PVEHSAGLNDVGMVAWFMDMCTPEFPSGRTILVVANDVTFKAGSFGPREDAFFRAVTDL
ACAKKLPLIYLAANSGARLGVAEEVKACFKVGWSEESNPEHGFQYVYLTPEDFARIGSSVI
AHELKLESGETRWIIDTIVGKEDGLGVENLSGSGAIAGSYSRAYKETFTLTYVTGRTVGIGA
YLARLGMRCIQRLDQPIILTGFSALNKLLGREVYSSHMQLGGPKIMATNGVVHLTVSDDL
EGVSAILKWLSYIPSHVGGSLPIVKPLDPPERPVEYLPENSCDPRAAISGTLDGNGRWLGGI
FDKDSFVETLEGWARTVVTGRAKLGGIPVGIVAVETQTVMQIIPADPGQLDSHERVVPQAG
QVWFPDSATKTAQAIMDFNREELPLFILANWRGFSGGQRDLFEGILQAGSTIVENLRTYKQ
PIFVYIPMMGELRGGAWVVVDSRINSDHIEMYADRTAKGNVLEPEGMIEIKFRTRELLECM
GRLDQKLITLKAKLQEAKDKRDTESFESLQQQIKSREKQLLPLYTQIATKFAELHDTSLRM
AAKGVIRQVLDWGNSRAVFYRRLYRRIGEQSLINNVREAAGDHLSHVSAMDLVKNWYLS
SNIAKGRKDAWLDDEAFFSWKENPSNYEDKLKELRAQKVLLQLTNIGDSVLDLQALPQG
LAALLSKLEPSSRVKLAEELRKVLG
>AhACC3
MASSFASTASSASSSLPTPSKPKPKINHFRFSHSNLSFRLSPKPNLPFLTKGSPPCQIVCPRVK
AQLDEVSLDGSSNAVAPTTANSEAEATAKPSSGTSSGVLASQESISQFITQVASLVKLVDSR
DIVELQLKQHDCEVMIRKREAMPQPQPPAQPAMYYPPPSLAAPPAAPASSPAPAPTPATRA
ASASPPAAKSTKSSLPPLKCPMAGTFYRSPGPGEPPFVKVGDKVKKGQVLCIIEAMKLMN
EIEADQSGTIVEILAEDGKPVSVDMPLFVIEP
>AhACC4
GKIGILSSFEYLYSPLALFGKKKAAPPPPSKKAATAVTPANDELAKWYGKFSGALHATCQG
DKILVANRGKIPVRAIRTAHELGIPCVAVYSTIDKDALHVKLADESVYIGL
>AhACC5
MASCTIPCPKCLSFSHLGLNSQTTQRNMHVAGLGLKKSQSFGSLVCDSNSIGVQCLNTKKF
SALKCQAQPKEVVTLENSSNSAPALVNGPIPASSSKEKDDENRKPAGPSTFADPASMSAFM
NQVSDLVKLVDSRDIVELQLKQADCELMIRKKEALEPPPAMVAPVSFPYPTYPSMPSPPPP
AAAAPAPASAAPSKAAPALPPPAKASRSSHPPLKCPMAGTFYRSPAPGEPPFVKVGDKVQK
GQVICIVEAMKLMNEIEADQSGTITEIIAEDGKPVSVDTPLLVIVP
>AhACC6
MASFTVPCPKCPSPSLGLNSQKLLKPSLSFGSLAAESASSGIRCLNGKQFSVQKLQAQRRE
AVTTIENSAPVLVSGPKVAAPNEKEDQNGKPGGTTTDPSLVSAFMAQVADLVKLVDSRDIV
ELQLKQSDCELMIRKKEALEPPPQVIAPASAPMHYAAYPSPPPPPPPVAASSTPASSPPAKAA
PALPSPGKASTSGHPPLKCPMAGTFYRSPAPGEPPFVKVGDKVQKGQVICIIEAMKLMNEI
EADQTGTITEILAEDGKPVSVDTPLLVIAP
>AhACC7

MASFTIPCPKCLSFSHLGLNSQTTQRNMHVAGLELKKSQSFGSLVCDSNSIGVQCLNTKKFSAL
KCQAQPKEVVTLENSSNSAPALVNGPIPASSKEKDDENRKPSGPSTFADPASMSAFMN
QVSDLVKLVDSRDIVELQLKQADCELMIRKKEALEPPPAMVAPVSFPYPTYPSMPSPPPPA
AAAVAPASAAPSKAAPALPPPAKASRSSHPPLKCPMAGTFYRSPAPGEPPFVKVGDKVQKG
QVVCIVEAMKLMNEIEADQSGTITEIIAEDGKPVSVDTKFLV
>AhACC8
MESRIMAALNSVTSPHLPSHSPGLYAVENSIKSSQCSFSAGSKKVSFPRQRCSHVTKTRAA
RDGGAGGALGATCQAEKILVANRGEIAVRVIRTAHEMGIPCVAVYSTIDKDALHVKLADDA
VCIGEAPSSQSYLLIPNVLSAAISRRCTMLHPGYGFLAENAVFVEMCREHGINFIGPNPDSIR
VMGDKSTARDTMKKAGVPTVPGSDGLLQTTEEAIRLANEIGFPVMIKATAGGGGRGMRL
AKEPDEFVKLLQQAKSEAAAAFGNDGVYLEKYVQNPRHIEFQVLADKYGNVVHFGERD
CSIQRRNQKLLEEAPSPALTPELRKAMGDAAVAAAASIGYIGVGTVEFLLDERGSFYFMEM
NTRIQVEHPVTEMISSVDLIEEQIRVAMGEKLRYKQEDIVLRGHSIECRINAEDAFKGFRPG
PGRITAYLPSGGPFVRMDSHVYPDYVVPPSYDSLLGKLIVWAPTREKAIERMKRALDDTII
TGKSTCIVIYSIAYQYLVQANCTPEARRD
>AhACC9
MEATMAACNSLSSPSVPIPGLYAGTSRGIKNSQCSFLGATKVNFPSQTMPGTCQLNHKHKT
RSGALHATCQGDKILVANRGEIAVRVIRTAHELGIPCVAVYSTIDKDALHVKLADESVCIGE
APSSQSYLLIPNVLSAAISRRCTMLHPGYGFLAENAVFVEMCREHGINFIGPNPDSIRVMGD
KSTARDTMKNAGVPTVPGSDGLLQSTEEAIRLANEIGFPVMIKATAGGGGRGMRLAKEPG
EFVKLLQQAKSEAAAAFGNDGVYLEKYIQNPRHIEFQVLADKYGNVVHFGERDCSIQRR
NQKLLEEAPSPALTPELRKAMGDAAVAAAASIGYIGVGTVEFLLDERGSFYFMEMNTRIQ
VEHPVTEMISSVDLIEEQIRVAMGAKLRYKQEDIILRGHSIECRINAEDAFKGFRPGPGRITA
YLPSGGPFVRMDSHVYPDYVVPPSYDSLLGKLIVWAPTREKAIERMKRALDDTIITGVPTT
IEYHKLILDIEDFRNGKVDTAFIPKHEEELTMPPQKMVPAINKAKELVGATV
>AhACC10
MASSFASTASSASSSLPTPSKPKPKINHFRFSHSNLSFRLSPKPNLPFLTKGSPPCQIVCPRVK
AQLNEVSLDGSSNAVAPTTANSEAEATAKPSSGTSSGVLASQESISQFITQVASLVKLVDSR
DIVELQLKQHDCEVMIRKREAMPQPQPPAQPAMYYPPPSLAAPPVAPASSPAPAPTPATRA
ASASPPAAKSTKSSLPPLKCPMAGTFYRSPGPGEPPFVKVGDKVKKGQVLCIIEAMKLMN
EIEADQSGTIVEILAEDGKPVSVDMPLFVIEP
>AhACC11
MESRIMAALNSVTSPHLPSHSPGLFAVENSIKSSQCSFSAGSKKVSFPRQRCSHVTKTRAAR
DGGAGGALGATCQAEKILVANRGEIAVRVIRTAHEMGIPCVAVYSTIDKDALHVKLADDAV
CIGEAPSSQSYLLIPNVLSAAISRRCTMLHPGYGFLAENAVFVEMCREHGINFIGPNPDSIRV
MGDKSTARDTMKKAGVPTVPGSDGLLQTTEEAIRLANEIGFPVMIKATAGGGGRGMRLA
KEPDEFVKLLQQAKSEAAAAFGNDGVYLEKYVQNPRHIEFQVLADKYGNVVHFGERDC
SIQRRNQKLLEEAPSPALTPELRKAMGDAAVAAAASIGYIGVGTVEFLLDERGSFYFMEMN
TRIQVEHPVTEMISSVDLIEEQIRVAMGEKLRYKQEDIVLRGHSIECRINAEDAFKGFRPGP
GRITAYLPSGGPFVRMDSHVYPDYVVPPSYDSLLGKLIVWAPTREKAIERMKRALDDTIIT
GVPTTIDYHKLILDIEDFKNGKVDTAFIPKHEEELAMPPVKMVLAKEFAGVNA
>AhACC12
MGPVVQVQVQAELSICYPPPPHRFHRRSSSHKKPPFVPTLIFAFSLTYSFSQEMEATMAAC
NSLSSPSVPIPGLYAGTSRGIKNSQCSFLGATKVNFPSQTMSRTCQLNHKHKTRSGALHATC

QGDKILVANRGEIAVRVIRTAHELGIPCVAVYSTIDKDALHVKLADESVCIGEAPSSQSYLLIPN
VLSAAISRRCTMLHPGYGFLAENAVFVEMCREHGINFIGPNPDSIRVMGDKSTARDTM
KNAGVPTVPGSDGLLQSTEEAIRLANEIGFPVMIKATAGGGGRGMRLAKEPGEFVKLLQQ
AKSEAAAAFGNDGVYLEKYIQNPRHIEFQVLADKYGNVVHFGERDCSIQRRNQKLLEEA
PSPALTPELRKAMGDAAVAAAASIGYIGVGTVEFLLDERGSFYFMEMNTRIQVEHPVTEMI
SSVDLIEEQIRVAMGAKLRYKQEDIILRGHSIECRINAEDAFKGFRPGPGRITAYLPSGGPFV
RMDSHVYPDYVVPPSYDSLLGKLIVWAPTREKAIERMKRALDDTIITGVPTTIEYHKLILDI
EDFRNGKVDTAFIPKHEEELTMPPQKMVPAINKAKEFVGATVSSHGAPVAPALSGGGKEG
QLQSLQQFGILTAMGALVHCLCGTGNRLSFH
>AhACC13
MASFTVPCPKCPSPSLGLNSQKLFKPSLSFGSLAAESASSGIRCLNGKQFSVQKLQAQRRE
AVTTIENSAPVLVSGPKVAAPNEKEDQNGKPGGTTTDPSLVSAFMAQVADLVKLVDSRDIV
ELQLKQSDCELMIRKKEALEPPSQVIAPASAPMHYAAYPSLPPPPPPAAASSTPASSPPAKA
APALPSPGKTSTSGHPPLKCPMAGTFYRSPAPGEPPFVKVGDKVQKGQVICIIEAMKLMNE
IEADQTGTITEILVEDGKPVSVDTPLLVIAP
>AhACC14
MESRIMAALNSVTSPHLPSHSPGLYAVENSIKSSQCSFSAGSKKVSFPRQRCSHVTKTRAA
RDGGAGGALGATCQAEKILVANRGEIAVRVIRTAHEMGIPCVAVYSTIDKDALHVKLADDA
VCIGEAPSSQSYLLIPNVLSAAISRRCTMLHPGYGFLAENAVFVEMCREHGINFIGPNPDSIR
VMGDKSTARDTMKKAGVPTVPGSDGLLQTTEEAIRLANEIGFPVMIKATAGGGGRGMRL
AKEPDEFVKLLQQAKSEAAAAFGNDGVYLEKYVQNPRHIEFQVLADKYGNVVHFGERD
CSIQRRNQKLLEEAPSPALTPELRKAMGDAAVAAAASIGYIGVGTVEFLLDERGSFYFMEM
NTRIQVEHPVTEMISSVDLIEEQIRVAMGEKLRYKQEDIVLRGHSIECRINAEDAFKGFRPG
PGRITAYLPSGGPFVRMDSHVYPDYVVPPSYDSLLGKLIVWAPTREKAIERMKRALDDTII
TGVPTTIDYHKLILDIEDFKNGKVDTAFIPKHEEELAMVTF
>AhACC15
MNTMSLTRGNYYMDGRLIIEEHGIESANQCVGKDFLGGSRAICIWPKYLTSSRNNTNTNP
NNNGKWQRFNVAAKIRKGKKHDYPWPDKMDPNISSGYLTYLSHFKPLAEKPKPVTLDFE
KPLVDLEKKIIEVRSMADDTGLDFSNQIEALESKYQQALKDLYTHLTPFQRLMIARHPNRP
TVLDHILNITEKWVELHGDRAGYDDPAIVTGLGTMDGKSYMFIGHQKGRNTKENITRNFA
MPTPHGYRKALRMMKYADHHKFPIITFVDTPGAYADLKSEELGQGEAIAHNLRTMFGLK
VPILTVVTGEGGSGGALAIACANKLFMLENSAFYVASPEACAAILWKSSKAAPKAAEKLRI
TAQEHYRLGIADGVIPEPLGGAHVDPTWTSQQIKLTLTQAMEELTKMNEEELFRHRHLKF
RSIGGFQEGIPVEPERKRNMKPSDVNSSTLTDIESELQTLRKQILESKGPTDPITNESIQKLV
KEVDEEITKAIISMGLAEKVQSVRMELSKNSNQPLSTNMEEKVDRIMEEINMKMAQPGAY
LGLKQKLKKLDTINSFIEMKVKQEKLRNELNEKLSADTKAKIASLMDAQERMPDHELVE
KAMEVQRELEEVLKSANLEIVGVMKKNVETPPADIKQKIVELNNEIIGEIDRVVNAEEGLK
DQIKELENMIAQGLDSKDVEKMEAGIKERILAALDAAGVKEKIERMKEEVESLSMAGFED
KIGEENGRC
>AhACC16
MASSSAAASLAGGSASDLLRSSTSGFSGVPLRTLGRARLPLKQRDFSVSCKMRKVKKHEH
PWPDNPDPNVKGGVLSHLSPFKPLKEKPKPVTLDFEKPLIALQKKIIDVRKMANETGLDFS
DQILSLETKYQQALKDLYTHLTPIQRVNIARHPNRPTFLDHIYNITDKFVELHGDRAGYDDP
AIVTGIGTIDGRRYMFIGQQKGRNTKENIQRNFGMPTPHGYRKALRLMEYADHHGFPIVT

FIDTPGAYADLKSEELGQGEAIAHNLRSMFGLKVPVISIVIGEGGSGGALAIGCGNKLLMLENA
VFYVASPEACAAILWKSAKAAPKAAEKLRITASELCRLEIADGVIPEPLGGAHADPSW
TSQQIKKAVNEAMDELTKLNTEELLRHRMLKFRKIGGFQEGIPVEPKKKINMKKKDIPIAN
KISDAELEVEVEKLKQQILDSKESSIEPRLDLDDMIKQLQIEVDQEYSEAVNAIGLSDRMSK
LKEEVVKANTDNQFVDPLLKSKIEKLKEEFDQKLSTAPNFGRLENKVNMLKELSKVKRL
QDQNKRTSALEQELKTKFDGIMKNPRIKEKYEALKSEIQAAGASSSRDLDDDLKQKIVEF
NKEFDSLLAESLKSAGMEVKIAPARPRDSSEESAVLGYESKIEELREGISKEIEKLANSSNIK
SKIELLKLEVAKAGETPDTESKNRIAALVQQIKQSLEEAVDSSSLKEEYENLVSKISSRDLET
EDGLTNDQLREKVGANRSFS
>AhACC17
MASSSAAASLAGGSASDLLRGSTSGFSGVPLRTLGRARLPLKQRDFSVSCKMRKVKKHE
HPWPDNPDPNVKGGVLSHLSPFKPLKEKPKPVTLDFEKPLIALQKKIIDVRKMANETGLD
FSDQILSLETKYQQALKDLYTHLTPIQRVNIARHPNRPTFLDHIYNITDKFVELHGDRAGYD
DPAIVTGIGTIDGRRYMFIGQQKGRNTKENIQRNFGMPTPHGYRKALRLMEYADHHGFPI
VTFIDTPGAYADLKSEELGQGEAIAHNLRSMFGLKVPVISIVIGEGGSGGALAIGCGNKLL
MLENAVFYVASPEACAAILWKSAKAAPKAAEKLRITASELCRLEIADGVIPEPLGGAHADP
SWTSQQIKKAVNEAMDELTKMNTEELLRHRMLKFRKIGGFQEGIPVEPKKKINMKKKDIP
IANKISDAELEVEVEKLKQQILDSKESSVEPRLDLDDMIKQLQIEVDQEYSEAVNAIGLSDR
MSKLKEEVVKANTDNQFIDPLLKSKIEKLKEEFDQKLSTAPNFGRLENKVNMLKELSKVK
RLQDQNKRTSALEQELKTKFDGIMKNPRIKEKYEALKSEIQAAGASSSRDLDDDLKQKIV
EFNKEFDSLLAESLKSAGMEVKIAPARPRDSSGESAELGYESKIEELREGISKEIEKLANSSN
IKSKIELLKLEVAKAGETPDTESKNRIAALVQQIKQSLEEAVDSSSLKEEYENLVSKISSRDS
EVEDGLTNDQLREKVGANRSFS
>AhACC18
MNTMSLTRGNYYMDGRLLIEEHGIESANQCVGKDFLGVSRAICIWPKYLTSSRNNTNTNT
NNNGKWQRFNVAAKIRKGKKHDYPWPDKMDPNISSGYLTYLSHFKPLAEKPKPVTLDFE
KPLVDLEKKIIEVRNMADDTGLDFSNQIEALESKYQQALKDLYTHLTPFQRLMIARHPNRP
TVLDHILNITEKWVELHGDRAGYDDPAIVTGLGTMDGKSYMFIGHQKGRNTKENITRNFA
MPTPHGYRKALRMMKYADHHKFPIITFVDTPGAYADLKSEELGQGEAIAHNLRTMFGLK
VPILTVVTGEGGSGGALAIACANKLFMLENSAFYVASPEACAAILWKSSKAAPKAAEKLRI
TAQEHYRLGIADGVIPEPLGGAHVDPTWTSQQIQLTLTQAMEELTKMNEEELLRHRHLKF
RSIGGFQEGIPVEPERKRNMKPSDVNSSTLTDIESELQTLRKQILESKGPTDPITNESIQKLV
KEVDEEITKAIISMGLAEKVQSVRMELSKNSNQPLSTNMEEKVDRIMEEINMKMAQPGAY
LGLKQKLKKLDTINSFIEMKVKQEKLRKELNEKLSADTKAKIASLMDAQERIPDHELVEK
AMEVQRELEEVLKSANLEIVGVMKKNVETPPADIKQKIVELNNEIIGEIDRVVNAEEGLKD
QIKELENMIAQGLDSKDAEKMEAGIKERILAALDAAGVKEKIERMKEEVESLSMAGFEDK
IGVENGRC
>AhACC19
MSSSDRIELSIDPGTWNPMDEDMVSMDPIEFHSEEESESYKNRMDSYQRKTGLTEAVQTG
TGQLNRIPVAIGIMDFQFMGGSMGSVVGEKITRLVEHAGNQLLPLILVCASGGARMQEGS
LSLMQMAKISSALYEYQKNKRLFYVSILTSPTTGRVTASFGMLGDIIIAEPDAYIAFAGKRVI
EQTLNTTIPEGSQVAEYLFQKGLFDSIVPRNPLKGVLSELFQLHAFFPL
>AhACC20
MFNSILFYRQLEYRCGLSNSMDSFSPIENTSASEDPILIDMKKDFPSWNDSDNSSYSNVDYL

VGVRNIRNFLFNKILLVRDNNSQRNRYCIYFDIENQFLEISNDPSFLSKPEYFFDSYNKNSSYLN
NVSKRHENHYMYDTKSSWKNGIHNCIESYLHCQICISHLGENDKYNDSYFYTSIYGKG
VNSSESEGSSIKTTITNENLTKREDSKDLDETKKYKHLWIECKNYYGLNYRKFFKSKMNIC
EHCGYHLKMSSSDRIEFSIDPCTWNPMDEDMVSMDPIEFHSEEEFKSIRIAGLTEAIQTGTD
QLNGIPVAIGIMDFQFMGGSMGSVVGEKITCLVEHAGNQLLPLQEGSLSLMQMAKISSAL
YEYQKNKRLFYVSILTSSTTGGATASFGMLGDTIIAEPDAYIAFAVNKNRMSRIHKKYLSKK
EMCKRNKEVRKKQTIPHTGGSKPISRKRHEI

>AhACC21

MTILDNNSQRNRYSYILILKNCSYLNKFLRDIIIIYTEIVVKVRVLPVKTTITNKNLTKRENS
KDLNETKKYKYLWIECKNYCGLNYRKFLR

>AhACC22

MVTQHFLWCEHCEYHLKKISTWNPMEEDMVSADPIEFHSEEQFYKKMYGLLLKRIGLTE
FMEDSMGSVLVCASERAHMQEGSLSI

>AhACC23

YLEYKCGLNNLIDYFDPVENTSASEDPILFVDVKNIKKILSNKILLIKDNNSQRNRYYIDIES
RNSSKSESSSIKTIITNENLTKRENSKDLNETKTYKHDD

>AhACC24

MCAGGKGAAGSSGNAISGAPTIAPLPPPQQGDHDAAANDNKDYRDLIMDFQFMEDSMRS
VVVCASKRAYMQEGSLSMTLFYVSILISLTTGRVTASFTMFGYIVDLIITLHLR

>AhACC25

MRNSDRIELSIDPGTWNPMDEDMVSMDPIEFHSEEESESCAAVAAESSTGSPLLTQKKSLLP
SSLQLCPEG

>AhACC26

MDSFGPVENTSASEDPILIDMEKDFPSWNDSDNSSYSNVDYLVGVRNIRNFLSDKILLVRD
NNSQRNRYSIYFDIENQFLEISNDPSFLSEPESLFDSYNKNSSYLNNVSKRHENHYMAEIVV
KARVLP

>AhACC27

MSLNSNFVHTIESVLGVSLGFHLGFGGPDYDDLGSTDIWDANLLEEIVEPKKGDETGHSA
EAVGKLKTLHAAKGMLTATTSNRIFFFFAADFKSGSFSTRAERLVALSGKGAIMTDRSLTSS
RDVTALFLRSNLKISGANNAGFGEIDLERQYVKGVVFLGPPAVSMAALGDKIGSPLIAQAA
EVPTLSWNGSHIRSSGVAFIISITATAFPFPFAEASTVMLLLASLATSPLKIGDGGGAPSISSFL
CLFSPGAIAAA

>AhACC28

MASSFASAASTTPSAIISPNYTYKCSSSSSCMLSFHLSHKTKLPLFTKVPPPSRIVLPRVKVE
PDDVSIFHWPSIENALSEAIYHLLNKLLGSSFVSKITTIDSSSNATVDNKTIAEVARSVDNDA
KTRSSEGVLATEKSISNFITHVASLVKLVDSRDIVELQLKKLDCEVIIRKQEAMPQPQAPTEV
AMLNLPPHTFATTGSVTYPTCSNLPGESMIALE

>AhACC29

MASSFASAASTTPSAIISPNYICKCSSSSSCMLSFHLSHKTMLPLFTKVPPPSRIVLPRVKVEP
DDVSIFHWPSIENALSEALYHLLNKLLGSIFVSKITTIDSSSNATVDNKTISEVARSVDNDAK
TRSSEGVLATEESISNFITHVASLVKLVDSRHIVELQLKKLDCEVIIRKQEAMPQPQAPTEVA
MLNCHHTPSPPPVV
