# Supplementary material for: Bioinformatics Identification and Expression Analysis of Acetyl-CoA Carboxylase Reveal Its Role in Isoflavone Accumulation during Soybean Seed Development
Source: Int J Mol Sci. 2024 Sep 23;25(18):10221. doi: 10.3390/ijms251810221 (PMC11432495; doi:10.3390/ijms251810221)
Supplement: Supplementary file 1 [file ijms-25-10221-s001.zip › ijms-3171334-supplementary/ijms-3171334-supplementary.pdf]

Table S1. Soybean Acetyl-CoA carboxylase (ACCase) gene family and functional annotation.

Gene Name	Gene ID	Chromosome	Length（aa）	MW	pI	Subcellular Localization Prediction	Group	Gene Description
GmACC1	Glyma.04G104900	4	2260	252370.45	6	Cytoplasmic	I	acetyl-CoA carboxylase 1
GmACC2	Glyma.06G105900	6	2260	252169.99	5.92	Cytoplasmic	I	acetyl-CoA carboxylase 1
GmACC3	Glyma.13G363500	13	298	32437.37	8.49	PlasmaMembrane、Chloroplast	III	biotin carboxyl carrier protein of acetyl-CoA carboxylase
GmACC4	Glyma.15G010300	15	269	29161.57	8.81	Nuclear、Chloroplast、Mitochondrial	III	biotin carboxyl carrier protein of acetyl-CoA carboxylase
GmACC5	Glyma.18G243500	18	262	27657.07	9.47	Chloroplast	III	biotin carboxyl carrier protein of acetyl-CoA carboxylase
GmACC6	Glyma.18G265300	18	284	29764.27	8.45	Chloroplast	III	biotin carboxyl carrier protein of acetyl-CoA carboxylase
GmACC7	Glyma.05G221100	5	539	58888.72	7.22	Mitochondrial	I	biotin carboxylase subunit
GmACC8	Glyma.07G137400	7	107	11530.56	9.03	Mitochondrial	I	biotin carboxylase
GmACC9	Glyma.08G027600	8	539	58807.60	7.22	Mitochondrial	I	biotin carboxylase subunit
GmACC10	Glyma.18G195700	18	709	78666.60	7.64	Mitochondrial、Cytoplasmic	V	acetyl-CoA carboxylase carboxyl transferase subunit alpha
GmACC11	Glyma.18G195900	18	690	76918.92	8.63	Mitochondrial、Cytoplasmic	V	acetyl-CoA carboxylase carboxyl transferase subunit alpha
GmACC12	Glyma.18G196000	18	683	75989.91	8.88	Cytoplasmic、Mitochondrial	V	acetyl-CoA carboxylase carboxyl transferase subunit alpha
GmACC13	Glyma.10G208900	10	34	3865.83	10.30	Mitochondrial、Chloroplast、Nuclear	IV	acetyl-CoA carboxylase carboxyl transferase subunit beta
GmACC14	Glyma.15G003800	15	34	3847.76	10.00	Mitochondrial、Chloroplast	IV	acetyl-CoA carboxylase carboxyl transferase subunit beta
GmACC15	Glyma.15G248500	15	126	14897.90	4.51	Nuclear	II	acetyl-coenzyme a carboxylase carboxyl transferase subunit beta, chloroplastic
GmACC16	Glyma.09G248900	9	261	27536.94	9.37	Chloroplast	III	biotin carboxyl carrier protein of acetyl-coa carboxylase 1, chloroplastic
GmACC17	Glyma.13G057400	13	276	28870.48	8.69	Chloroplast	III	biotin carboxyl carrier protein of acetyl-CoA carboxylase 2, chloroplastic
GmACC18	Glyma.11G233700	11	297	32064.75	9.01	Mitochondrial	III	biotin carboxyl carrier protein of acetyl-CoA carboxylase-like
GmACC19	Glyma.18G023300	18	291	31540.09	9.10	Mitochondrial	III	biotin carboxyl carrier protein of acetyl-CoA carboxylase-like
GmACC20	Glyma.19G028800	19	280	29326.88	8.16	Chloroplast	III	biotin carboxyl carrier protein of acetyl-CoA carboxylase 2, chloroplastic-like

Table S2. Members of the *ACC* gene family in various species

Gene Name	Gene ID	Organism	Gene Description	Group
AtACC1	AT1G36160	A.thaliana Araport11	acetyl-CoA carboxylase 1	I
AtACC2	AT1G36180	A.thaliana Araport11	acetyl-CoA carboxylase 2	I
AtACC3	AT5G16390	A.thaliana Araport11	biotin carboxyl carrier protein	III
AtACC4	AT5G15530	A.thaliana Araport11	biotin carboxyl carrier protein 2	III
AtACC5	AT5G35360	A.thaliana Araport11	acetyl Co-enzyme a carboxylase biotin carboxylase subunit	I
AtACC6	AT2G38040	A.thaliana Araport11	acetyl Co-enzyme a carboxylase carboxyltransferase alpha subunit	V
AtACC7	ATCG00500	A.thaliana Araport11	acetyl-CoA carboxylase carboxyl transferase subunit beta	VI
AtACC8	AT5G16390	A.thaliana Araport11	chloroplastic acetylcoenzyme A carboxylase 1	III
AhACC1	arahy.Tifrunner.gnm1.ann1.ZST20A	A.hypogaea v1.0	acetyl-CoA carboxylase / biotin carboxylase (ACAC)	I
AhACC2	arahy.Tifrunner.gnm1.ann1.L14VXE	A.hypogaea v1.0	acetyl-CoA carboxylase / biotin carboxylase	I
AhACC3	arahy.Tifrunner.gnm1.ann1.U9Q8NZ	A.hypogaea v1.0	acetyl-CoA carboxylase biotin carboxyl carrier protein (accB, bccP)	III
AhACC4	arahy.Tifrunner.gnm1.ann1.4YN66T	A.hypogaea v1.0	acetyl-CoA carboxylase, biotin carboxylase subunit (accC)	I
AhACC5	arahy.Tifrunner.gnm1.ann1.KBA53Y	A.hypogaea v1.0	acetyl-CoA carboxylase biotin carboxyl carrier protein (accB, bccP)	III
AhACC6	arahy.Tifrunner.gnm1.ann1.S0JXUX	A.hypogaea v1.0	acetyl-CoA carboxylase biotin carboxyl carrier protein (accB, bccP)	III
AhACC7	arahy.Tifrunner.gnm1.ann1.H4YX61	A.hypogaea v1.0	acetyl-CoA carboxylase biotin carboxyl carrier protein (accB, bccP)	III
AhACC8	arahy.Tifrunner.gnm1.ann1.58VMN1	A.hypogaea v1.0	acetyl-CoA carboxylase, biotin carboxylase subunit (accC)	I
AhACC9	arahy.Tifrunner.gnm1.ann1.H4HC75	A.hypogaea v1.0	acetyl-CoA carboxylase, biotin carboxylase subunit (accC)	I
AhACC10	arahy.Tifrunner.gnm1.ann1.KH93XI	A.hypogaea v1.0	acetyl-CoA carboxylase biotin carboxyl carrier protein (accB, bccP)	III
AhACC11	arahy.Tifrunner.gnm1.ann1.7FW3W7	A.hypogaea v1.0	acetyl-CoA carboxylase, biotin carboxylase subunit (accC)	I
AhACC12	arahy.Tifrunner.gnm1.ann1.E1R28C	A.hypogaea v1.0	acetyl-CoA carboxylase, biotin carboxylase subunit (accC)	I
AhACC13	arahy.Tifrunner.gnm1.ann1.AG8095	A.hypogaea v1.0	acetyl-CoA carboxylase biotin carboxyl carrier protein (accB, bccP)	III
AhACC14	arahy.Tifrunner.gnm1.ann1.1S754L	A.hypogaea v1.0	acetyl-CoA carboxylase, biotin carboxylase subunit (accC)	I
AhACC15	arahy.Tifrunner.gnm1.ann1.JYC8LX	A.hypogaea v1.0	acetyl-CoA carboxylase carboxyl transferase subunit alpha (accA)	V

AhACC16	arahy.Tifrunner.gnm1.ann1.70V125	A.hypogaea v1.0	acetyl-CoA carboxylase carboxyl transferase subunit alpha (accA)	V
AhACC17	arahy.Tifrunner.gnm1.ann1.45ZUWH	A.hypogaea v1.0	acetyl-CoA carboxylase carboxyl transferase subunit alpha (accA)	V
AhACC18	arahy.Tifrunner.gnm1.ann1.3BKK3V	A.hypogaea v1.0	acetyl-CoA carboxylase carboxyl transferase subunit alpha (accA)	V
AhACC19	arahy.Tifrunner.gnm1.ann1.15M9SP	A.hypogaea v1.0	acetyl-CoA carboxylase carboxyl transferase subunit beta (accD)	VI
AhACC20	arahy.Tifrunner.gnm1.ann1.J4M97S	A.hypogaea v1.0	acetyl-CoA carboxylase carboxyl transferase subunit beta (accD)	II
AhACC21	arahy.Tifrunner.gnm1.ann1.MXT580	A.hypogaea v1.0	Acetyl-CoA carboxylase	II
AhACC22	arahy.Tifrunner.gnm1.ann1.YEGK0N	A.hypogaea v1.0	Acetyl-CoA carboxylase	VI
AhACC23	arahy.Tifrunner.gnm1.ann1.48ICNX	A.hypogaea v1.0	Acetyl-CoA carboxylase	II
AhACC24	arahy.Tifrunner.gnm1.ann1.E6F9B2	A.hypogaea v1.0	Acetyl-CoA carboxylase	VII
AhACC25	arahy.Tifrunner.gnm1.ann1.6V23H5	A.hypogaea v1.0	Acetyl-CoA carboxylase	VI
AhACC26	arahy.Tifrunner.gnm1.ann1.3WU3U6	A.hypogaea v1.0	Acetyl-CoA carboxylase	II
AhACC27	arahy.Tifrunner.gnm1.ann1.R8EQGM	A.hypogaea v1.0	acetyl-CoA carboxylase 1	VII
AhACC28	arahy.Tifrunner.gnm1.ann1.RG375V	A.hypogaea v1.0	biotin carboxyl carrier protein of acetyl-coa carboxylase 1, chloroplastic	III
AhACC29	arahy.Tifrunner.gnm1.ann1.4N3F0D	A.hypogaea v1.0	biotin carboxyl carrier protein of acetyl-coa carboxylase 1, chloroplastic	III
OsACC1	OsKitaake10g071200	O.sativaKitaake v3.1	biotin carboxylase	I
OsACC2	OsKitaake05g101700	O.sativaKitaake v3.1	biotin carboxylase	I
OsACC3	OsKitaake10g067355	O.sativaKitaake v3.1	Acetyl-CoA carboxylase	V
OsACC4	OsKitaake01g359300	O.sativaKitaake v3.1	Acetyl-CoA carboxylase	V
OsACC5	OsKitaake12g099500	O.sativaKitaake v3.1	Acetyl-CoA carboxylase	V
ZmACC1	Zm00001eb419400	Zea mays Zm-B73-REFERENCE-NAM-5.0.55	acetyl-CoA carboxylase / biotin carboxylase (ACAC)	I
ZmACC2	Zm00001eb086560	Zea mays Zm-B73-REFERENCE-NAM-5.0.55	acetyl-CoA carboxylase / biotin carboxylase (ACAC)	I
ZmACC3	Zm00001eb223980	Zea mays Zm-B73-REFERENCE-NAM-5.0.55	acetyl-CoA carboxylase / biotin carboxylase (ACAC)	I
ZmACC4	Zm00001eb367400	Zea mays Zm-B73-REFERENCE-NAM-5.0.55	Acetyl-CoA carboxylase	I
ZmACC5	Zm00001eb189990	Zea mays Zm-B73-REFERENCE-NAM-5.0.55	Acetyl-CoA carboxylase	I
ZmACC6	Zm00001eb428980	Zea mays Zm-B73-REFERENCE-NAM-5.0.55	biotin carboxylase // Acetyl-CoA carboxylase	I
ZmACC7	Zm00001eb028920	Zea mays Zm-B73-REFERENCE-NAM-5.0.55	Acetyl-CoA carboxylase	I

Table S3. Cis-acting elements in promoter sequences of *GmACCs*

Gene Name	Position	Function
GmACC1	39	cis-acting element involved in the abscisic acid responsiveness
GmACC1	30	cis-acting element involved in the abscisic acid responsiveness
GmACC1	646	cis-acting element involved in the abscisic acid responsiveness
GmACC1	691	cis-acting element involved in the abscisic acid responsiveness
GmACC1	868	cis-acting element involved in the abscisic acid responsiveness
GmACC1	869	cis-acting element involved in the abscisic acid responsiveness
GmACC1	939	cis-acting regulatory element essential for the anaerobic induction
GmACC1	976	cis-acting regulatory element essential for the anaerobic induction
GmACC1	981	cis-acting regulatory element essential for the anaerobic induction
GmACC1	1352	cis-acting regulatory element essential for the anaerobic induction
GmACC1	1358	cis-acting regulatory element essential for the anaerobic induction
GmACC1	689	cis-acting regulatory element involved in the MeJA-responsiveness
GmACC1	1140	cis-acting regulatory element involved in the MeJA-responsiveness
GmACC1	689	cis-acting regulatory element involved in the MeJA-responsiveness
GmACC1	1140	cis-acting regulatory element involved in the MeJA-responsiveness
GmACC1	327	gibberellin-responsive element
GmACC1	971	gibberellin-responsive element
GmACC1	1337	gibberellin-responsive element
GmACC2	1508	cis-acting element involved in low-temperature responsiveness
GmACC2	686	cis-acting element involved in the abscisic acid responsiveness
GmACC2	870	cis-acting element involved in the abscisic acid responsiveness
GmACC2	871	cis-acting element involved in the abscisic acid responsiveness
GmACC2	976	cis-acting regulatory element essential for the anaerobic induction
GmACC2	981	cis-acting regulatory element essential for the anaerobic induction
GmACC2	1367	cis-acting regulatory element essential for the anaerobic induction
GmACC2	684	cis-acting regulatory element involved in the MeJA-responsiveness
GmACC2	684	cis-acting regulatory element involved in the MeJA-responsiveness
GmACC2	971	gibberellin-responsive element
GmACC2	1297	gibberellin-responsive element
GmACC2	1352	gibberellin-responsive element
GmACC3	1989	cis-acting element involved in defense and stress responsiveness
GmACC3	391	cis-acting element involved in salicylic acid responsiveness
GmACC3	1395	cis-acting element involved in the abscisic acid responsiveness
GmACC3	1970	cis-acting element involved in the abscisic acid responsiveness
GmACC3	185	cis-acting regulatory element related to meristem expression
GmACC3	1807	gibberellin-responsive element
GmACC3	667	MYB binding site involved in light responsiveness
GmACC4	1910	cis-acting element involved in defense and stress responsiveness
GmACC4	762	cis-acting element involved in gibberellin-responsiveness
GmACC4	850	cis-acting element involved in gibberellin-responsiveness

GmACC4	718	cis-acting element involved in salicylic acid responsiveness
GmACC4	1965	cis-acting element involved in salicylic acid responsiveness
GmACC4	1410	cis-acting regulatory element involved in seed-specific regulation
GmACC4	940	element for maximal elicitor-mediated activation (2copies)
GmACC5	591	cis-acting element involved in defense and stress responsiveness
GmACC5	302	cis-acting element involved in the abscisic acid responsiveness
GmACC5	916	cis-acting element involved in the abscisic acid responsiveness
GmACC5	1844	cis-acting element involved in the abscisic acid responsiveness
GmACC5	1845	cis-acting element involved in the abscisic acid responsiveness
GmACC5	989	cis-acting regulatory element essential for the anaerobic induction
GmACC5	1866	cis-acting regulatory element related to meristem expression
GmACC6	338	cis-acting element involved in the abscisic acid responsiveness
GmACC6	1385	cis-acting element involved in the abscisic acid responsiveness
GmACC6	72	cis-acting regulatory element essential for the anaerobic induction
GmACC6	1768	cis-acting regulatory element essential for the anaerobic induction
GmACC6	1725	cis-acting regulatory element involved in the MeJA-responsiveness
GmACC6	1848	cis-acting regulatory element involved in the MeJA-responsiveness
GmACC6	1725	cis-acting regulatory element involved in the MeJA-responsiveness
GmACC6	1848	cis-acting regulatory element involved in the MeJA-responsiveness
GmACC7	1414	cis-acting element involved in defense and stress responsiveness
GmACC7	389	cis-acting element involved in low-temperature responsiveness
GmACC7	407	cis-acting element involved in low-temperature responsiveness
GmACC7	1691	cis-acting element involved in salicylic acid responsiveness
GmACC7	1529	cis-acting element involved in the abscisic acid responsiveness
GmACC7	1829	cis-acting element involved in the abscisic acid responsiveness
GmACC7	568	cis-acting regulatory element essential for the anaerobic induction
GmACC7	988	cis-acting regulatory element involved in the MeJA-responsiveness
GmACC7	988	cis-acting regulatory element involved in the MeJA-responsiveness
GmACC7	659	element involved in differentiation of the palisade mesophyll cells
GmACC7	974	gibberellin-responsive element
GmACC7	1998	gibberellin-responsive element
GmACC7	1667	MYB binding site involved in light responsiveness
GmACC7	988	auxin-responsive element
GmACC8	288	auxin-responsive element
GmACC8	1451	cis-acting element involved in salicylic acid responsiveness
GmACC8	1583	cis-acting element involved in salicylic acid responsiveness
GmACC8	1905	cis-acting element involved in salicylic acid responsiveness
GmACC8	330	cis-acting regulatory element essential for the anaerobic induction
GmACC8	575	cis-acting regulatory element essential for the anaerobic induction
GmACC8	1030	cis-acting regulatory element essential for the anaerobic induction
GmACC8	1482	cis-acting regulatory element essential for the anaerobic induction
GmACC8	1702	gibberellin-responsive element
GmACC8	1877	gibberellin-responsive element
GmACC8	1740	MYB binding site involved in drought-inducibility

GmACC8	1756	MYB binding site involved in drought-inducibility
GmACC9	375	binding site of AT-rich DNA binding protein (ATBP-1)
GmACC9	999	cis-acting element involved in defense and stress responsiveness
GmACC9	1211	cis-acting element involved in defense and stress responsiveness
GmACC9	134	cis-acting element involved in low-temperature responsiveness
GmACC9	424	cis-acting element involved in the abscisic acid responsiveness
GmACC9	1395	cis-acting regulatory element essential for the anaerobic induction
GmACC9	828	cis-acting regulatory element related to meristem expression
GmACC9	356	element involved in differentiation of the palisade mesophyll cells
GmACC10	1249	cis-acting element involved in defense and stress responsiveness
GmACC10	1544	cis-acting element involved in defense and stress responsiveness
GmACC10	595	cis-acting element involved in gibberellin-responsiveness
GmACC10	383	cis-acting element involved in the abscisic acid responsiveness
GmACC10	1710	cis-acting regulatory element essential for the anaerobic induction
GmACC10	1811	cis-acting regulatory element involved in the MeJA-responsiveness
GmACC10	1811	cis-acting regulatory element involved in the MeJA-responsiveness
GmACC10	541	gibberellin-responsive element
GmACC10	67	gibberellin-responsive element
GmACC10	1635	gibberellin-responsive element
GmACC11	1517	cis-acting element involved in cell cycle regulation
GmACC11	1167	cis-acting element involved in defense and stress responsiveness
GmACC11	1245	cis-acting element involved in low-temperature responsiveness
GmACC11	1249	cis-acting element involved in salicylic acid responsiveness
GmACC11	87	cis-acting element involved in the abscisic acid responsiveness
GmACC11	1919	cis-acting regulatory element essential for the anaerobic induction
GmACC11	684	cis-acting regulatory element involved in the MeJA-responsiveness
GmACC11	1445	cis-acting regulatory element involved in the MeJA-responsiveness
GmACC11	1791	cis-acting regulatory element involved in the MeJA-responsiveness
GmACC11	684	cis-acting regulatory element involved in the MeJA-responsiveness
GmACC11	1445	cis-acting regulatory element involved in the MeJA-responsiveness
GmACC11	1791	cis-acting regulatory element involved in the MeJA-responsiveness
GmACC11	1102	gibberellin-responsive element
GmACC11	1615	gibberellin-responsive element
GmACC11	943	MYB binding site involved in drought-inducibility
GmACC12	644	auxin-responsive element
GmACC12	1521	cis-acting element involved in cell cycle regulation
GmACC12	1168	cis-acting element involved in defense and stress responsiveness
GmACC12	1242	cis-acting element involved in salicylic acid responsiveness
GmACC12	1558	cis-acting element involved in salicylic acid responsiveness
GmACC12	34	cis-acting element involved in the abscisic acid responsiveness
GmACC12	787	cis-acting regulatory element essential for the anaerobic induction
GmACC12	1235	cis-acting regulatory element essential for the anaerobic induction
GmACC12	1692	cis-acting regulatory element essential for the anaerobic induction
GmACC12	1919	cis-acting regulatory element essential for the anaerobic induction

GmACC12	1793	cis-acting regulatory element involved in the MeJA-responsiveness
GmACC12	1793	cis-acting regulatory element involved in the MeJA-responsiveness
GmACC12	649	gibberellin-responsive element
GmACC12	1072	gibberellin-responsive element
GmACC12	1617	gibberellin-responsive element
GmACC13	411	cis-acting element involved in gibberellin-responsiveness
GmACC13	1678	cis-acting element involved in gibberellin-responsiveness
GmACC13	1898	cis-acting element involved in salicylic acid responsiveness
GmACC13	630	cis-acting element involved in the abscisic acid responsiveness
GmACC13	950	cis-acting element involved in the abscisic acid responsiveness
GmACC13	867	cis-acting regulatory element essential for the anaerobic induction
GmACC13	779	cis-acting regulatory element involved in the MeJA-responsiveness
GmACC13	779	cis-acting regulatory element involved in the MeJA-responsiveness
GmACC13	831	cis-acting regulatory element related to meristem expression
GmACC13	808	MYB binding site involved in drought-inducibility
GmACC13	1895	MYB binding site involved in flavonoid biosynthetic genes regulation
GmACC13	1149	MYB binding site involved in light responsiveness
GmACC14	1023	cis-acting element involved in salicylic acid responsiveness
GmACC14	674	cis-acting element involved in the abscisic acid responsiveness
GmACC14	408	cis-acting regulatory element essential for the anaerobic induction
GmACC14	1577	cis-acting regulatory element essential for the anaerobic induction
GmACC14	1990	element involved in differentiation of the palisade mesophyll cells
GmACC15	426	binding site of AT-rich DNA binding protein (ATBP-1)
GmACC15	1857	binding site of AT-rich DNA binding protein (ATBP-1)
GmACC15	1220	cis-acting element involved in gibberellin-responsiveness
GmACC15	667	cis-acting element involved in salicylic acid responsiveness
GmACC15	360	cis-acting element involved in the abscisic acid responsiveness
GmACC15	1997	cis-acting regulatory element essential for the anaerobic induction
GmACC15	502	MYB binding site involved in drought-inducibility
GmACC16	907	cis-acting element involved in salicylic acid responsiveness
GmACC16	647	cis-acting element involved in the abscisic acid responsiveness
GmACC16	756	cis-acting element involved in the abscisic acid responsiveness
GmACC16	1181	cis-acting element involved in the abscisic acid responsiveness
GmACC16	1183	cis-acting element involved in the abscisic acid responsiveness
GmACC16	1920	cis-acting element involved in the abscisic acid responsiveness
GmACC16	1921	cis-acting element involved in the abscisic acid responsiveness
GmACC16	219	cis-acting regulatory element essential for the anaerobic induction
GmACC16	224	cis-acting regulatory element essential for the anaerobic induction
GmACC16	1015	cis-acting regulatory element essential for the anaerobic induction
GmACC16	551	cis-acting regulatory element involved in circadian control
GmACC16	1925	cis-acting regulatory element related to meristem expression
GmACC16	60	gibberellin-responsive element
GmACC16	106	gibberellin-responsive element
GmACC16	419	gibberellin-responsive element

GmACC17	1834	cis-acting element involved in low-temperature responsiveness
GmACC17	15	cis-acting element involved in salicylic acid responsiveness
GmACC17	1068	cis-acting element involved in the abscisic acid responsiveness
GmACC17	1388	cis-acting element involved in the abscisic acid responsiveness
GmACC17	96	cis-acting regulatory element essential for the anaerobic induction
GmACC17	344	cis-acting regulatory element essential for the anaerobic induction
GmACC17	886	cis-acting regulatory element essential for the anaerobic induction
GmACC17	1640	cis-acting regulatory element essential for the anaerobic induction
GmACC17	1749	cis-acting regulatory element essential for the anaerobic induction
GmACC17	1754	cis-acting regulatory element essential for the anaerobic induction
GmACC17	1759	cis-acting regulatory element essential for the anaerobic induction
GmACC17	1998	cis-acting regulatory element essential for the anaerobic induction
GmACC17	1501	cis-acting regulatory element related to meristem expression
GmACC17	769	MYB binding site involved in drought-inducibility
GmACC18	706	cis-acting element involved in the abscisic acid responsiveness
GmACC18	972	cis-acting element involved in the abscisic acid responsiveness
GmACC18	1012	cis-acting element involved in the abscisic acid responsiveness
GmACC18	1295	cis-acting element involved in the abscisic acid responsiveness
GmACC18	1459	cis-acting element involved in the abscisic acid responsiveness
GmACC18	1698	cis-acting element involved in the abscisic acid responsiveness
GmACC18	1701	cis-acting element involved in the abscisic acid responsiveness
GmACC18	38	cis-acting regulatory element essential for the anaerobic induction
GmACC18	262	cis-acting regulatory element essential for the anaerobic induction
GmACC18	534	cis-acting regulatory element involved in the MeJA-responsiveness
GmACC18	598	cis-acting regulatory element involved in the MeJA-responsiveness
GmACC18	636	cis-acting regulatory element involved in the MeJA-responsiveness
GmACC18	691	cis-acting regulatory element involved in the MeJA-responsiveness
GmACC18	534	cis-acting regulatory element involved in the MeJA-responsiveness
GmACC18	598	cis-acting regulatory element involved in the MeJA-responsiveness
GmACC18	636	cis-acting regulatory element involved in the MeJA-responsiveness
GmACC18	691	cis-acting regulatory element involved in the MeJA-responsiveness
GmACC18	526	MYB binding site involved in light responsiveness
GmACC19	1274	cis-acting element involved in defense and stress responsiveness
GmACC19	1526	cis-acting element involved in defense and stress responsiveness
GmACC19	35	cis-acting element involved in salicylic acid responsiveness
GmACC19	882	cis-acting element involved in salicylic acid responsiveness
GmACC19	741	cis-acting element involved in the abscisic acid responsiveness
GmACC19	1760	cis-acting element involved in the abscisic acid responsiveness
GmACC19	1809	cis-acting element involved in the abscisic acid responsiveness
GmACC19	341	cis-acting regulatory element essential for the anaerobic induction
GmACC19	1209	cis-acting regulatory element essential for the anaerobic induction
GmACC19	1249	cis-acting regulatory element essential for the anaerobic induction
GmACC19	622	cis-acting regulatory element involved in circadian control
GmACC19	393	cis-acting regulatory element involved in the MeJA-responsiveness

GmACC19	393	cis-acting regulatory element involved in the MeJA-responsiveness
GmACC19	370	MYB binding site involved in drought-inducibility
GmACC19	393	auxin-responsive element
GmACC20	1260	auxin-responsive element
GmACC20	1585	binding site of AT-rich DNA binding protein (ATBP-1)
GmACC20	1819	cis-acting element involved in defense and stress responsiveness
GmACC20	60	cis-acting element involved in gibberellin-responsiveness
GmACC20	1833	cis-acting element involved in low-temperature responsiveness
GmACC20	521	cis-acting regulatory element essential for the anaerobic induction
GmACC20	1029	cis-acting regulatory element essential for the anaerobic induction
GmACC20	1764	cis-acting regulatory element essential for the anaerobic induction
GmACC20	1769	cis-acting regulatory element essential for the anaerobic induction
GmACC20	1998	cis-acting regulatory element essential for the anaerobic induction
GmACC20	720	cis-acting regulatory element involved in the MeJA-responsiveness
GmACC20	720	cis-acting regulatory element involved in the MeJA-responsiveness
GmACC20	1477	cis-acting regulatory element related to meristem expression
GmACC20	855	MYB binding site involved in light responsiveness

Table S4. Orthologous gene pairs between soybean and other plant species.

No.	Gene name	Gene ID of Soybean	Syntenic with	Organism	Gene ID
1	GmACC1	Glyma.04G104900	——	Arabidopsis thaliana Araport11	AT1G36160
2	GmACC2	Glyma.06G105900	——	Arabidopsis thaliana Araport11	AT1G36160
3	GmACC3	Glyma.13G363500	——	Arabidopsis thaliana Araport11	AT1G52670
4	GmACC4	Glyma.13G363500	——	Arabidopsis thaliana Araport11	AT3G15690
5	GmACC5	Glyma.15G010300	——	Arabidopsis thaliana Araport11	AT1G52670
6	GmACC5	Glyma.15G010300	——	Arabidopsis thaliana Araport11	AT3G15690
7	GmACC6	Glyma.18G243500	——	Arabidopsis thaliana Araport11	AT5G16390
8	GmACC6	Glyma.18G265300	——	Arabidopsis thaliana Araport11	AT5G15530
9	GmACC10	Glyma.18G195700	——	Arabidopsis thaliana Araport11	AT2G38040
10	GmACC16	Glyma.09G248900	——	Arabidopsis thaliana Araport11	AT5G16390
11	GmACC17	Glyma.13G057400	——	Arabidopsis thaliana Araport11	AT5G15530
12	GmACC18	Glyma.11G233700	——	Arabidopsis thaliana Araport11	AT3G56130
13	GmACC19	Glyma.18G023300	——	Arabidopsis thaliana Araport11	AT3G56130
14	GmACC20	Glyma.19G028800	——	Arabidopsis thaliana Araport11	AT5G15530
15	GmACC1	Glyma.04G104900	——	A.hypogaea v1.0	arahy.Tifrunner.gnm1.ann1.L14VXE
16	GmACC1	Glyma.04G104900	——	A.hypogaea v1.0	arahy.Tifrunner.gnm1.ann1.ZST20A
17	GmACC2	Glyma.06G105900	——	A.hypogaea v1.0	arahy.Tifrunner.gnm1.ann1.L14VXE
18	GmACC2	Glyma.06G105900	——	A.hypogaea v1.0	arahy.Tifrunner.gnm1.ann1.ZST20A
19	GmACC3	Glyma.13G363500	——	A.hypogaea v1.0	arahy.Tifrunner.gnm1.ann1.XUD8XD
20	GmACC4	Glyma.15G010300	——	A.hypogaea v1.0	arahy.Tifrunner.gnm1.ann1.XUD8XD
21	GmACC5	Glyma.18G243500	——	A.hypogaea v1.0	arahy.Tifrunner.gnm1.ann1.U9Q8NZ
22	GmACC5	Glyma.18G243500	——	A.hypogaea v1.0	arahy.Tifrunner.gnm1.ann1.RG375V
23	GmACC5	Glyma.18G243500	——	A.hypogaea v1.0	arahy.Tifrunner.gnm1.ann1.KH93XI

24	GmACC5	Glyma.18G243500	——	A.hypogaea v1.0	arahy.Tifrunner.gnm1.ann1.4N3F0D
25	GmACC6	Glyma.18G265300	——	A.hypogaea v1.0	arahy.Tifrunner.gnm1.ann1.KBA53Y
26	GmACC6	Glyma.18G265300	——	A.hypogaea v1.0	arahy.Tifrunner.gnm1.ann1.AG8095
27	GmACC6	Glyma.18G265300	——	A.hypogaea v1.0	arahy.Tifrunner.gnm1.ann1.H4YX61
28	GmACC6	Glyma.18G265300	——	A.hypogaea v1.0	arahy.Tifrunner.gnm1.ann1.S0JXUX
29	GmACC7	Glyma.05G221100	——	A.hypogaea v1.0	arahy.Tifrunner.gnm1.ann1.E1R28C
30	GmACC7	Glyma.05G221100	——	A.hypogaea v1.0	arahy.Tifrunner.gnm1.ann1.1S754L
31	GmACC7	Glyma.05G221100	——	A.hypogaea v1.0	arahy.Tifrunner.gnm1.ann1.H4HC75
32	GmACC7	Glyma.05G221100	——	A.hypogaea v1.0	arahy.Tifrunner.gnm1.ann1.7FW3W7
33	GmACC9	Glyma.08G027600	——	A.hypogaea v1.0	arahy.Tifrunner.gnm1.ann1.E1R28C
34	GmACC9	Glyma.08G027600	——	A.hypogaea v1.0	arahy.Tifrunner.gnm1.ann1.1S754L
35	GmACC9	Glyma.08G027600	——	A.hypogaea v1.0	arahy.Tifrunner.gnm1.ann1.H4HC75
36	GmACC9	Glyma.08G027600	——	A.hypogaea v1.0	arahy.Tifrunner.gnm1.ann1.7FW3W7
37	GmACC10	Glyma.18G195700	——	A.hypogaea v1.0	arahy.Tifrunner.gnm1.ann1.45ZUWH
38	GmACC10	Glyma.18G195700	——	A.hypogaea v1.0	arahy.Tifrunner.gnm1.ann1.70V125
39	GmACC16	Glyma.09G248900	——	A.hypogaea v1.0	arahy.Tifrunner.gnm1.ann1.U9Q8NZ
40	GmACC16	Glyma.09G248900	——	A.hypogaea v1.0	arahy.Tifrunner.gnm1.ann1.RG375V
41	GmACC16	Glyma.09G248900	——	A.hypogaea v1.0	arahy.Tifrunner.gnm1.ann1.KH93XI
42	GmACC16	Glyma.09G248900	——	A.hypogaea v1.0	arahy.Tifrunner.gnm1.ann1.4N3F0D
43	GmACC17	Glyma.13G057400	——	A.hypogaea v1.0	arahy.Tifrunner.gnm1.ann1.KBA53Y
44	GmACC17	Glyma.13G057400	——	A.hypogaea v1.0	arahy.Tifrunner.gnm1.ann1.AG8095
45	GmACC17	Glyma.13G057400	——	A.hypogaea v1.0	arahy.Tifrunner.gnm1.ann1.H4YX61
46	GmACC17	Glyma.13G057400	——	A.hypogaea v1.0	arahy.Tifrunner.gnm1.ann1.S0JXUX
47	GmACC18	Glyma.11G233700	——	A.hypogaea v1.0	arahy.Tifrunner.gnm1.ann1.2XR7QU
48	GmACC18	Glyma.11G233700	——	A.hypogaea v1.0	arahy.Tifrunner.gnm1.ann1.JA22RM
49	GmACC18	Glyma.11G233700	——	A.hypogaea v1.0	arahy.Tifrunner.gnm1.ann1.E2TW3L

50	GmACC18	Glyma.11G233700	——	A.hypogaea v1.0	arahy.Tifrunner.gnm1.ann1.AGI60H
51	GmACC19	Glyma.18G023300	——	A.hypogaea v1.0	arahy.Tifrunner.gnm1.ann1.2XR7QU
52	GmACC19	Glyma.18G023300	——	A.hypogaea v1.0	arahy.Tifrunner.gnm1.ann1.JA22RM
53	GmACC19	Glyma.18G023300	——	A.hypogaea v1.0	arahy.Tifrunner.gnm1.ann1.E2TW3L
54	GmACC19	Glyma.18G023300	——	A.hypogaea v1.0	arahy.Tifrunner.gnm1.ann1.AGI60H
55	GmACC20	Glyma.19G028800	——	A.hypogaea v1.0	arahy.Tifrunner.gnm1.ann1.KBA53Y
56	GmACC20	Glyma.19G028800	——	A.hypogaea v1.0	arahy.Tifrunner.gnm1.ann1.AG8095
57	GmACC20	Glyma.19G028800	——	A.hypogaea v1.0	arahy.Tifrunner.gnm1.ann1.S0JXUX
58	GmACC20	Glyma.19G028800	——	A.hypogaea v1.0	arahy.Tifrunner.gnm1.ann1.H4YX61

Table S5. Isoflavone content in seed of different varieties

No.	Variety	Daidzein content (μg/g)	Glycitein content (μg/g)	Genistein content (μg/g)	Isoflavone content (μg/g)
1-1	Yuanbao Jin	1061.36	440.50	811.99	2313.86
1-2	Feng Shou No. 6	1088.08	347.26	1121.53	2556.87
1-3	Hong Feng No. 11	1049.03	399.11	1030.90	2479.04
1-4	Ken Feng 11	881.13	444.68	1068.56	2394.37
1-5	Jiangmo Bean No. 1	380.39	466.92	549.02	1396.33
1-6	Meng Dou 12	709.07	429.39	1046.71	2185.17
1-7	L-79	539.19	389.31	1204.73	2133.22
1-8	North 4834	767.27	655.88	1152.62	2575.78
1-9	Ji Yu 94	921.50	377.57	1040.84	2339.91
1-10	Yanqi Soybeans	714.63	460.50	808.20	1983.34
2-1	Sui Nong 10	1498.77	456.07	1725.61	3680.45
2-2	Sui Nong 29	1632.07	553.57	2256.36	4441.99
2-3	He 97-793	1127.26	454.30	2408.04	3989.60
2-4	Hei Nong 61	1233.04	519.72	1963.99	3716.75
2-5	Hei Nong 37	1516.53	499.96	1532.78	3549.27
2-6	Conrad	1446.31	584.45	1751.42	3782.18
2-7	Sui 03-3952	1739.53	474.16	2403.92	4617.60
2-8	Sui 04-5804	1786.88	452.05	1837.19	4076.11
2-9	Small-seeded Fodder Bean	1209.61	484.09	1883.63	3577.33
2-10	Zhong Dou 32	2248.73	632.89	1806.51	4688.14

Table S6. Spearman's Correlation between *GmACCs* and isoflavone accumulation

Var1	Var2	Cor	P_value	Significance
isoflavone accumulation	GmACC1	0.947	0.053	
isoflavone accumulation	GmACC2	0.984	0.016	*
isoflavone accumulation	GmACC3	0.983	0.017	*
isoflavone accumulation	GmACC4	0.829	0.171	
isoflavone accumulation	GmACC5	0.814	0.186	
isoflavone accumulation	GmACC6	0.834	0.166	
isoflavone accumulation	GmACC7	0.673	0.327	
isoflavone accumulation	GmACC9	0.833	0.167	
isoflavone accumulation	GmACC10	0.734	0.266	
isoflavone accumulation	GmACC11	0.813	0.187	
isoflavone accumulation	GmACC12	0.815	0.185	
isoflavone accumulation	GmACC13	-0.442	0.558	
isoflavone accumulation	GmACC14	-0.194	0.806	
isoflavone accumulation	GmACC15	0.992	0.008	**
isoflavone accumulation	GmACC16	0.445	0.555	
isoflavone accumulation	GmACC17	0.982	0.018	*
isoflavone accumulation	GmACC18	0.832	0.168	
isoflavone accumulation	GmACC19	0.800	0.200	
isoflavone accumulation	GmACC20	0.988	0.012	*

Note：*andsignificant at 0.05 and 0.01 levels**

Table S7. Genes in each module

Gene	ModuleColor
Glyma.01G131100	blue
Glyma.01G164300	blue
Glyma.02G020000	blue
Glyma.02G229700	blue
Glyma.03G158400	blue
Glyma.03G197600	blue
Glyma.04G199200	blue
Glyma.04G231500	blue
Glyma.05G091100	blue
Glyma.05G124500	blue
Glyma.05G149500	blue
Glyma.05G209400	blue
Glyma.05G221100	blue
Glyma.06G082400	blue
Glyma.06G133600	blue
Glyma.06G166300	blue
Glyma.06G275700	blue
Glyma.07G190600	blue
Glyma.07G240600	blue
Glyma.07G258000	blue
Glyma.08G016100	blue
Glyma.08G027600	blue
Glyma.08G103900	blue
Glyma.08G282800	blue
Glyma.08G311900	blue
Glyma.08G312300	blue
Glyma.10G049100	blue
Glyma.11G011300	blue
Glyma.11G020900	blue
Glyma.11G192900	blue
Glyma.11G233700	blue
Glyma.12G161500	blue
Glyma.13G056100	blue
Glyma.13G136900	blue
Glyma.13G179600	blue
Glyma.13G308100	blue
Glyma.14G053300	blue
Glyma.14G058400	blue
Glyma.14G111800	blue
Glyma.14G130700	blue
Glyma.14G183000	blue

Glyma.15G010300	blue
Glyma.16G039500	blue
Glyma.16G155800	blue
Glyma.17G023400	blue
Glyma.17G171100	blue
Glyma.17G202700	blue
Glyma.17G216000	blue
Glyma.18G195900	blue
Glyma.18G196000	blue
Glyma.18G243500	blue
Glyma.18G265300	blue
Glyma.19G028800	blue
Glyma.19G160800	blue
Glyma.19G195500	blue
Glyma.02G216000	brown
Glyma.04G004000	brown
Glyma.04G096900	brown
Glyma.05G133100	brown
Glyma.05G140200	brown
Glyma.05G140800	brown
Glyma.06G003600	brown
Glyma.06G098700	brown
Glyma.06G228700	brown
Glyma.07G046200	brown
Glyma.07G235800	brown
Glyma.08G079600	brown
Glyma.08G087600	brown
Glyma.10G096200	brown
Glyma.10G133700	brown
Glyma.11G107500	brown
Glyma.12G224200	brown
Glyma.12G236000	brown
Glyma.13G059400	brown
Glyma.13G201500	brown
Glyma.14G196800	brown
Glyma.15G035100	brown
Glyma.15G093600	brown
Glyma.16G014400	brown
Glyma.17G008400	brown
Glyma.17G037600	brown
Glyma.18G058900	brown
Glyma.18G102800	brown
Glyma.19G021600	brown
Glyma.19G027200	brown

Glyma.19G189100	brown
Glyma.19G208600	brown
Glyma.20G084500	brown
Glyma.02G103500	grey
Glyma.07G214700	grey
Glyma.01G004200	turquoise
Glyma.01G082200	turquoise
Glyma.01G139600	turquoise
Glyma.01G163900	turquoise
Glyma.01G169700	turquoise
Glyma.01G178200	turquoise
Glyma.01G188900	turquoise
Glyma.01G194100	turquoise
Glyma.01G222500	turquoise
Glyma.01G228500	turquoise
Glyma.01G228700	turquoise
Glyma.01G239600	turquoise
Glyma.02G045700	turquoise
Glyma.02G073000	turquoise
Glyma.02G208400	turquoise
Glyma.02G226300	turquoise
Glyma.02G236500	turquoise
Glyma.02G236900	turquoise
Glyma.02G259000	turquoise
Glyma.02G267800	turquoise
Glyma.02G307300	turquoise
Glyma.03G004200	turquoise
Glyma.03G027400	turquoise
Glyma.03G088400	turquoise
Glyma.03G143700	turquoise
Glyma.03G209900	turquoise
Glyma.03G226500	turquoise
Glyma.03G255400	turquoise
Glyma.03G257700	turquoise
Glyma.04G104900	turquoise
Glyma.04G173300	turquoise
Glyma.04G184900	turquoise
Glyma.04G219200	turquoise
Glyma.04G235700	turquoise
Glyma.04G238800	turquoise
Glyma.04G242700	turquoise
Glyma.05G028800	turquoise
Glyma.05G204000	turquoise
Glyma.06G046400	turquoise

Glyma.06G074300	turquoise
Glyma.06G105900	turquoise
Glyma.06G120600	turquoise
Glyma.06G124900	turquoise
Glyma.06G129000	turquoise
Glyma.06G190900	turquoise
Glyma.06G230300	turquoise
Glyma.06G235500	turquoise
Glyma.06G254700	turquoise
Glyma.06G303600	turquoise
Glyma.07G069300	turquoise
Glyma.07G175000	turquoise
Glyma.07G199900	turquoise
Glyma.07G202300	turquoise
Glyma.07G249100	turquoise
Glyma.07G250900	turquoise
Glyma.07G265600	turquoise
Glyma.08G095600	turquoise
Glyma.08G113700	turquoise
Glyma.08G138800	turquoise
Glyma.08G267400	turquoise
Glyma.08G297300	turquoise
Glyma.09G036300	turquoise
Glyma.09G046500	turquoise
Glyma.09G248900	turquoise
Glyma.09G273100	turquoise
Glyma.10G058200	turquoise
Glyma.10G090600	turquoise
Glyma.10G211400	turquoise
Glyma.10G212200	turquoise
Glyma.10G292200	turquoise
Glyma.11G011500	turquoise
Glyma.11G047800	turquoise
Glyma.11G053300	turquoise
Glyma.11G064000	turquoise
Glyma.11G073600	turquoise
Glyma.11G078700	turquoise
Glyma.11G079600	turquoise
Glyma.11G140900	turquoise
Glyma.11G199400	turquoise
Glyma.12G032500	turquoise
Glyma.12G064400	turquoise
Glyma.12G100300	turquoise
Glyma.12G161200	turquoise

Glyma.12G193000	turquoise
Glyma.12G194500	turquoise
Glyma.12G228900	turquoise
Glyma.13G007000	turquoise
Glyma.13G036600	turquoise
Glyma.13G040500	turquoise
Glyma.13G057400	turquoise
Glyma.13G059200	turquoise
Glyma.13G063600	turquoise
Glyma.13G117700	turquoise
Glyma.13G145000	turquoise
Glyma.13G173500	turquoise
Glyma.13G176100	turquoise
Glyma.13G176400	turquoise
Glyma.13G270800	turquoise
Glyma.13G277400	turquoise
Glyma.13G334800	turquoise
Glyma.13G339300	turquoise
Glyma.13G363500	turquoise
Glyma.14G005700	turquoise
Glyma.14G030400	turquoise
Glyma.14G072700	turquoise
Glyma.14G118200	turquoise
Glyma.14G124300	turquoise
Glyma.14G205200	turquoise
Glyma.14G205700	turquoise
Glyma.14G222100	turquoise
Glyma.15G039700	turquoise
Glyma.15G054700	turquoise
Glyma.15G141200	turquoise
Glyma.15G154000	turquoise
Glyma.16G035000	turquoise
Glyma.16G058800	turquoise
Glyma.16G123700	turquoise
Glyma.16G138200	turquoise
Glyma.16G149300	turquoise
Glyma.16G154300	turquoise
Glyma.16G175600	turquoise
Glyma.16G175900	turquoise
Glyma.17G025200	turquoise
Glyma.17G032800	turquoise
Glyma.17G042100	turquoise
Glyma.17G098000	turquoise
Glyma.17G169700	turquoise

Glyma.18G120300	turquoise
Glyma.18G124600	turquoise
Glyma.18G143600	turquoise
Glyma.18G195700	turquoise
Glyma.18G216000	turquoise
Glyma.18G234800	turquoise
Glyma.19G027100	turquoise
Glyma.19G030500	turquoise
Glyma.19G121700	turquoise
Glyma.19G126000	turquoise
Glyma.19G182300	turquoise
Glyma.19G223500	turquoise
Glyma.19G253000	turquoise
Glyma.19G256200	turquoise
Glyma.20G103200	turquoise
Glyma.20G107300	turquoise
Glyma.20G114200	turquoise
Glyma.20G179500	turquoise
Glyma.20G241500	turquoise
Glyma.20G241700	turquoise

Table S8. Primers used in this study

Primer Name	Sequence(5' to 3')
QGmACC1-F	AACACTTCTAAGACAGCCGACAAC
QGmACC1-R	CAACTCCTCCATTGCAGCCATC
QGmACC2-F	TTCCGCCTGAAAGTTCCTTGATTAC
QGmACC2-R	CTTGCCACTGCTTCCTCTGTTG
QGmACC3-F	ACCTCCTCTGATTCCTGCTTCTG
QGmACC3-R	GGTACTGGGTCTACTGGCTTCG
QGmACC4-F	TGTGTCGGGAGAGGTCATCAAG
QGmACC4-R	AACAAGTGCGTCACCATATCCAAC
QGmACC5-F	ACCAGCATCTACTCCAGCACCTAC
QGmACC5-R	GGGGCTTTTAAGAGGCGGAAGTG
QGmACC6-F	ATGGCGTCATTCACGATCCCTTG
QGmACC6-R	GCGTTCCGTTGCTGGGTCTG
QGmACC7-F	TTGCGGAGAATGCTGTGTTTGTTG
QGmACC7-R	TGTCTCTCTGGCAGTTGCTTTGTC
QGmACC9-F	GTCCGTTAGCTCGCCTTCTGTTC
QGmACC9-R	GCTCGCTCCCGCTAAGAAACTG
QGmACC10-F	TGCTGAGCCACCTTTCCTTGTTC
QGmACC10-R	AGTCCAGTTTCGTTCGCCATCTTC
QGmACC11-F	AAGATGGCGAACGAAACTGGACTG
QGmACC11-R	CGATGTTGACCCGCTGAATAGGAG
QGmACC12-F	TGCTGAGCCACCTTTCCTTGTTC
QGmACC12-R	AGTCCAGTTTCGTTCGCCATCTTC
QGmACC16-F	AGACATTGTGGAGTTGAAGCTGAAG
QGmACC16-R	GAGGAGGAGGTGGTGGTTGAG
QGmACC17-F	CATTTGTCAAGGTGGGAGATAAAGTG
QGmACC17-R	TCAGCCTCAATTTCATTCATCAGTTTC
QGmACC18-F	GGCTTTGGTATTAGAGGTCTGTGATG
QGmACC18-R	CTTGGAATAGGTGGTGGTGTAGTTG
QGmACC19-F	AATCAAAGAAGGGCAAGTCATAGGG
QGmACC19-R	TCTCCATAACCAACAGGCTCTCC
QGmACC20-F	CTACAACTTCTTCTTCGTCTTCTCTCC
QGmACC20-R	GCTTCCTGTTAAGGCACTGAATCC

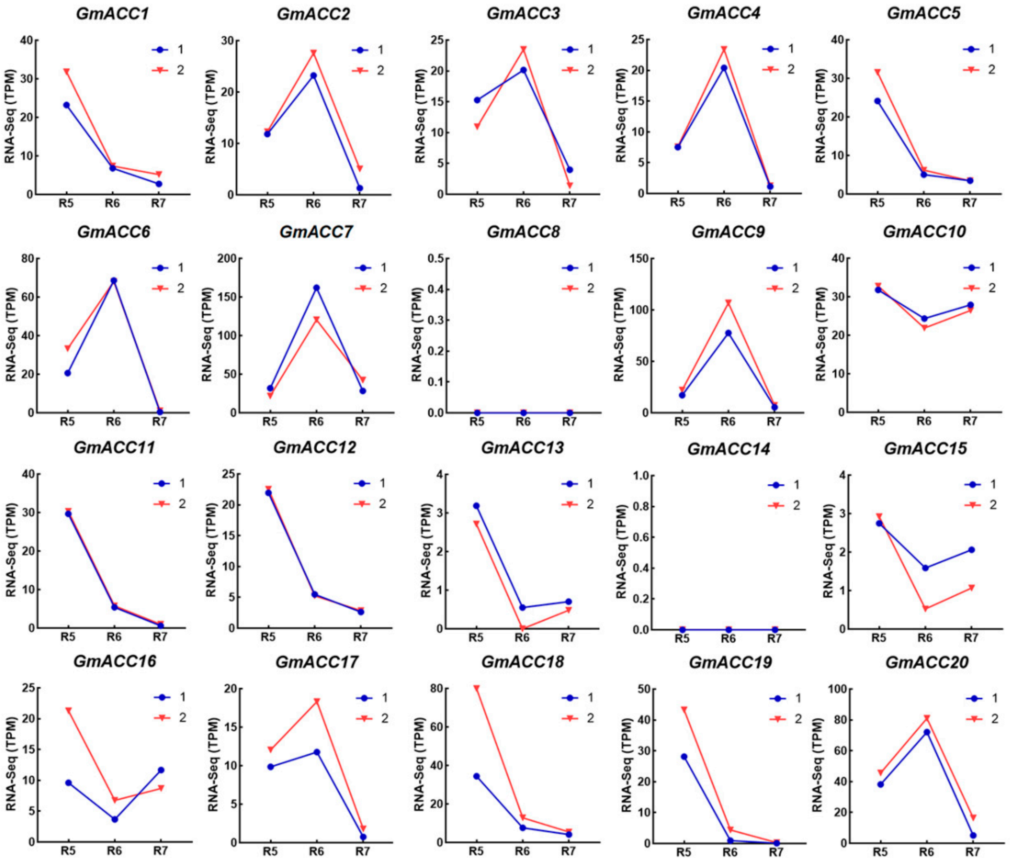

Figure S1. Seed development R5-R7 stage 20 *GmACCs* transcriptome sequencing data. The blue line represents the low isoflavone variety, and the red line represents the high isoflavone variety.
